# Supplementary material for: Breathing and Tilting: Mesoscale Simulations Illuminate Influenza Glycoprotein Vulnerabilities
Source: ACS Cent Sci. 2022 Dec 8;8(12):1646–63. doi: 10.1021/acscentsci.2c00981 (PMC9801513; doi:10.1021/acscentsci.2c00981)
Supplement: Supplementary file 11 — oc2c00981_si_012.pdf [file oc2c00981_si_012.pdf]

Supporting Information

Breathing and tilting: mesoscale simulations illuminate influenza glycoprotein vulnerabilities

Lorenzo Casalino[1], Christian Seitz[1], Julia Lederhofer[2], Yaroslav Tsybovsky[3], Ian A. Wilson[4], Masaru Kanekiyo[2], Rommie E. Amaro[1]*

[1]Department of Chemistry and Biochemistry, University of California San Diego, La Jolla, California 92093, United States
[2]Vaccine Research Center, National Institute of Allergy and Infectious Diseases, National Institutes of Health, Bethesda, MD, 20892, USA
[3]Electron Microscopy Laboratory, Cancer Research Technology Program, Frederick National Laboratory for Cancer Research sponsored by the National Cancer Institute, Frederick, MD 21702, United States
[4]Department of Integrative Structural and Computational Biology and the Skaggs Institute for Chemical Biology, The Scripps Research Institute, La Jolla, CA 92037, United States

*Correspondence to: ramaro@ucsd.edu

TABLE OF CONTENTS:

1. **Materials and Methods**
   1.1. Computational Methods
   1.2. Experimental Materials and Methods
2. **Supplementary Figures (S1 to S29)**
3. **Supplementary Tables (S1 to S9)**
4. **Captions for Supplementary Movies (S1 to S10)**
5. **Captions for Supplementary Data (S1 to S2)**
6. **Supplementary References**

1. Materials and Methods

1.1 Computational Methods

Mesoscale all-atom modeling of the influenza virus

In the work presented here, two glycosylated, whole-virion models of the H1N1 influenza A (H1N1) virus were built, simulated, and analyzed. The first corresponds to the strain A/swine/Shandong/N1/2009(H1N1) (hereafter referred to as H1N1-Shan2009), whereas the second one accounts for the A/45/Michigan/2015(H1N1) strain (hereafter referred to as H1N1-Mich2015). Both models are based on the unglycosylated H1N1-Shan2009 whole-virion model previously built by us and presented in Durrant et al.[1] The main difference lies in the addition of N-linked glycans to HA and NA glycoproteins, which substantially improved the biological relevance of the simulated constructs. Although we refer to Durrant et al.[1] for a detailed description of the original modeling of the individual proteins, assembly procedure of the whole-virion model, and molecular dynamics (MD) simulations of both the individual proteins (HA, NA, M2) and the whole-virion model, a summary is also provided in the following paragraph.

The unglycosylated, H1N1-Shan2009 whole-virion model constructed and simulated by Durrant et al.[1] comprises 236 HA trimers, 30 NA tetramers, and 11 M2 ion channels embedded in a 3-palmitoyl-2-oleoyl-dglycero-1-phosphatidylcholine (POPC) quasi-spherical lipid bilayer.[1] HA, NA and M2 were individually modeled and separately equilibrated (HA and NA) through MD simulations before being incorporated into the viral membrane.[1] We note that in Durrant et al.[1] glycans were not modeled. LipidWrapper[2] was used to create the spherical lipid bilayer starting from a large planar POPC-bilayer model generated with the CHARMM-GUI server.[3,4] As specified in reference 1,[1] slight differences in the leaflet densities are magnified by several times in the mesoscale systems, possibly creating large instabilities during simulation.[5] Although a bilayer made of only POPC represents an oversimplification of the actual membrane composition,[6] it has the advantage of minimizing such instabilities during simulations. This was the main reason behind the decision to build a POPC-only lipid bilayer. The M1 matrix proteins, coating the inner leaflet of the lipid bilayer, and the ribonucleoproteins contained inside of the virion were not modeled. As described in Amaro et al.[7], the shape of the virion and the distribution of the glycoproteins in the viral membrane were adopted from a point model of the virion exterior provided by collaborators, which was in turn derived from a cryoelectron tomography (cryoET) map of the influenza virus pleiomorphy.[8] Positioning of the glycoproteins and insertion of M2 channels in the lipid bilayer according to the point model was done with PyMolecule.[9,10] Full procedure, including handling of clashes, is described in references 1 and 7.[1,7] The whole-virion model was solvated inside and outside of the lipid bilayer with explicit TIP3P water molecules,[11] and ionized at 150 mM concentration with Na^+/Cl^-, tallying 160,653,271 atoms. All-atom MD simulations were performed on the Blue Waters supercomputer using NAMD2.10[12] and CHARMM36 all-atom additive force fields for protein,[13] lipids,[14,15] and ions.[16]

In the next paragraphs, we detail the system setup and all-atom MD simulations of the glycosylated H1N1-Shan2009 and H1N1-Mich2015 whole-virion models presented in this work.

System setup of the glycosylated H1N1-Shan2009 whole-virion model

UniProtKB accession codes for the sequences of HA and NA modeled in H1N1-Shan2009 are F2YI86 and F2YI87, respectively. HA was modeled in its uncleaved form (HA0), with residues ranging from 1 to 566. NA residues range from 1 to 469. We note that for HA and NA we adopted the absolute numbering system that starts with the first residue of the sequence in the corresponding UniProtKB entry. Sequences are reported in Figure S2 and Figure S3. The M2 construct[1] was based on the 2L0J structure,[17] tallying 41 residues (from 22 to 62) since N-terminal and C-terminal tails were not modeled.

Initial coordinates for the setup of the glycosylated whole-virion model of H1N1-Shan2009 were derived from the last frame of the all-atom MD simulation of the unglycosylated whole-virion model previously performed by us and briefly described in the previous subsection.[1] Specifically, we used the last frame obtained from the third simulation branch, which is depicted in green in the schematic reported in the supplementary information file of reference 1.[1] At that point of the simulation, corresponding to 69.74 ns, lipid-bilayer instabilities had been already resolved and the system had been steadily simulated without major issues. Our goal was to improve the simulated system by adding N-linked glycans to HA and NA while preserving the relaxed, MD-derived morphology of the virion, thus facilitating a smooth continuation of the simulation endeavor. For this reason, we retained the MD-derived coordinates of all the atoms of the system, including water molecules and ions, and then performed glycosylation of HA and NA accordingly (Figure S4). Specific oligo-mannose, hybrid, and complex N-linked glycans were identified to be linked to HA and NA, as described in the following paragraphs.

Each H1N1-Shan2009 HA monomer exhibits six N-linked sequons (Figure S5), of which one (N104) is located within the head and five (N28, N40, N293, N304, N498) within the stalk,[18] for a total of 18 potential N-linked glycans per trimer. It must be noted that N27 is also a potential glycosylation site; however, as reported in previous studies,[18,19] it is not glycosylated as N-X-T sequon at residue 28 is preferentially glycosylated over the N-X-S sequence at residue 27. N293 is located on the edge between the head and the stalk, but it is considered part of the stalk.[20,21] Several glycomics studies were used as a reference to build the glycosylation profile of HA,[18,22–24] which is shown in Figure S5. Instead, each H1N1-Shan2009 NA monomer presents eight N-linked sequons (Figure S5), of which four are located within the head (N88, N146, N235, N386) and four within the stalk (N50, N58, N63, N68),[18] for a total of 32 potential N-linked glycans per tetramer. Several glycomics studies were used as a reference to build the glycosylation profile of NA,[18,19,25] which is displayed in Figure S5.

Upon defining the respective glycosylation profiles, N-glycans were iteratively added to all the 236 HA trimers and 30 NA tetramers using the doGlycans tool.[26] VMD,[27] NAMD,[28] GROMACS[29] and the tools therein contained were also used in the glycosylation workflow for pulling each glycoprotein from the virion, assessing sequon accessibility, renaming residues, local minimization, and parameterization. HA and NA N-linked sequons are overall all highly occupied.[22,30,31] However, since glycans were added to a construct resulting from 69.74 ns of MD,[1] many sequons occurred to be sterically hindered by adjacent residues or not fully exposed to the

solvent as a result of the previous dynamics. Moreover, since the viral membrane was originally densely studded with unglycosylated HAs and NAs,[7,8] adding glycans to sequons located at the interface between adjacent glycoproteins was often not possible. For these reasons, all the sequons were subjected to a preliminary screening to evaluate their accessibility. The accessible surface area (ASA) of the asparagine side chain's ND2 atom was calculated to determine whether a sequon was accessible in our initial model. Both scenarios, where HD21 or HD22 were removed from ND2, were considered (Figure S4A). ASA was calculated with the *measure sasa* command implemented in VMD,[27] combined with in-house scripts. A sphere of radius 10 Å, approximating the size of the basic $(GlcNAc)_2(Man)_3$ N-core present in all glycans (Figure S4B), was used as a probe for the ASA screening. If the ASA of the putative asparagine's ND2 atom was larger than 10 Å^2, then a glycan would be tentatively linked (Figure S4C). Clashes with surrounding amino acids or other glycans were subsequently examined and solved, first by performing a local minimization with NAMD2.13[28] and then by eliminating the clashing glycan if the clashes could not be solved with minimization. Once the glycosylated glycoproteins were positioned back in the viral membrane, possible clashes arising between the glycans and the neighboring (glycosylated) glycoproteins were examined, resulting in the depletion of the clashing glycans. This further reduced the total number of glycans that were added. A total of 1521 glycans (29.2% of all the available sequons), among which 1277 were linked to HA (30.0%) and 244 to NA (25.4%) (Figure S5). We note that we could only populate 29 out of the 708 available N104 sequons within the HA head. This low number is due to the overall limited accessibility–in our system–of residue N104, which is occluded by neighboring residues. A similar scenario related to the modeling of HA's N104 glycosylation site is also reported in reference 18.[18]

Next, we adjusted the orientation of the 11 M2 ion channels within the lipid bilayer, so that the four N-termini of each M2 tetrameric assembly were directed toward the outside of the virion (extravirion), whereas the four C-termini were directed toward the inside of the virion (intravirion).[32,33] This operation was necessary since the M2 channels had been positioned upside down in the original system presented in Durrant et al.[1] No alterations in the composition and the coordinates of the POPC lipid bilayer were done with respect to the original system.[1] The resulting glycosylated H1N1-Shan2009 virion construct, comprising 236 glycosylated HA homo-trimers, 30 glycosylated NA homo-tetramers, and 11 M2 homo-tetramers embedded in the POPC lipid bilayer, was parameterized using NAMD's PSFGEN[28] and CHARMM36 all-atom additive force fields for protein,[13] lipids,[14,15] glycans,[34] and ions.[16] As mentioned above, explicit TIP3 water molecules[11] located inside and outside of the lipid bilayer, Na^+ and Cl^- ions, as well as Ca^{2+} ions bound to the NA heads, were retained from the original unglycosylated system.[1] The addition of glycans altered the overall electric charge of the solute, thus requiring tweaking of the total number of Na^+ and Cl^- ions to ensure electrical neutrality. These modifications did not impact the overall ionic strength, which remained at ~150 mM. The following minimization steps solved eventual clashes between newly added glycans and pre-existing water molecules and ions. The total number of atoms for the final system is 160,919,594, with an orthorhombic periodic cell of 1142 Å × 1194

Å × 1155 Å. After generating suitable files for mesoscale simulations with NAMD,[35] the system was ready to undergo all-atom MD simulations.

System setup of the glycosylated H1N1-Mich2015 whole-virion model

UniProtKB accession codes for the sequences of HA and NA modeled in H1N1-Mich2015 are A0A144YDV8 and A0A0X9QTS2, respectively. HA was modeled in its uncleaved form (HA0), with residues ranging from 1 to 566. NA residues range from 1 to 469. We note that for HA and NA, we adopted the absolute numbering system that starts with the first residue of the sequence in the corresponding UniProtKB entry. Sequences are reported in Figure S2 and Figure S3. The M2 construct[1] was based on the 2L0J structure,[17] tallying 41 residues (from 22 to 62) since N-terminal and C-terminal tails were not modeled.

Building on the same initial coordinates obtained from the MD simulation of the unglycosylated virion,[1] the system setup for H1N1-Mich2015 was conducted in the same way as described for H1N1-Shan2009. The main challenge was introducing the H1N1-Mich2015 point mutations occurring in HA and NA. All the mutations within HA (15 per monomer) and NA (14 per monomer) were modeled onto the initial construct using NAMD's PSFGEN[28] *mutate* command and the CHARMM36 amino acid topologies to build side chain rotamers.[13] The mutations are listed and displayed in Figure S6. No mutations were introduced to M2 ion channels, which were treated and positioned in the lipid bilayer in the same way as in H1N1-Shan2009. The resulting, mutated models of HA and NA were then subjected to the same glycosylation procedure adopted for H1N1-Shan2009 and described above.

S179N mutation introduced a second glycan within the HA head (Figure S7), in addition to the already existent N104. Therefore, each H1N1-Mich2015 HA monomer exhibits seven N-linked sequons (Figure S8), of which two (N104, N179) are located within the head and five (N28, N40, N293, N304, N498) within the stalk,[18] for a total of 21 potential N-linked glycans per trimer. The same glycoprofile as in H1N1-Shan2009 was adopted.[18,22–24] The new glycan linked to N179 was modeled as a complex tri-antennary (Figure S8). N386K mutation ablated the N386 sequon within the NA head (Figure S7). Therefore, each H1N1-Mich2015 NA monomer presents seven N-linked sequons (Figure S8), of which three are located within the head (N88, N146, N235) and four within the stalk (N50, N58, N63, N68),[18] for a total of 28 potential N-linked glycans per tetramer. The same glycoprofile as in H1N1-Shan2009 was adopted except for the deleted N386 glycan (Figure S8).[18,19,25]

A total of 1791 glycans (30.9 % of all the available sequons) were added to H1N1-Mich2015, among which 1626 were linked to HA (32.8%) and 165 to NA (19.6%) (Fig. S8). We note that also for H1N1-Mich2015 we could populate a low number of N104 sequons within the HA head (33 of 708). Instead, we could populate 351 sequons out of 708 available (49.6%) for the new N179 glycan within the HA head (Figure S8).

Similar to H1N1-Shan2009, the resulting glycosylated H1N1-Mich2015 virion construct was parameterized using NAMD's PSFGEN[28] and CHARMM36 all-atom additive force fields for protein,[13] lipids,[14,15] glycans,[34] and ions.[16] As for H1N1-Shan2009, explicit TIP3P water

molecules[11] located inside and outside of the lipid bilayer, Na^+ and Cl^- ions, as well as Ca^{2+} ions bound to the NA heads, were retained from the original unglycosylated system.[1] The amino acid mutations and the addition of glycans altered the overall electric charge of the solute, thus requiring further tweaking of the total number of Na^+ and Cl^- ions to establish electrical neutrality. These modifications did not impact the overall ionic strength, which remained at ~150 mM. The following minimization steps solved eventual clashes involving mutated residues or between newly added glycans and pre-existing water molecules and ions. The total number of atoms for the final system is 160,981,954, with an orthorhombic periodic cell of 1138 Å × 1189 Å × 1150 Å. After generating suitable files for mesoscale simulations with NAMD,[35] the system was ready to undergo all-atom MD simulations.

All-atom molecular dynamics simulations

All-atom MD simulations of H1N1-Shan2009 and H1N1-Mich2015 were performed on the TITAN supercomputer at Oak Ridge National Laboratory (ORNL) and the NSF Blue Waters supercomputer at the University of Illinois at Urbana-Champaign, respectively, using the CUDA memory-optimized version of NAMD 2.13.[12] The same simulation protocol was adopted for both H1N1-Shan2009 and H1N1-Mich2015. CHARMM36 all-atom additive force fields were used to describe proteins,[13] lipids,[14,15] glycans,[34] and ions.[16] Simulations were performed in explicit water using the TIP3P model.[11]

Preliminary tests were initially run to assess the stability of the systems, familiarize with the computing facilities, and debug possible errors. Then, the systems were initially minimized in two consecutive cycles of 15,000 steps, each using the NAMD's conjugate gradient energy approach. During the first cycle, the position of protein backbone atoms and of POPC P atoms within 15 Å of the M2 ion channels was kept harmonically restrained at 100 (kcal/mol)/Å^2. We note that the orientation of M2 ion channels was modified with respect to our previous work,[1] thus requiring more caution. We also remark that the initial coordinates for water molecules, ions, lipids, and proteins (except for M2) were retained from a previous MD simulation as described in the previous sections,[1] thus being already equilibrated. During the second minimization cycle, the harmonic restraints on the protein backbone atoms were released, whereas the harmonic restraints on the POPC P atoms within 15 Å of M2 ion channels were decreased to 10 (kcal/mol)/Å^2. After minimization, the systems were gradually heated up with no restraints from 0 K to 298 K in 220 ps using isothermal–isobaric conditions (NPT ensemble). The temperature was controlled using the Langevin thermostat[36] with a damping coefficient of 5/ps, whereas the Nosé-Hoover Langevin piston[37,38] was used to maintain the pressure at 1.01325 bar. A time-step of 2 fs was used. Next, the systems were equilibrated for 0.5 ns under the NPT ensemble. Temperature (298 K) and pressure (1.01325 bar) control were achieved again using the Langevin thermostat[36] with a damping coefficient of 5/ps, and the Nosé-Hoover Langevin piston.[37,38] During this phase, harmonic restraints at 100 (kcal/mol)/Å^2 were applied again to POPC P atoms within 15 Å of M2 ion channels and gradually released to 10 (kcal/mol)/Å^2.

Finally, the systems were submitted to productive MD simulations in NPT conditions using the same barostat and thermostat as above to achieve pressure (1.01325 bar) and temperature (298 K) control. Langevin damping coefficient was set to 1/ps during productive MD. One continuous replica was performed for each system using a time-step of 2 fs. To preserve the overall geometry of the virion models in the absence of the interior component, we applied a harmonic position restraint of 20 (kcal/mol)/Å^2 to every 20th inner-leaflet P atom. A similar protocol was adopted in our previous simulation of the unglycosylated virion,[1] where instead every 10th POPC head group was kept fixed. Non-bonded interactions (van der Waals and short-range electrostatic) were calculated at each timestep using a cutoff of 12 Å and a switching distance of 10 Å. All simulations were performed using periodic boundary conditions, employing the particle-mesh Ewald method[39] with a grid spacing of 2.1 Å to evaluate long-range electrostatic interactions every three timesteps. SHAKE algorithm[40] was adopted to keep the atomic bonds involving hydrogens fixed with the NAMD option *rigidbonds all*. Frames were saved every 30,000 steps (60 ps).

For H1N1-Shan2009, we collected a total of ~441.78 ns (7363 frames) continuous productive MD (Movie S1). Simulations were performed with the CUDA memory-optimized version of NAMD 2.13[12] running on 4,096 physical nodes (28,672 processors) on the TITAN supercomputer, with an average benchmark of ~13.83 ns/day. A total of ~17 TB of data were generated, including stripped trajectories.

For H1N1-Mich2015, we collected a total of 424.98 ns (7083 frames) continuous productive MD (Movie S2). Simulations were performed with the CUDA memory-optimized version of NAMD 2.13[12] running on 4,096 physical nodes (114,688 processors) on the Blue Waters supercomputer, with an average benchmark of ~13.78 ns/day. A total of ~16 TB of data was generated including stripped trajectories.

Root Mean Squared Deviation (RMSD) analysis of HA and NA

For both strains, each of the 236 HA trimeric and 30 NA tetrameric trajectories was separately aligned using as a reference the respective initial coordinates of all the backbone atoms (C, N, O, Cα). Then, for each of the HA trimers/NA tetramers, we calculated the RMSD of the backbone atoms using the *measure rmsd* command implemented in VMD.[27] Subsequently, we computed the mean and standard deviation of the RMSD values obtained at each frame across all the 236 HA trimers / 30 NA tetramers, generating, respectively, the averaged RMSD profiles for HA and NA (Figure S9).

Root Mean Squared Fluctuation (RMSF) analysis of HA and NA residues

We performed the calculation of Cα-atom RMSF to probe the average mobility of HA and NA residues. Before the calculation of Cα-atom RMSF, rotation and translation motions of each individual HA trimer and NA tetramer were removed through specific alignments. In detail, two sets of aligned trajectories, based on different residue masks, were generated for each of the 236 HA trimers and 30 NA tetramers of both strains. For each of the HA trimers, the first set of alignment was performed using as a reference the respective initial coordinates of all the backbone

atoms (C, N, O, Cα) of the ectodomain region (residues 18 to 503) of the respective glycoprotein, whereas the second set was based on the region comprising the transmembrane domain (TMD) (residues 529 to 549). The former served for the calculation of RMSF of residues 1 to 503, the latter for the calculation of RMSF of residues 504 to 566. For each of the NA tetramers, the first set of alignment was performed using as a reference the respective initial coordinates of all the backbone atoms (C, N, O, Cα) of the head region (residues 96 to 461) of the respective glycoprotein, whereas the second set was based on the region comprising the stalk and the TMD (residues 7 to 81). The former served for the calculation of RMSF of residues 96 to 469, the latter for the calculation of RMSF of residues 1 to 95. This procedure was performed due to peculiar structures of HA and NA that present domains undergoing tilting motions during the dynamics, i.e., HA ectodomain tilting and NA head tilting. If we had performed a single alignment based on the backbone atoms of the whole structure, the extensive motions of the HA ectodomain and NA head, which are larger in size and account for most of the residues, would have biased the RMSF values of the complementary region encompassing the hinge linker (both for HA and NA), the stalk (for NA), the TMD, and the cytosolic tail.

For each trajectory of both sets and for each monomer, we calculated the RMSF of the Cα atoms of the residues belonging to the respective region using the *measure rmsf* command implemented in VMD.[27] All the frames were used for the calculation. We then combined the RMSF values obtained for the two regions into a single RMSF profile for each monomer. Finally, we computed the mean and standard deviation of the RMSF values obtained for each Cα atom across all the 708 HA monomers / 120 NA monomers, generating the respectively averaged RMSF profiles for HA and NA (Figure S10).

<u>Positional fluctuations of HA's and NA's TMD and ectodomain center of masses</u>

For both H1N1-Shan2009 and H1N1-Mich2015, we calculated the RMSF of HA's and NA's TMD center of mass (COM) and ectodomain COM using the first frame as a reference. The analysis was performed using in-house tcl scripts within the VMD framework.[27] We note that the aim of this analysis was estimating the average positional fluctuations of the TMD COM and ectodomain COM of each glycoprotein within the virion. Therefore, only the global rotation and translation motions of the whole virion were removed and not those of each individual glycoprotein within the virion. Global rotation and translation motions of the whole virion coordinates during dynamics were removed by aligning the whole-virion trajectory using as reference the coordinates of all the protein backbone atoms (C, N, O, Cα) at the initial frame. The COM of HA TMD was defined using the coordinates of backbone atoms of residues 529 to 549. The COM of HA ectodomain was defined using the coordinates of backbone atoms of residues 1 to 528. The COM of NA TMD was defined using the coordinates of backbone atoms of residues 7 to 29. The COM of NA ectodomain was defined using the coordinates of backbone atoms of residues 30 to 469.

At each frame of the whole-virion trajectory, we calculated, for each glycoprotein, the squared positional fluctuation of both COMs (i.e., ectodomain COM and TMD COM) with respect

to their initial position (reference). Next, always for each glycoprotein, we averaged the squared fluctuations of the COMs and calculated the square root of the averages, obtaining the respective RMSF value.

NA head-tilt angle

The NA head-tilt angle was calculated for all 30 NA tetramers at each frame of both virion trajectories using in-house tcl scripts within the VMD framework.[27] The NA head-tilt angle is defined by the principal axis of the tetrameric upper stalk region (residues 50 to 78) and the vector joining residue 78 at the top of the stalk with the COM of the tetrameric head (residues 91 to 469). Only Cα atoms were considered to calculate the COMs. The distribution of NA head-tilt angle values was computed through kernel density estimation using Gaussian kernels with gnuplot plotting program.[41]

HA ectodomain-tilt angle

The HA ectodomain-tilt angle was calculated for all 236 HA trimers at each frame of both virion trajectories using in-house tcl scripts within the VMD framework.[27] The HA ectodomain-tilt angle is defined by the principal axis of the trimeric TMD (residues 529 to 549) and the vector joining residue 529 at the top of the TMD with the COM of the trimeric ectodomain's long α-helices (LαHs) (residues 419 to 471). Only Cα atoms were considered to calculate the COMs. The distribution of HA ectodomain-tilt angle values was computed through kernel density estimation using Gaussian kernels with gnuplot plotting program.[41]

HA head breathing

The extent of HA head breathing was estimated by calculating the distance between the COM of each monomeric HA head (residues 66 to 286) and the COM of the apical residues of the three LαHs. The apical residue of each LαH corresponds to residue 419, and the COM was calculated based on the three residues 419 as in the trimeric assembly. Only Cα atoms were considered to calculate the COMs. This distance was calculated for all 708 HA monomers at each frame of both virion trajectories using in-house tcl scripts within the VMD framework.[27] The distribution of HA head–LαHs distance values was computed through kernel density estimation using Gaussian kernels with gnuplot plotting program.[41]

NA head breathing

The extent of NA head breathing was estimated by calculating the inter-protomer distances between the four NA head COMs (residues 96 to 461). Only Cα atoms were considered to calculate the COMs. Considering the four NA heads within each tetramer, a total of six distances have been monitored for each NA tetramer (Figure S27), at each frame of both virion trajectories, using in-house tcl scripts within the VMD framework.[27] The distribution of the values of the inter-protomer distances was computed through kernel density estimation using Gaussian kernels with gnuplot plotting program.[41]

FluA-20 epitope antibody accessible surface area (AbASA)

Antibody accessible surface area (AbASA) of the FluA-20[42] epitope, located on the HA head domain trimer interface, was calculated using the *measure sasa* VMD command,[27] an implementation based on the Shrake and Rupley algorithm,[43] along with in-house tcl scripts to handle data. The following HA residues were considered for the epitope: residues 109, 230, 232-237, 243. An 18.6 Å probe radius, which approximately encloses the FluA-20 Fab's variable domains (see Figure 5 in the main text), was used. ASA evaluations were conducted on the HA trimer showing the highest degree of head domain breathing, at every frame of the trajectory.

Evaluation of glycoprotein–glycoprotein interactions

For both virion simulations, interactions between glycoproteins were evaluated using the *measure contacts* implemented in VMD[27] along with in-house tcl scripts to handle data.

For each glycoprotein, we first pre-computed a list of neighbor glycoproteins using a cutoff of 75 Å. Then, always for each glycoprotein, we assessed the number of connections formed with any of the glycoproteins included in its respective neighbor list every 10th frame (i.e., every 0.6 ns) of the virion trajectory. At each evaluation, a "connection" was considered formed and counted as one when any protein or glycan heavy atoms of the screened glycoprotein were detected to be within 5 Å of any protein or glycan heavy atoms of the screened neighbor glycoprotein. Only heavy atoms belonging to the ectodomains were considered for the analysis. Importantly, if glycoprotein "A" was, for example, found to form a connection with glycoprotein "B", that connection was counted one time for glycoprotein "A" and one time also for glycoprotein "B". Relative fractions of glycoproteins forming respectively 0, 1, 2, 3, 4, or 5 connections at each evaluation were subsequently computed with respect to the total number of glycoproteins (266). Results are shown in Figure 7 for all the glycoproteins, i.e., HAs + NAs, and in Figure S26 for HA and NA separately.

Within the same workflow, we then evaluated the formation of macroclusters of glycoproteins. At each assessment, we calculated the number of glycoproteins that were part of a macrocluster using the same cutoff of 5 Å between protein/glycan heavy atoms. If glycoprotein "A" was, for example, found to form a connection with glycoprotein "B," and, glycoprotein "B" was in turn found to establish at the same time a connection with glycoprotein "C," the three glycoproteins A, B, and C were considered as part of a macro-cluster of 3, regardless of the presence of a direct connection between A and C. Results are shown in Figure S27.

Next, we evaluated the nature of the connections formed by each glycoprotein. The connections were broken down into three categories depending on the residues used by each glycoprotein to form such connections: i) "glycan-only" if only glycan residues were used to form the connection; ii) "protein-only" if only protein residues were used to form the connection; "protein and glycan" if both protein and glycan residues were used at the same time to interact with the screened neighbor glycoprotein. Relative frequencies of "glycan-only", "protein-only", and "protein and glycan" types of connection were subsequently computed with respect to the total number of counted connections. Results are shown in Figure 7 in the main text.

Finally, if glycans were involved in a connection between two glycoproteins, for each glycoprotein we counted the number of stalk/head glycans involved in the connection, respectively. Relative frequencies of the interacting stalk/head glycans were subsequently calculated for HA and NA with respect to the respective total number of stalk/head glycans in both strains. Results are shown in Figure S28.

Evaluation of conditional probabilities between HA and NA tilting/breathing and interprotein connectivity

For each motion investigated in this work, namely NA head tilting, HA ectodomain tilting, and HA head breathing, we calculated the conditional probability that a glycoprotein (either NA or HA) exhibits a certain degree of tilting/breathing during the dynamics given that it forms a connection with one or more neighboring glycoproteins. Here, the conditional event, i.e., B, the formation of one or more connections during the dynamics, should account for the interprotein connectivity. Therefore, with this analysis, we investigate whether the interprotein connectivity somehow affects the probability of achieving a certain degree of tilting/breathing. In order to calculate such conditional probability, we used the following formula:

$$P(A|B) = P(A \cap B) / P(B)$$

Where:

- P(A) is the probability that a glycoprotein (HA or NA) tilts/breathes by a certain amount during the dynamics with respect to the initial frame.
- P(B) is the probability that the glycoprotein forms one or more connections during the dynamics. P(B) must be > 0.
- $P(A \cap B)$ is the probability at which the events A and B occur together.
- P(A|B) is the probability of event A occurring if event B has happened.

P(A), P(B) and $P(A \cap B)$ were determined by counting the number of frames where events A, B, and $A \cap B$ occurred, respectively, and dividing it by the total number of screened frames. H1N1-Shan2009 and H1N1-Mich2015 trajectories were analyzed with a stride of 10. To assess whether event A occurred or not for HA ectodomain tilting and NA head tilting, we evaluated, for each HA/NA, the tilting angle at the examined frame with respect to the tilt angle at frame 0 and computed the difference, namely (*tilt-angle_frame_n – tilt-angle_frame_0*). If the obtained difference value was within a certain (positive) range, then event A would be counted as occurred for the evaluated range of values (see Tables S4–S9). Instead, to assess whether event A occurred or not for HA head breathing, we evaluated, for each HA trimer, the breathing distance of each monomer at the screened frame with respect to the respective breathing distance at frame 0 and computed the difference, namely (*breathing-distance_frame_n – breathing-distance_frame_0*). If the obtained difference value for at least one monomer within the trimer was within a certain (positive) range, then event A would be counted as occurred for the evaluated range of values (see

Tables S4–S9). To assess whether event B occurred or not we evaluated the number of connections made by the screened glycoprotein at each analyzed frame. If the number of connections was equal or larger than one, then event B would be counted as occurred. To assess whether event A∩B occurred or not, we evaluated whether event A and B occurred at the same time or not. If true, then event A∩B would be counted as occurred.

The absolute error of P(A) was calculated as *P(A) / sqrt((Count of number of times A was observed) – 1)*. The same was done for the absolute errors of P(B) and P(A∩B).

The absolute error of P(A|B) was calculated as $E_R(A|B) \times P(A|B)$, where $E_R(A|B)$ is the relative error of P(A|B).

The relative error of P(A|B), namely $E_R(A|B)$, was calculated as $sqrt(E_R(A)^2 + E_R(B)^2 + E_R(A\cap B)^2)$, where $E_R(A)$ is the relative error of P(A), $E_R(B)$ is the relative error of P(B), and $E_R(A\cap B)$ is the relative error of P(A∩B).

The relative error of P(A), namely, $E_R(A)$ was calculated as *E(A) / P(A)*. The same was done to calculate $E_R(B)$ and $E_R(A\cap B)$.

Next, we also estimated in a similar way the opposite conditional probability (P(B|A)) that a glycoprotein (either NA or HA) forms a connection with one or more glycoproteins, given that it exhibits a certain degree of tilting/breathing during the dynamics. Here, the conditional event is A, i.e., the degree of tilting/breathing achieved during the dynamics. Therefore, with this analysis, we investigate whether tilting/breathing affects the probability of HA/NA forming one or more connections. In order to calculate such conditional probability, we used the following formula:

$$P(B|A) = P(A\cap B) / P(A)$$

Where:

- P(B) is the probability that the glycoprotein forms one or more connections during the dynamics.
- P(A) is the probability that a glycoprotein (HA or NA) tilts/breathes by a certain amount during the dynamics with respect to the initial frame. P(A) must be > 0.
- P(A∩B) is the probability at which the events A and B occur together.
- P(B|A) is the probability of event B occurring if event A has happened.

Events A, B, and A∩B were counted as above, and errors were computed as described above.

<u>Markov State Models (MSMs)</u>

The first step was the preparation of individual NA tetrameric (30) and HA trimeric (236) trajectories, which were generated from the whole-virion MD simulations of H1N1-Shan2009 and H1N1-Mich2015, respectively. Glycans were not included in the generated trajectories because a common topology, i.e., equal for all the NA tetramers and HA trimers, respectively, was necessary to load all the trajectories in the MSM workflow. However, the impact of glycans on the

glycoproteins remains accounted for as the trajectories were generated from the simulations of the glycosylated whole-virions.

NA head tilting. The MD simulation trajectories were imported into the PyEMMA2 software[44] for MSM analysis. The MSM of the NA head tilting was created as follows. We used two features; the first was defined as the shortest distance between the COM of the head and the COM of the center of the stalk, while the second feature was defined as the shortest distance between the COM of the head and the COM of the bottom of the stalk. The exact residues defining these COM selections are given in the accompanying Jupyter notebook (Data S1). With these two features, we ran a time-lagged independent component analysis (TICA) as is commonly used on MSMs with multiple features[45–47] using a lag time of 100 steps. Our step size is 0.06, so 100 steps are equivalent to 6 ns. We ran k-means clustering[48] of the two features, selecting 300 clusters. From our implied timescale plots[49,50] we selected an MSM lag time of 250 steps (Figure S12). We then created Bayesian Markov state models, selecting two macrostates for each MSM. Using these parameters, we created MSMs and validated our two-state systems through Chapman-Kolmogorov (CK) tests[51–54] at a confidence interval of 95% (Figure S13). The estimated and predicted lines overlapped out to 750 steps (3x the MSM lag time number), which meant these MSMs are statistically valid.[51] We then used Robust Perron Cluster Center Analysis (PCCA++) to cluster the first two eigenvectors of the MSM transition matrices to cluster the states.[55] The first macrostate in the H1N1-Shan2009 strain MSM (untilted) had a stationary distribution of 51.4% of the total population, while the second macrostate (tilted) had a distribution of 48.6%. The first macrostate in the H1N1-Mich2015 strain MSM (untilted) had a stationary distribution of 44.3% of the total population, while the second macrostate (tilted) had a distribution of 55.7%. Finally, we extracted NA structures from the two macrostates per strain by creating a 99.99999% probability cutoff for the H1N1-Shan2009 strain macrostates and a 99.999999% probability cutoff for the H1N1-Mich2015 strain macrostates. This means that the NA structures (microstates) that we selected have a >99.99999% probability of residing in the macrostate state that we think they are in through the PCCA++ fuzzy spectral clustering method. Confirmation of which microstates corresponded to which conformations was performed through the MDTraj software program.[56]

HA ectodomain tilting. The MD simulation trajectories were imported into the PyEmma2 software[44] for MSM analysis. The MSM of the HA ectodomain tilting was created as follows. We used two features, defined as the distance between the COM of the head and the center of the HA stalk, and the distance between the HA monomer head tips and the bottom of the stalk. The exact residues defining these COM selections are given in the accompanying Jupyter notebook. With these two features, we ran TICA[45–47] using a lag time of 25 steps. Our step size is 0.06, so 25 steps are equivalent to 1.5 ns. We ran k-means clustering[48] of the three features, selecting 300 clusters. From our implied timescale plots[49,50] we selected an MSM lag time of 50 steps (Figure S18). We then created Bayesian Markov state models, selecting two macrostates for each MSM. Using these parameters, we created MSMs and validated our two-state systems through CK tests[51–54] at a confidence interval of 95% (Figure S19). The estimated and predicted lines overlapped out to 150 steps (>3x the MSM lag time number), which meant these MSMs are statistically valid.[51] We then

used PCCA++ to cluster the first two eigenvectors of the MSM transition matrices to cluster the states.[55] The first macrostate in the H1N1-Shan2009 strain MSM (tilted) had a stationary distribution of 6.3% of the total population, while the second macrostate (tilted) had a distribution of 93.7%. The first macrostate in the H1N1-Mich2015 strain MSM (tilted) had a stationary distribution of 6.2% of the total population, while the second macrostate (untilted) had a distribution of 93.8%. Finally, we extracted HA structures from the two macrostates per strain by creating a 99.999999% probability cutoff for all macrostates. This means that the HA structures (microstates) that we selected have a 99.999999% probability of residing in the macrostate state that we think they are in through the PCCA++ fuzzy spectral clustering method. Confirmation of which microstates corresponded to which conformations was performed through the MDTraj software program.[56]

HA head breathing. The MD simulation trajectories were imported into the PyEMMA2 software[44] for MSM analysis. The HA head breathing MSM was created as follows. We used one feature, defined as the smallest mean distance of the monomers to each other. The exact residues defining this feature are given in the accompanying Jupyter notebook. With this one feature, we ran TICA[45–47] using a lag time of 100 steps to properly format the data, even though the one dimension cannot be reduced, obviously. Our step size is 0.06, so 100 steps are equivalent to 6 ns. We ran k-means clustering[48] of the features, selecting 300 clusters. Running TICA and k-means clustering should not be necessary for an MSM with one feature, but we ran these anyways for continuity. From our implied timescale plots[49,50] we selected an MSM lag time of 500 steps (Figure S22). We then created Bayesian Markov state models, selecting two macrostates for each MSM. Using these parameters, we created MSMs and validated our two-state systems through CK tests[51–54] at a confidence interval of 95% (Figure S23). The estimated and predicted lines overlapped out to 1250 steps (>2.5x the MSM lag time number), which meant these MSMs are statistically valid.[51] We then used PCCA++ to cluster the first two eigenvectors of the MSM transition matrices to cluster the states.[55] The first macrostate in the H1N1-Shan2009 strain MSM (open) had a stationary distribution of 24.6% of the total population, while the second macrostate (closed) had a distribution of 75.4%. The first macrostate in the H1N1-Mich2015 strain MSM (open) had a stationary distribution of 26.7% of the total population, while the second macrostate (closed) had a distribution of 73.3%. Finally, we extracted HA structures from the two macrostates per strain by creating a 99.99999% probability cutoff for the H1N1-Shan2009 strain macrostates and a 99.9999% probability cutoff for the H1N1-Mich2015 strain macrostates. This means that the HA structures (microstates) that we selected have a >99.9999% probability of residing in the macrostate state that we think they are in through the PCCA++ fuzzy spectral clustering method. Confirmation of which microstates corresponded to which conformations was performed through the MDTraj software program.[56]

Figures and movies of the whole-virion simulations

Figure panels and movies of the whole-virion simulations were rendered from the trajectories using VMD GPU-accelerated ray tracing.[27,57]

1.2 Experimental Materials and Methods

Single-cell sorting, immunoglobulin amplification, sequencing and mAb production

Cryopreserved PBMC samples were thawed and stained with monoclonal antibodies against human CD3, CD14, CD56, CD19, CD21, CD27, CD38, IgM and IgG (BioLegends, BD BioSciences, Beckam Coulter). The NA probe N2 (A/Wisconsin/67/2005) was conjugated with either AX488 or AX647 fluorochrome according to the manufacturer's protocol (Microscale Protein Labeling Kit, Thermo Fisher Scientific) prior to use in flow cytometry. Non-B cells and dead cells were excluded with CD3, CD14, CD56, and aqua (live-dead) staining in the same fluorescent channel. Memory B cells were further gated on CD38low, CD27high, IgM- and IgG+ and NA-binding memory B cells were single-cell sorted into a 96-well plate (Bio-Rad) using an FACS Aria Instrument (BD Immunocytometry Systems). Plates were subjected to reverse transcription PCR and cDNA were used for PCR to amplify immunoglobulin (Ig) heavy and light chain genes as described previously.[58,59] PCR products were sequenced by Sangar sequencing. Ig heavy and light chain sequences were synthesized and cloned into IgG1 heavy and kappa backbone expression vector (GenScript). Antibody NDS.1 was expressed recombinantly by transient transfection of Ig expression plasmids in Expi293 cells with ExpiFectamine (Thermo Fisher Scientific). Supernatant was harvested 5 days post transfection and antibody was purified by using Protein A Sepharose (Thermo Fisher Scientific).

Biolayer interferometry (BLI)

BLI experiments were performed by using the Octet HTX instrument (Sartorius). HIS1K biosensors (Sartorius) were hydrated in PBS prior to use. Recombinant N2 NA tetramers derived from A/Wisconsin/67/2005 and A/Darwin/9/2021 were immobilized on HIS1K biosensors through their hexahistidine tags. After brief equilibration in assay buffer (25 mM Tris pH 8.0, 150 mM NaCl, 1% BSA), the biosensors were dipped into a two-fold dilution series of NDS.1 Fab for 5 min. Starting concentration of NDS.1 was 800 nM. Biosensors were then dipped in the assay buffer to allow Fab to dissociate from NA for 10 min. All assay steps were performed at 30°C with agitation set at 1,000 rpm. Baseline correction was carried out by subtracting the measurements recorded for a sensor loaded with the NA in the same buffer with no Fab. Data analysis and curve fitting were done with the Octet analysis software (version 11). Experimental data were fitted with the binding equations describing a 1:1 (Langmuir model) interaction.

Negative-stain electron microscopy

Samples were diluted to 0.02 mg/ml with buffer containing 10 mM HEPES, pH 7.0, and 150 mM NaCl, adsorbed to glow-discharged carbon-coated copper grids for 10 s, washed with three drops of the same buffer and negatively stained with 0.75% uranyl formate. Micrographs were recorded at a nominal magnification of 100,000 (corresponding to a pixel size of 2.2 Å) on an FEI Tecnai T20 electron microscope equipped with a 2k x 2k Eagle CCD camera and operated at 200 kV. Data collection was performed with SerialEM.[60] Particles were selected from micrographs using in-house written software (YT, unpublished) and extracted into 144 x 144-pixel boxes. After

2D classification, initial models were obtained and refined using Relion 3.1[61] with C4 symmetry enforced. The final datasets contained 2,304 and 4,368 particles for NDS.1 Fab and NDS.1/1G01 Fabs, respectively. The corresponding resolutions determined using the Fourier shell correlation threshold of 0.5 was 24.1 and 24 Å. UCSF Chimera[62] was used for docking and map visualization.

2. Supplementary Figures

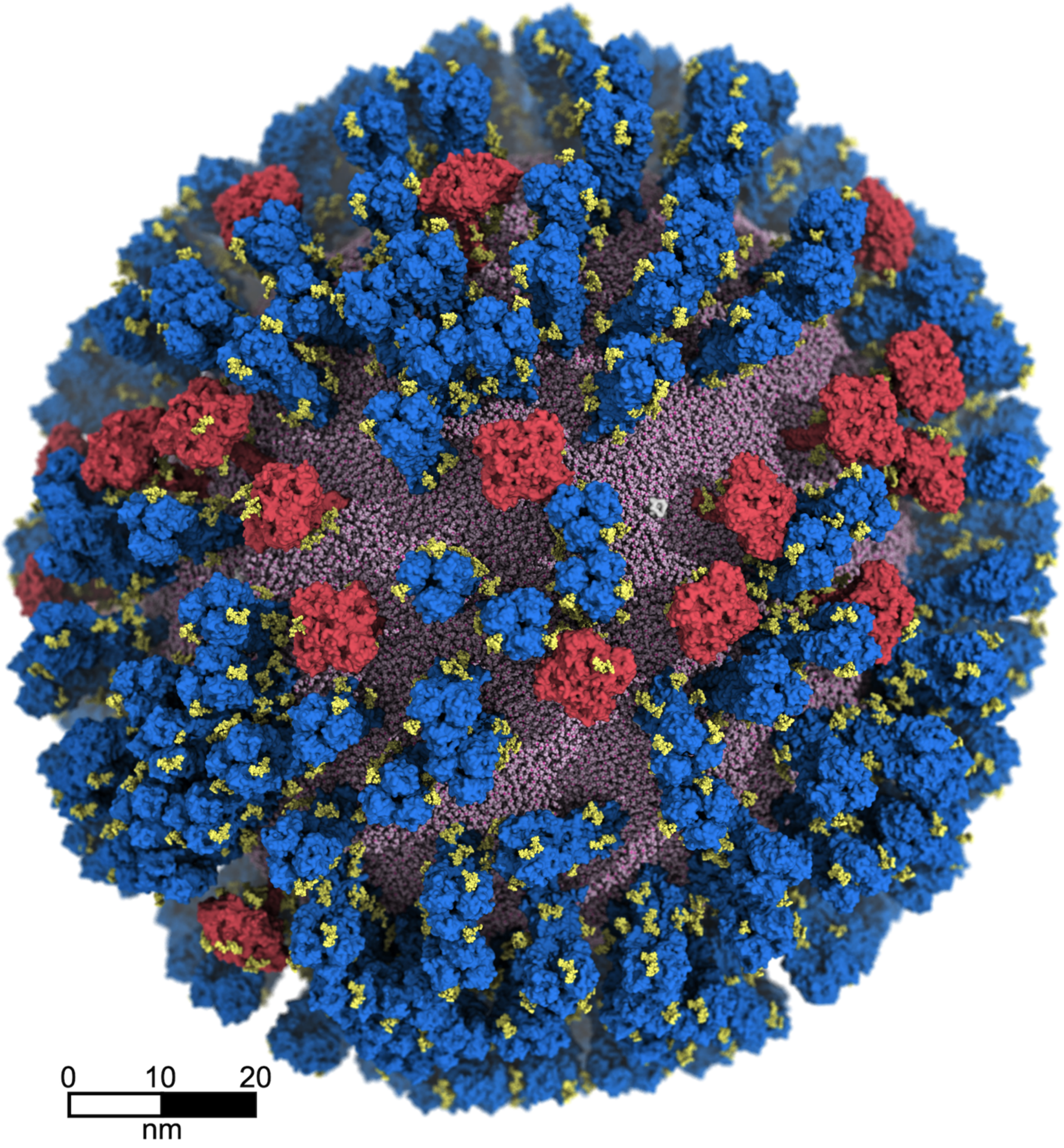

Figure S1. All-atom glycosylated model of H1N1-Mich2015 influenza A virion. Neuraminidase (NA) and hemagglutinin (HA) glycoproteins, and the M2 ion channels, are depicted with red, blue, and white surfaces, respectively. N-linked glycans are shown in yellow. The spherical lipid envelope is represented with pink van der Waals (vdW) spheres.

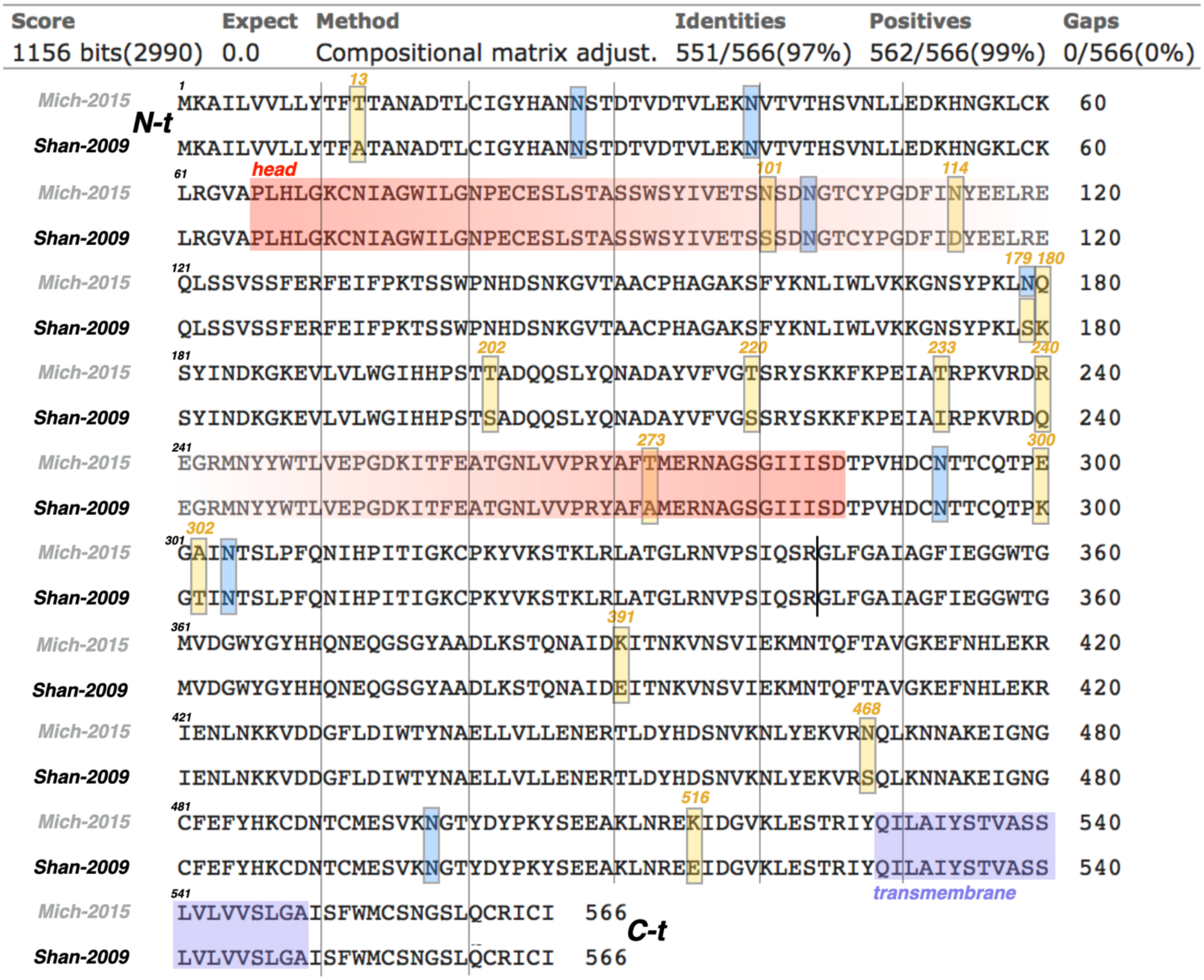

Figure S2. Aligned sequences of HA in H1N1-Shan2009 (UniProtKB accession code: F2YI86) and H1N1-Mich2015 (UniProtKB accession code: A0A144YDV8) as modeled in this work. N-linked glycosylation sites are highlighted with blue squares, whereas mutation sites with yellow squares. Alignment was performed with BLAST.[63]

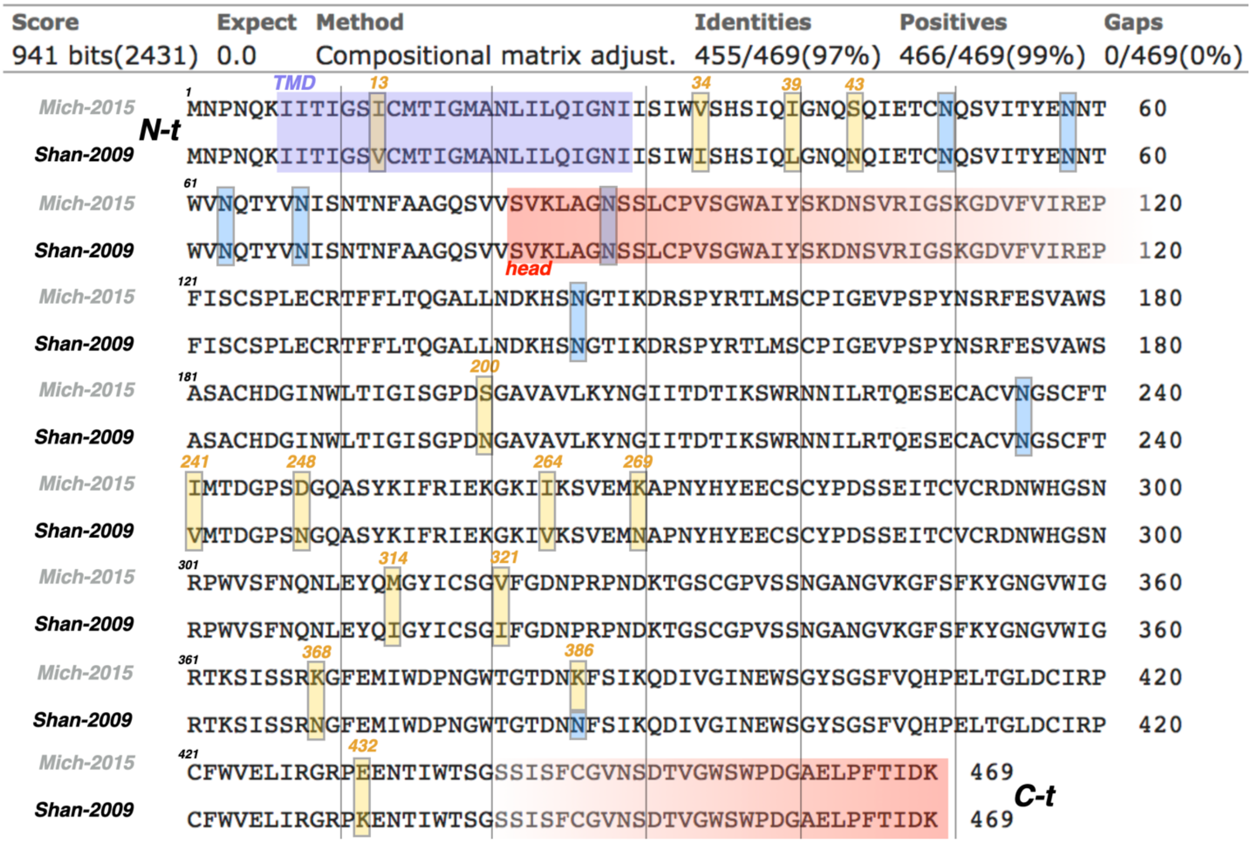

Figure S3. Aligned sequences of NA in H1N1-Shan2009 (UniProtKB accession code: F2YI87) and H1N1-Mich2015 (UniProtKB accession code: A0A0X9QTS2) as modeled in this work. N-linked glycosylation sites are highlighted with blue squares, whereas mutation sites with yellow squares. Alignment was performed with BLAST.[63]

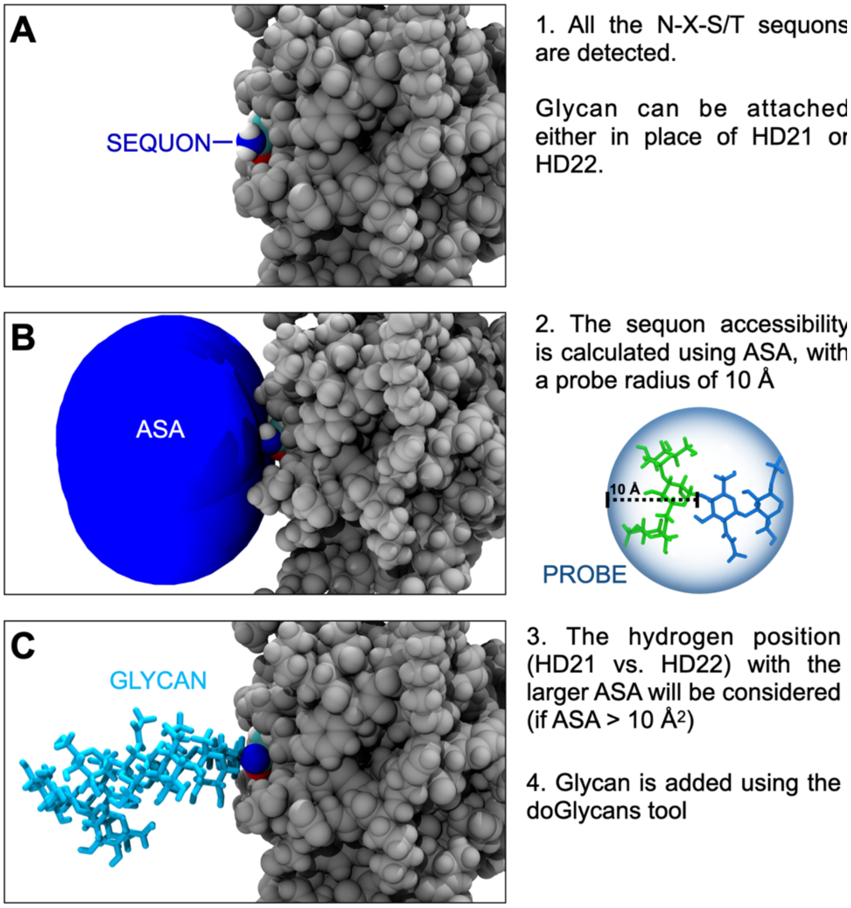

Figure S4. Schematic representation of the glycosylation procedure. **A**) N-X-S/T N-linked sequons are selected. **B**) Although all sequons are overall highly occupied as reported by previous studies,[22,30,31] in this work we evaluated the accessibility of each selected sequon by calculating the accessible surface area (ASA) of the asparagine side chain's ND2 atom using a probe radius of 10 Å. This was done since the initial coordinates of the whole-virion construct are derived from previous MD simulation of the unglycosylated whole-virion construct.[1] **C**) Addition of glycan.

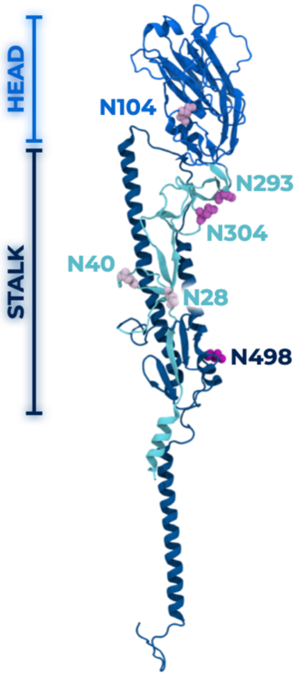

HA

Sequon	Region	Structure	#	%
N28	Stalk	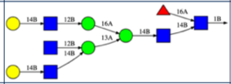	428/708	60.5%
N40	Stalk	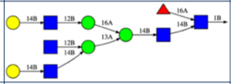	299/708	42.2%
N104	Head	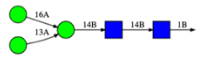	29/708	4.1%
N293	Stalk	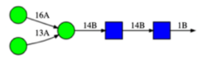	36/708	5.1%
N304	Stalk	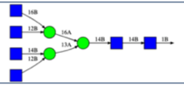	95/708	13.4%
N498	Stalk	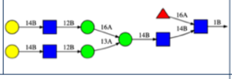	390/708	55.1%
TOTAL			1277/4248	30%

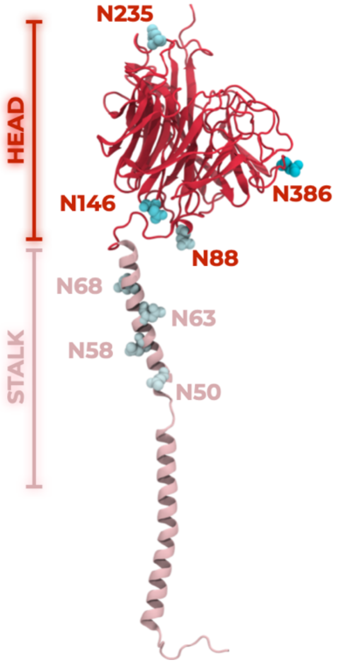

NA

Sequon	Region	Structure	#	%
N50	Stalk	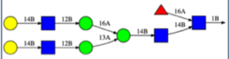	30/120	25.0%
N58	Stalk	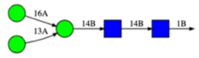	0/120	0%
N63	Stalk	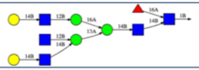	38/120	31.7%
N68	Stalk	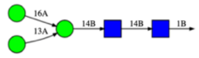	10/120	8.3%
N88	Head	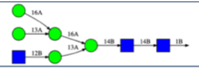	37/120	30.8%
N146	Head	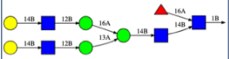	16/120	13.3%
N235	Head	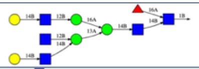	32/120	26.7%
N386	Head	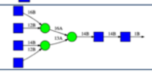	81/120	67.5%
TOTAL			244/960	25.4%

Figure S5. Glycosylation of the 708 HA monomers (top) and 120 NA monomers (bottom) included in the H1N1-Shan2009 whole-virion model. The position of N-linked glycosylation sites on HA (top) and NA (bottom) monomeric structure is shown on the left panels. The structure, total number, and relative frequency (%) of glycans added at each N-linked site are summarized on the right panels.

HA

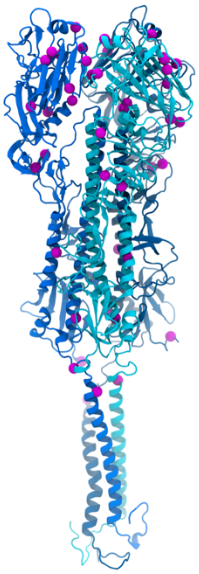

#	RES NUMBER	H1N1-Mich2015 MUTATION
1	13	A13T
2	101	S101N
3	114	D114N
4	179	S179N
5	180	K180Q
6	202	S202T
7	220	S220T
8	233	I233T
9	240	Q240R
10	273	A273T
11	300	K300E
12	302	T302A
13	391	E391K
14	468	S468N
15	516	E516K

NA

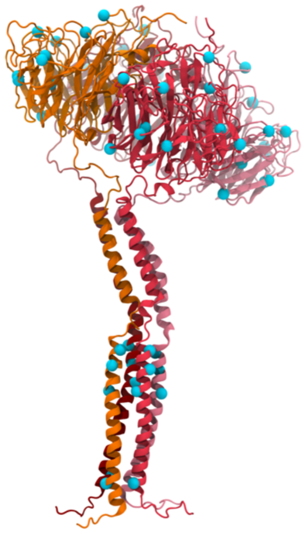

#	RES NUMBER	H1N1-Mich2015 MUTATION
1	13	V13I
2	34	I34V
3	39	L39I
4	43	N43S
5	200	N200S
6	241	V241I
7	248	N248D
8	264	V264I
9	269	N269K
10	314	I314M
11	321	I321V
12	368	N368K
13	386	N386K
14	432	K432E

Figure S6. List of amino acid point mutations in H1N1-Mich2015 with respect to H1N-Shan2009 for HA (left panel) and NA (right panel). The addition of N-linked sequon within HA head at position N179 is highlighted in blue. Deletion of N-linked sequon within NA head at position N386 highlighted in red. The location of the point mutations within HA (left) and NA (right) structures is highlighted with magenta and cyan beads, respectively.

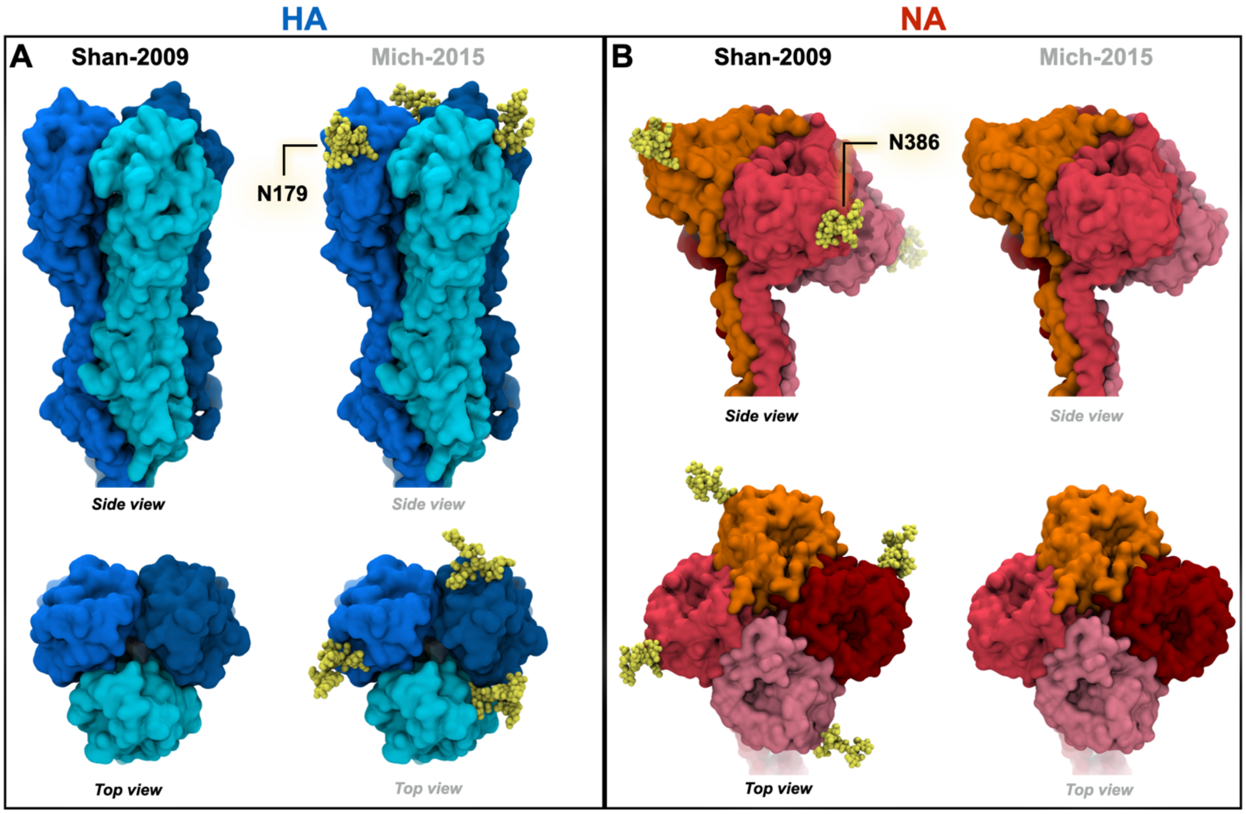

Figure S7. Glycan variation in H1N1-Mich2015 with respect to H1N1-Shan2009 for HA (**A**) and NA (**B**). A new N-linked glycan is introduced at position N179 within the HA head (**A**). N-linked glycan at position N386 within the NA head is instead deleted (**B**). The glycan is illustrated with yellow van der Waals spheres, whereas HA and NA are represented with cyan and red surfaces, respectively.

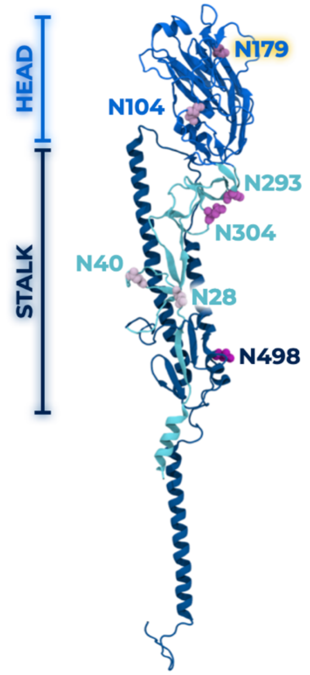

HA

Sequon	Region	Structure	#	%
N28	Stalk	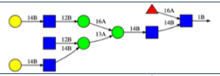	420/708	59.3
N40	Stalk	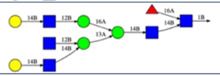	288/708	40.7%
N104	Head	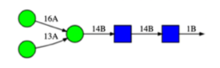	33/708	4.7%
N179	Head	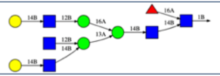	351/708	49.6%
N293	Stalk	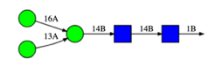	39/708	5.5%
N304	Stalk	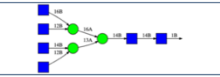	102/708	14.4%
N498	Stalk	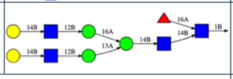	393/708	55.5%
TOTAL			1626/4956	32.8%

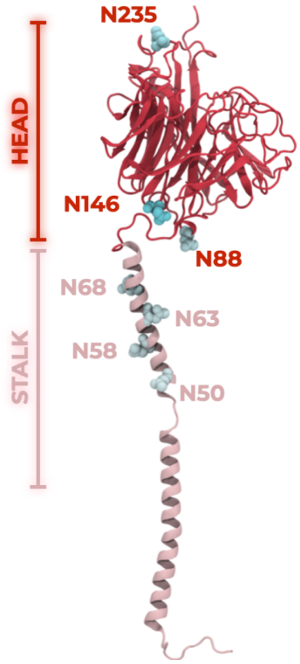

NA

Sequon	Region	Structure	#	%
N50	Stalk	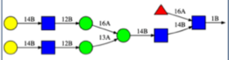	31/120	25.8%
N58	Stalk	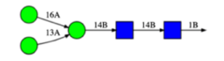	0/120	0%
N63	Stalk	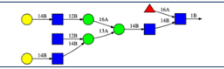	40/120	33.3%
N68	Stalk	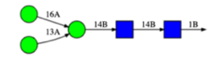	11/120	9.2%
N88	Head	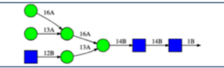	36/120	30.0%
N146	Head	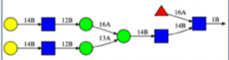	18/120	15.0%
N235	Head	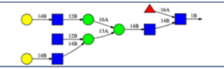	29/120	24.2%
~~N386~~	–	–	–	–
TOTAL			165/840	19.6%

Figure S8. Glycosylation of the 708 HA monomers (top) and 120 NA monomers (bottom) included in the H1N1-Mich2015 whole-virion model. The position of N-linked glycosylation sites on the HA (top) and NA (bottom) monomeric structure is shown on the left panels. The structure, total number, and relative frequency (%) of glycans added at each N-linked site are summarized on the right panels.

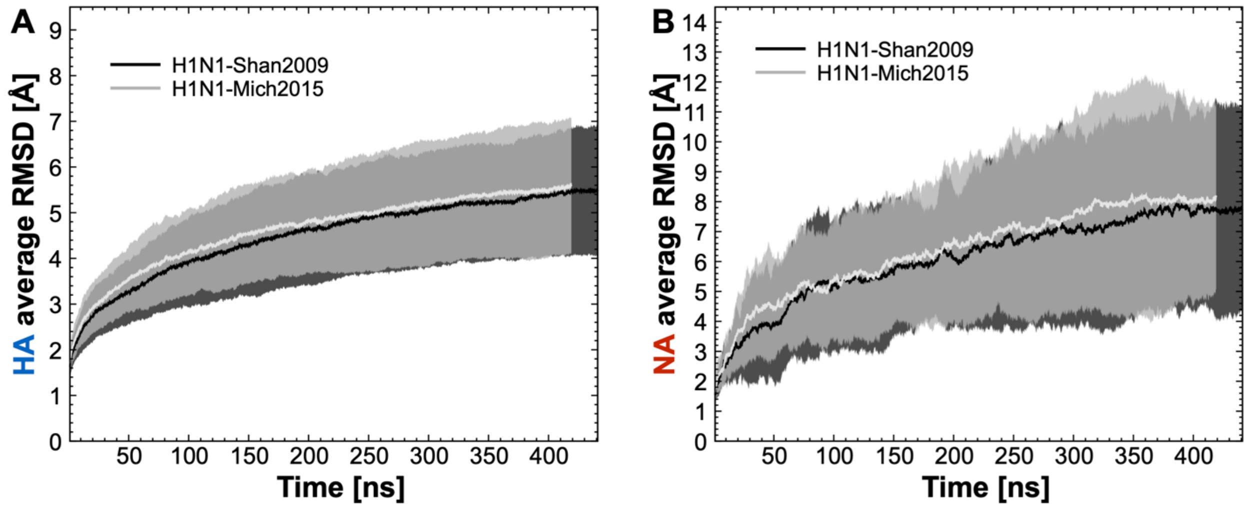

Figure S9. Average RMSD profiles for HA (**A**) and NA (**B**) as calculated over the course of H1N1-Shan2009 (black line) and H1N1-Mich2015 (gray line) whole-virion MD simulations. RMSD values have been averaged across the 236 HA trimers and 30 NA tetramers, respectively. Standard deviation values are plotted with a semi-transparent filled area.

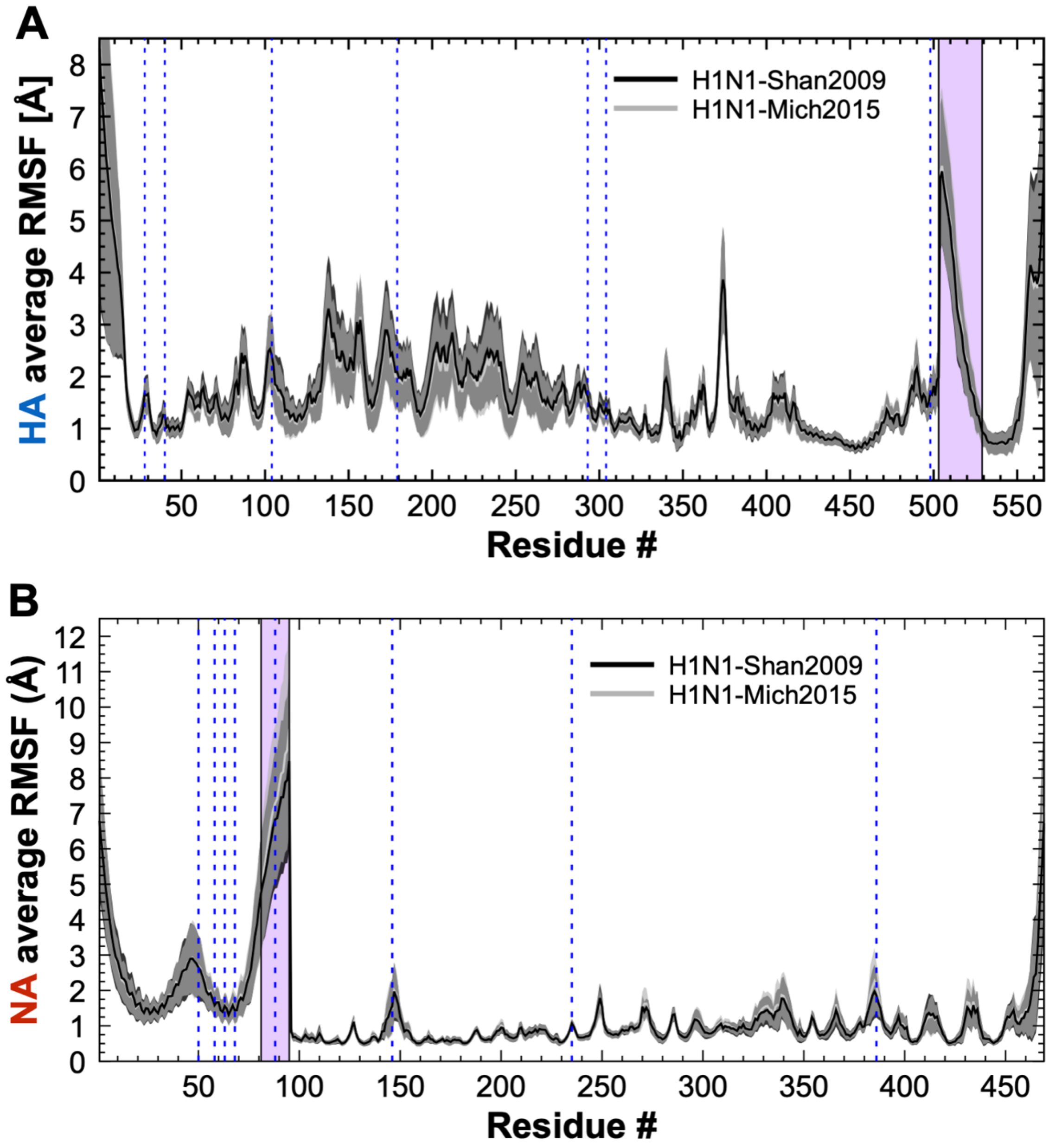

Figure S10. Average RMSF profiles for HA (**A**) and NA (**B**) as calculated over the course of H1N1-Shan2009 (black line) and H1N1-Mich2015 (gray line) whole-virion MD simulations. RMSF values have been averaged across the 708 HA and 120 NA monomers, respectively. Standard deviation values are plotted with a semi-transparent filled area. Highlighted with a transparent purple rectangle is the flexible hinge region for HA and NA facilitating HA ectodomain tilting and NA head tilting, respectively. The left side of the purple rectangle in the HA RMSF profile demarcates the two alignment HA regions used for RMSF calculation. The left side of the purple rectangle in the HA RMSF profile demarcates the two alignment regions used for RMSF calculation.

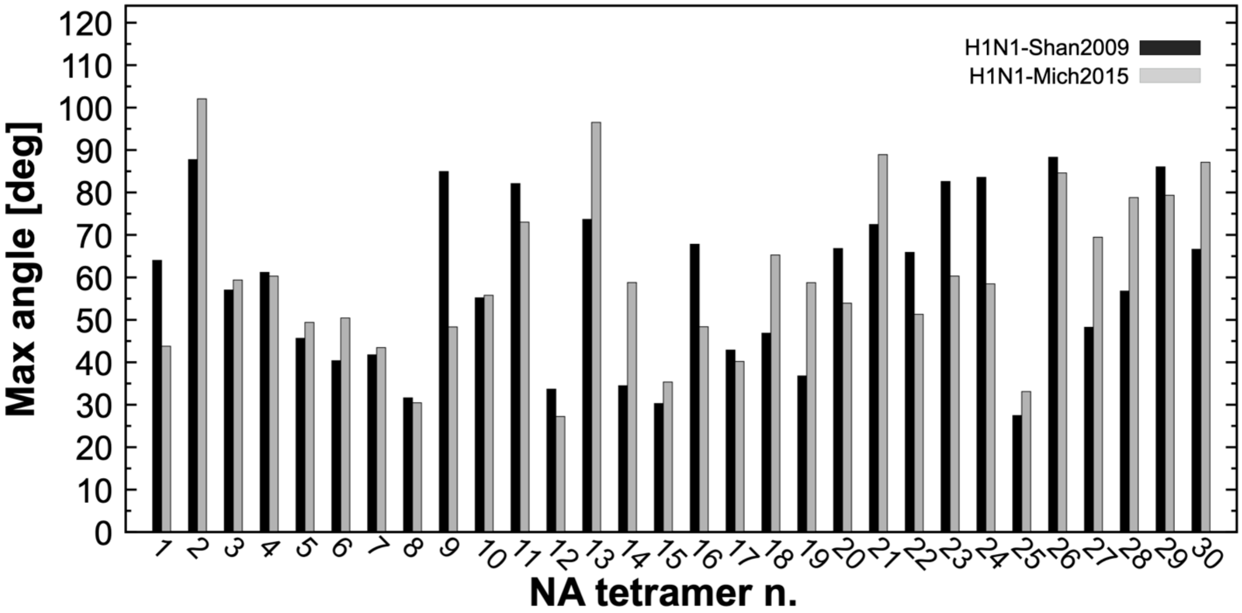

Figure S11. Maximum NA head-tilt angle observed in H1N1-Shan2009 and H1N1-Mich2015 NA tetramers. For each NA tetramer (numbered from #1 to #30), the maximum NA head-tilt angle values (degree) calculated during the simulations of H1N1-Shan2009 and H1N1-Mich205 are shown with black and gray bars, respectively.

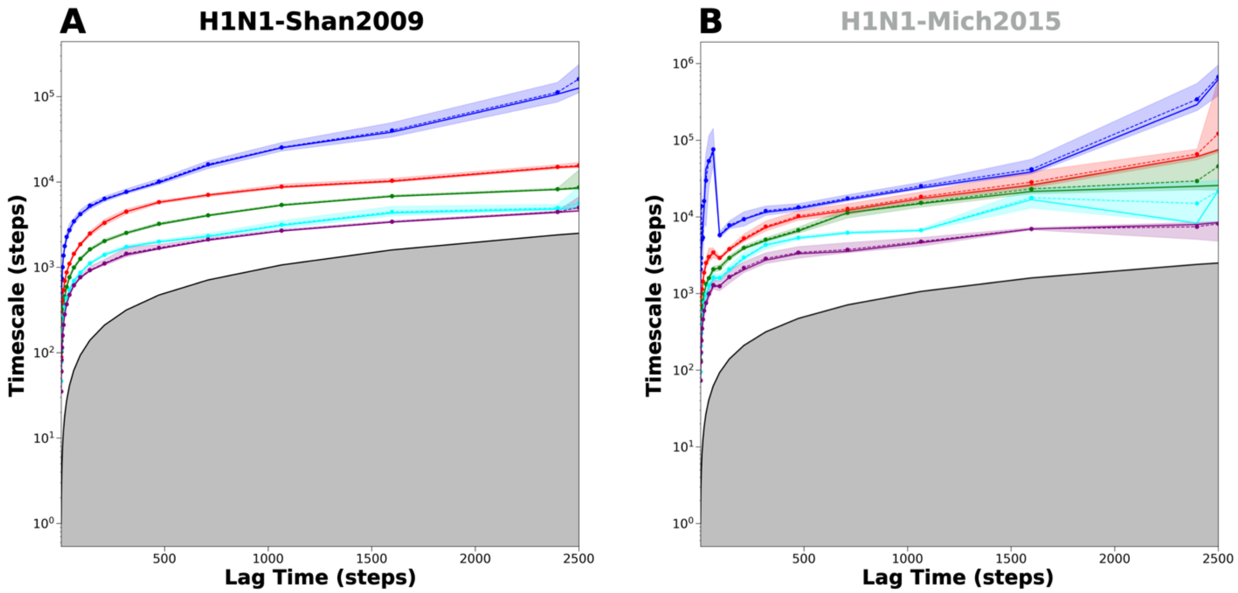

Figure S12. The implied timescale plots for the MSM of the NA head tilting in (**A**) H1N1-Shan2009 and (**B**) H1N1-Mich2015. The shading on each of the implied timescales represents statistical uncertainties calculated through Bayesian sampling. From these figures, the lag time was selected to be 250 steps and two macrostates were selected. The gray region represents forbidden space, which is faster than the lag time; transitions in this region cannot be resolved.

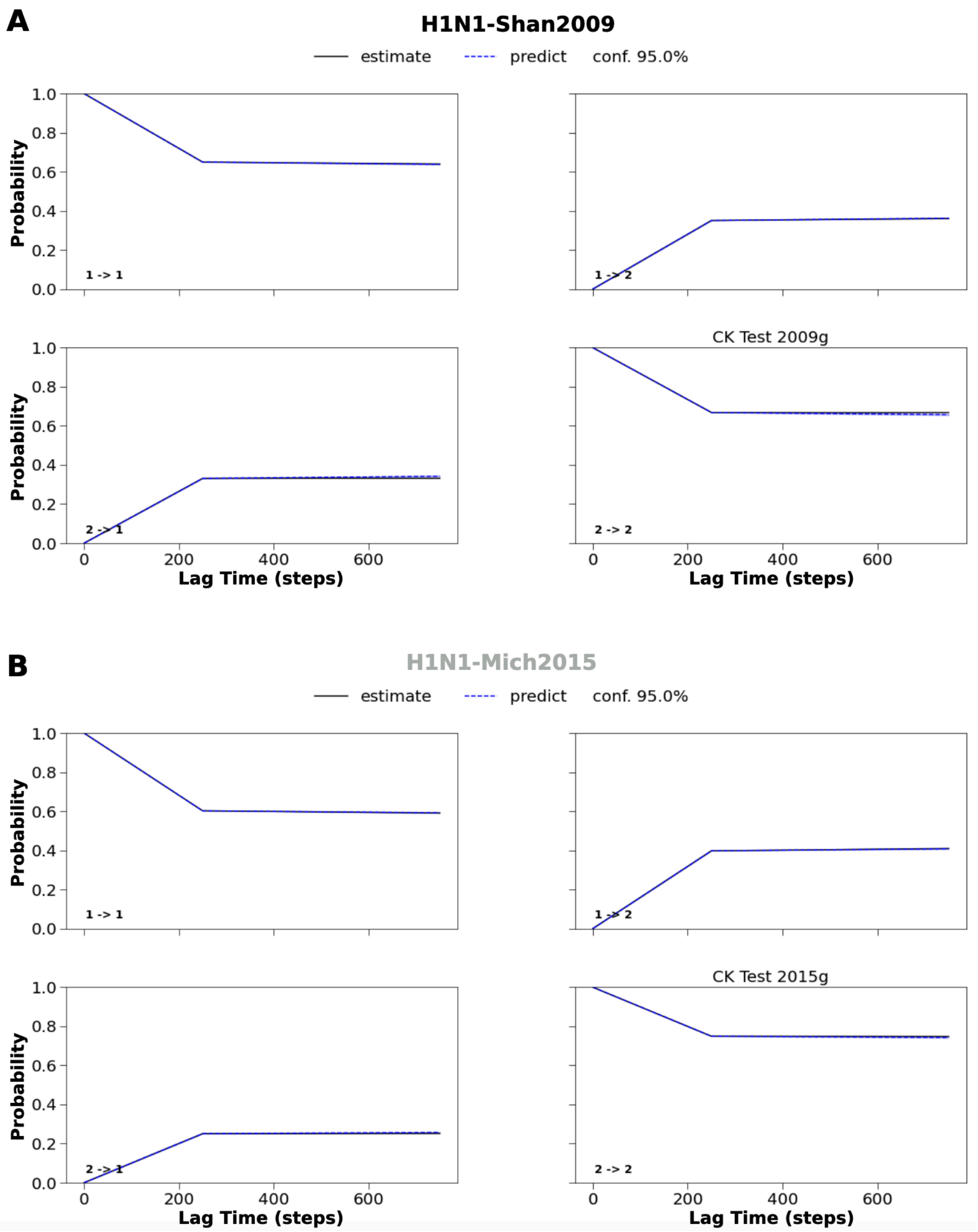

Figure S13. The CK test plots for the MSM of the NA head tilting in (**A**) H1N1-Shan2009 and (**B**) H1N1-Mich2015. The lag time was selected to be 250 steps, with two macrostates selected. The solid line (the "estimate") represents the transition probabilities of the model created at a lag time of 250 steps, with these probabilities then estimated at multiples of the lag time. The dotted line (the "prediction") represents predicted probabilities of the same model independently created at each of these multiples of the lag time. Dark blue shading represents a 95% confidence interval for the predicted probabilities. Since the estimates of the MSM model that we used overlap with the predicted probabilities of the model at 3x the lag time, this model satisfies Markovianity, passes the CK test and can be used to extract transition kinetics. The numbers in the lower left corners represent macrostate transitions; thus "1 -> 1" represents a transition from macrostate 1 to macrostate 1.

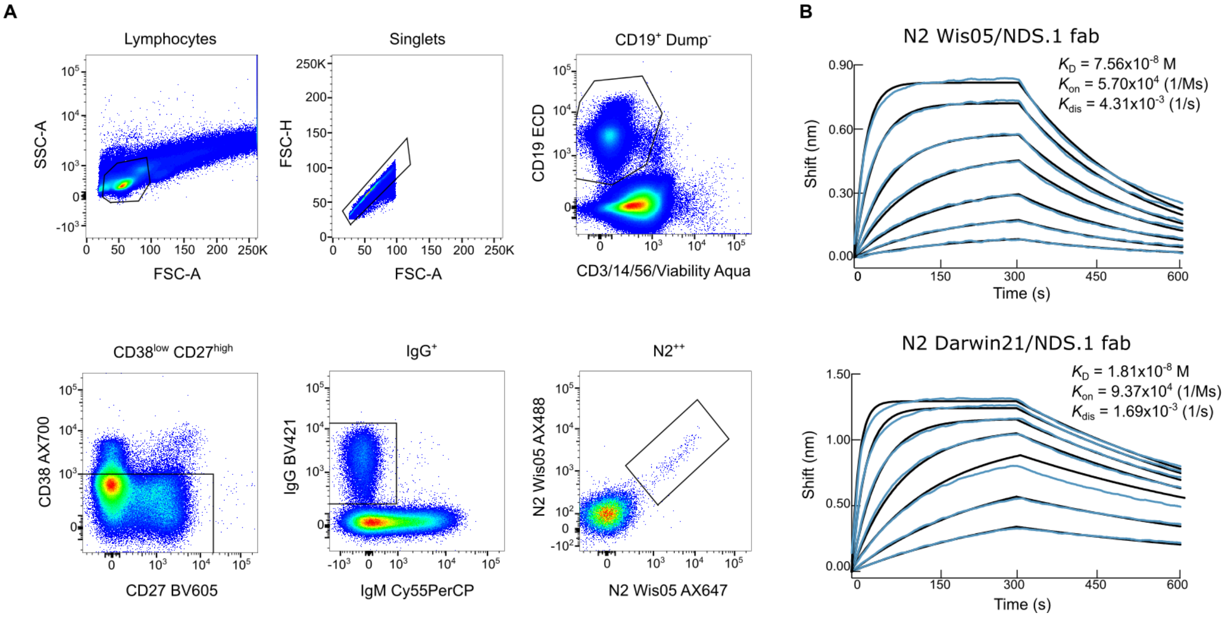

Figure S14. Isolation and characterization of NA-directed mAb NDS.1. (**A**) Gating strategy of NA-specific memory B-cells by flow cytometry. (**B**) Binding kinetics of NDS.1 Fab to N2-Wis05 and N2-Darwin21 by biolayer interferometry. Experimental data (blue traces) were fitted (black lines) with the binding equations describing a 1:1 interaction.

Darwin21 N2/NDS.1 Fab

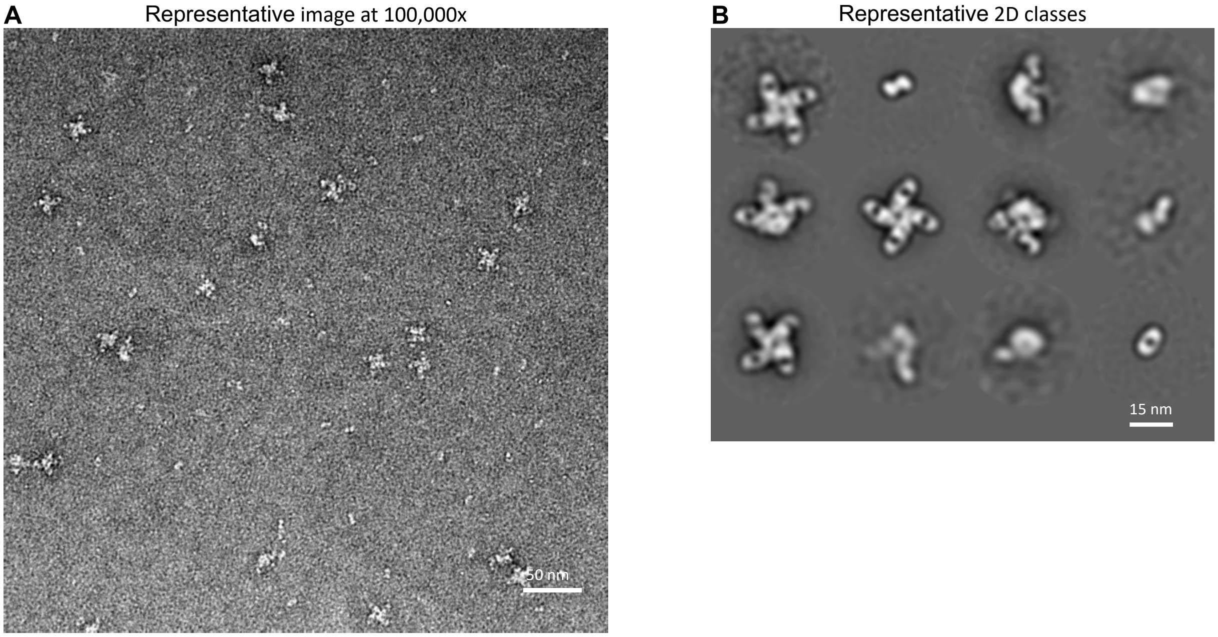

Figure S15. Negative-stain EM analysis of N2 A/Darwin/9/2021 NA tetramer in complex with Fab NDS.1 (**A)** Representative raw NS-EM image and (**B**) 2D class averages of N2 A/Darwin/9/2021 NA in complex with Fab NDS.1 (scale bar: 50 nm and 15 nm).

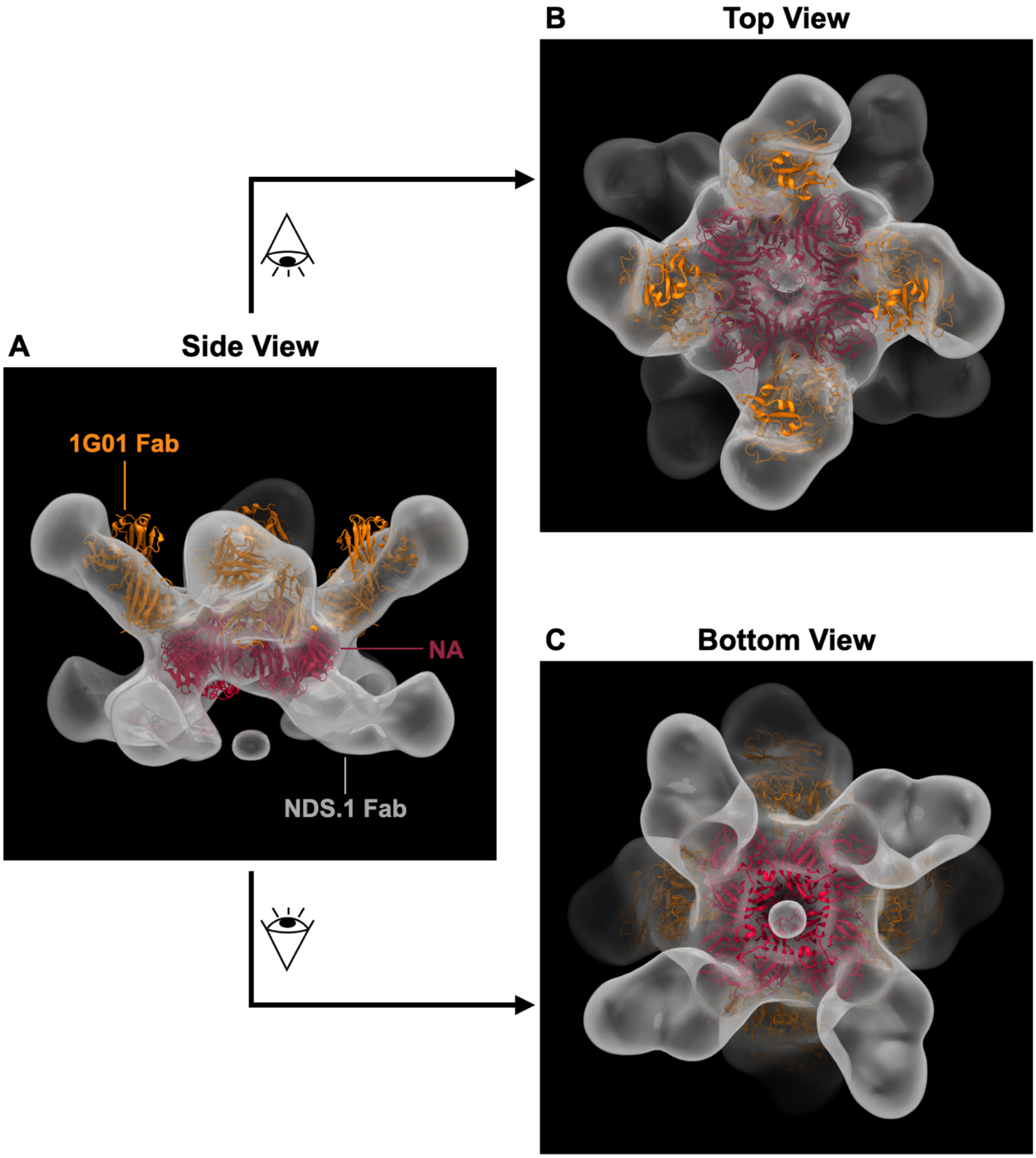

Figure S16. NS-EM reconstruction model of N2 A/Indiana/10/2011 NA tetramer in complex with NDS.1 Fab and 1G01 Fab. Side view (**A**), top view (**B**) and bottom view (**C**) of the ternary complex. Crystal structure of NA-1G01 complex (PDB ID: 6Q23[64]) is fitted into the NS-EM density map. The final model was generated using a dataset containing 4,368 particles.

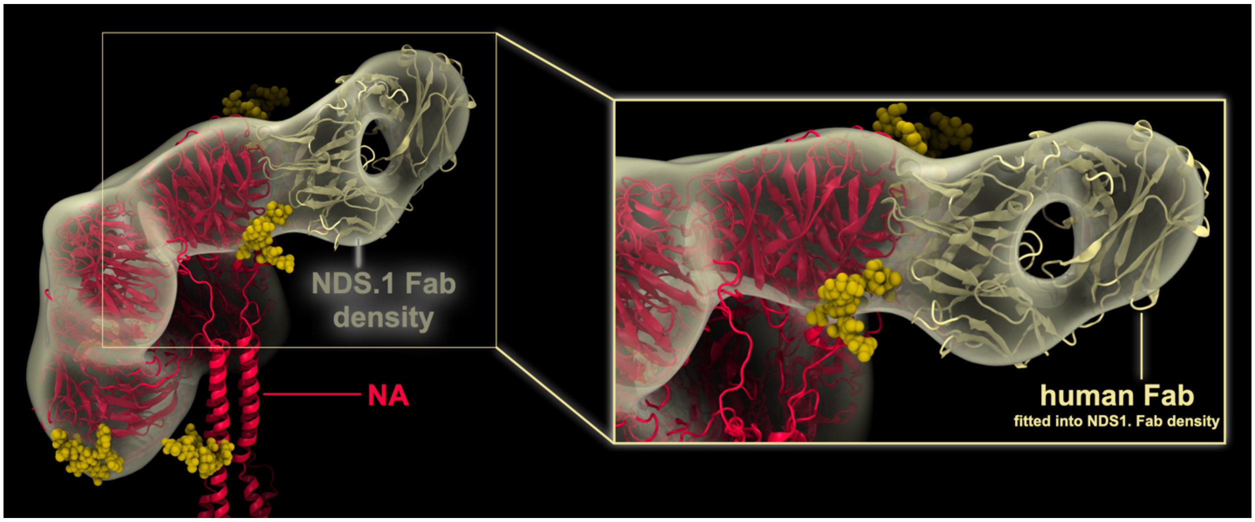

Figure S17. The NA head-underside epitope. A mock Fab, namely the crystal structure of the human immunoglobulin fragment Fab New (IgG1,λ) (PDB ID: 7FAB[65]), together with one conformation of the NA with a titled head extracted from the simulation of H1N1-Shan2009, was fitted into the NS-EM density map of the N2 A/Indiana/10/2011 NA tetramer in complex with NDS.1 Fab. NA is represented with red cartoons, whereas the human immunoglobulin fragment Fab is shown with yellow cartoons. The NS-EM density is depicted with a transparent yellow surface. Glycans are shown with yellow vdW spheres.

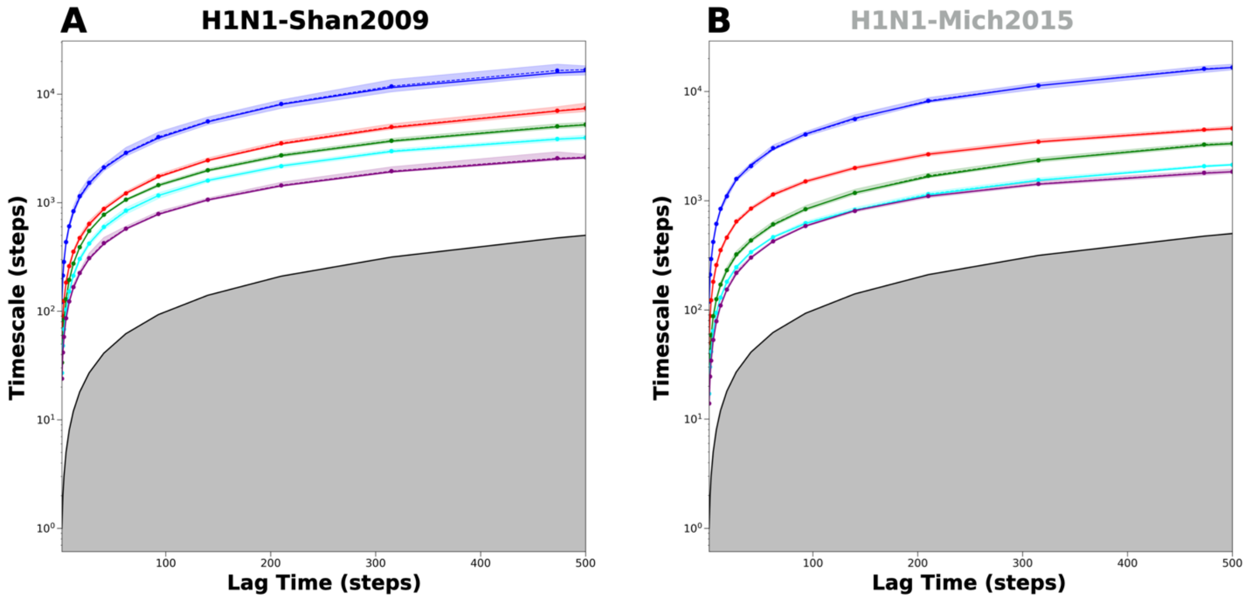

Figure S18. The implied timescale plots for the MSM of the HA ectodomain tilting in (**A**) H1N1-Shan2009 and (**B**) H1N1-Mich2015. The shading on each of the implied timescales represents statistical uncertainties calculated through Bayesian sampling. From these figures, the lag time was selected to be 50 steps and two macrostates were selected. The gray region represents forbidden space, which is faster than the lag time; transitions in this region cannot be resolved.

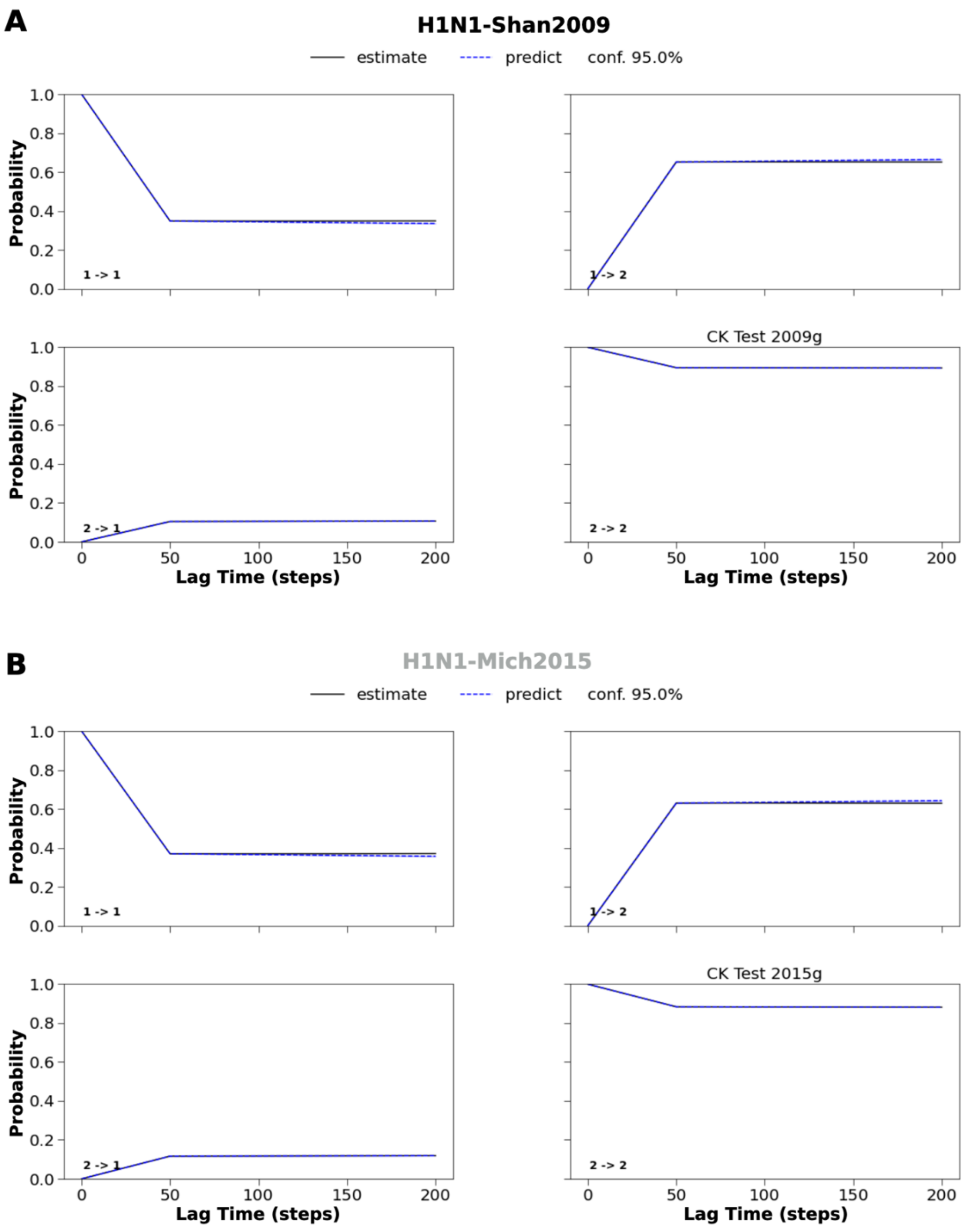

Figure S19. The CK test plots for the MSM of the HA ectodomain tilting in (**A**) H1N1-Shan2009 and (**B**) H1N1-Mich2015. The lag time was selected to be 50 steps, with two macrostates selected. The solid line (the "estimate") represents the transition probabilities of the model created at a lag time of 50 steps, with these probabilities then estimated at multiples of the lag time. The dotted line (the "prediction") represents predicted probabilities of the same model independently created at each of these multiples of the lag time. Dark blue shading represents a 95% confidence interval for the predicted probabilities. Since the estimates of the MSM model that we used overlap with the predicted probabilities of the model at 3x the lag time, this model satisfies Markovianity, passes the CK test and can be used to extract transition kinetics. The numbers in the lower left corners represent macrostate transitions; thus "1 -> 1" represents a transition from macrostate 1 to macrostate 1.

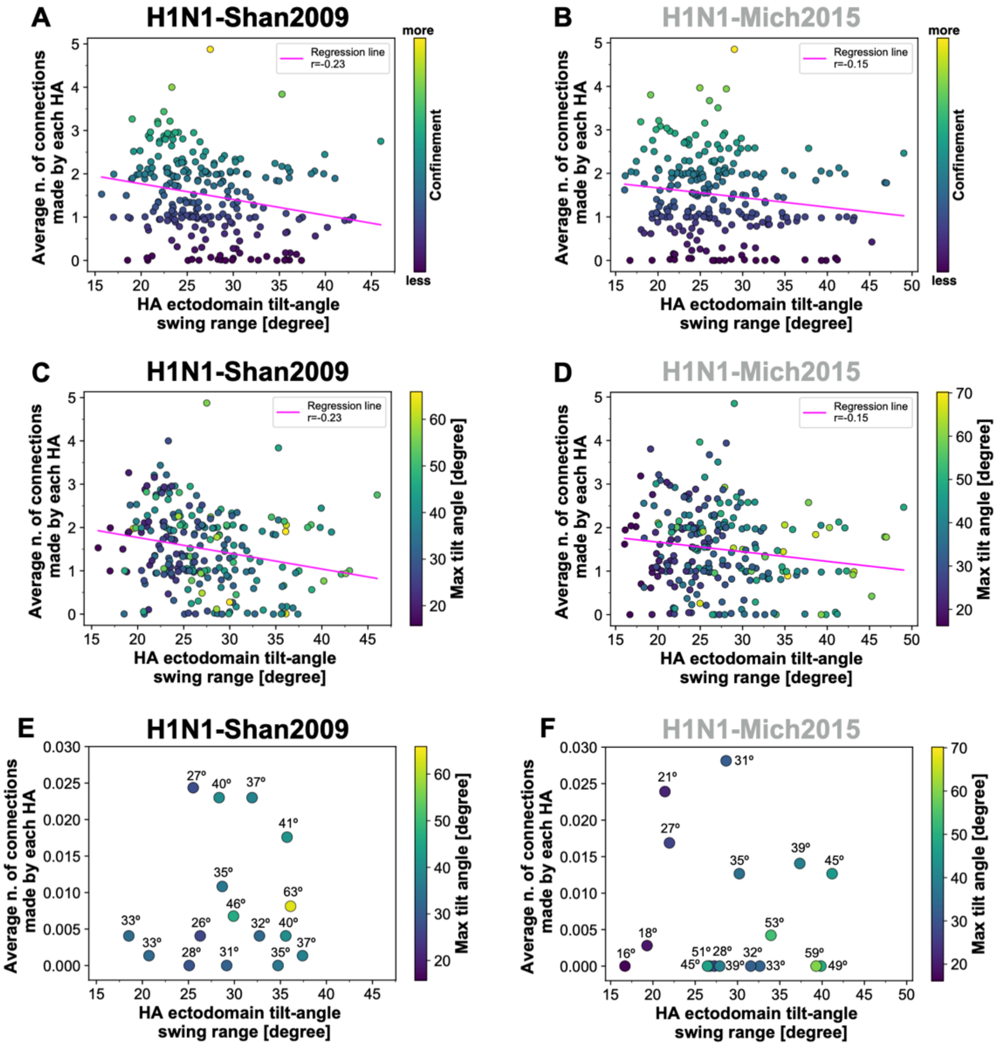

Figure S20. Analysis of the correlation between HA ectodomain tilting and HA protein confinement. The correlation between the HA ectodomain tilt-angle swing range (*x-axis*) and the average number of connections made by each HA (*y-axis*) is illustrated in the scatter plots (**A**) and (**B**) for H1N1-Shan2009 and H1N1-Mich2015, respectively. The tilt-angle swing range is calculated for each HA ectodomain as (*max_tilt-angle_value – min_tilt-angle_value*) exhibited during the respective dynamics. The tilt angle and the connections made by each HA with neighboring glycoproteins are evaluated as described in the Material and Methods section. The regression line is shown in magenta. Each point is colored according to the extent of protein confinement using the viridis palette: the higher the average number of connections made by each HA the more the respective confinement. The same plots are replicated in panels (**C**) and (**D**) where instead each point is colored according to the maximum value of tilting angle exhibited during the simulations by the respective HA ectodomain. Plots (**E**) and (**F**) are a subsection of plots (**C**) and (**D**), respectively, where only the 0.000–0.0030 range is considered on the y-axis. These plots show the extent of tilting for the HA glycoproteins that remain isolated during the simulations, i.e., the ones that do not interact with any neighboring glycoproteins. The value of the maximum tilt angle achieved during the dynamics is reported for each HA.

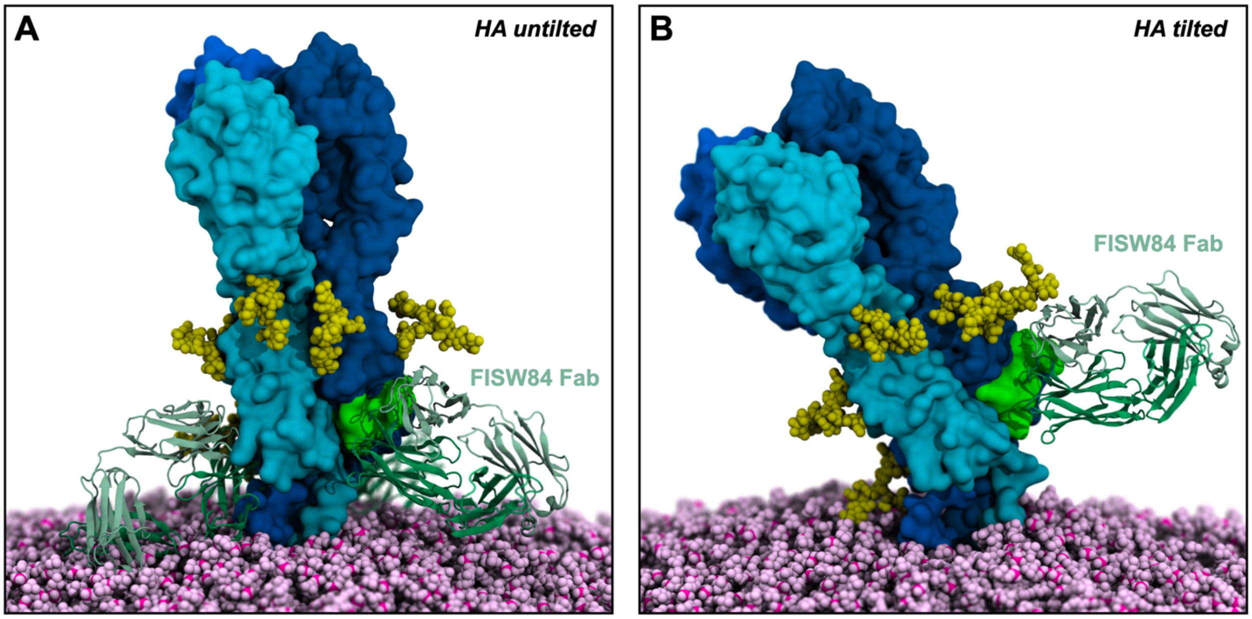

Figure S21. HA anchor epitope accessibility. **A**) When HA is untilted, the FISW84 Fab[66] must approach the anchor epitope with an upward angle. FISW84 is depicted with green cartoons, whereas the three HA monomers are depicted with surfaces colored with different shades of blue. The anchor epitope is highlighted with a green surface. N-glycans are shown with yellow vdW spheres, whereas the POPC lipids are displayed with pink vdW spheres. **B**) When HA flexes onto the membrane, the angle of approach to at least one epitope gradually shifts from upward to lateral or slightly downward, facilitating FISW84 Fab binding.

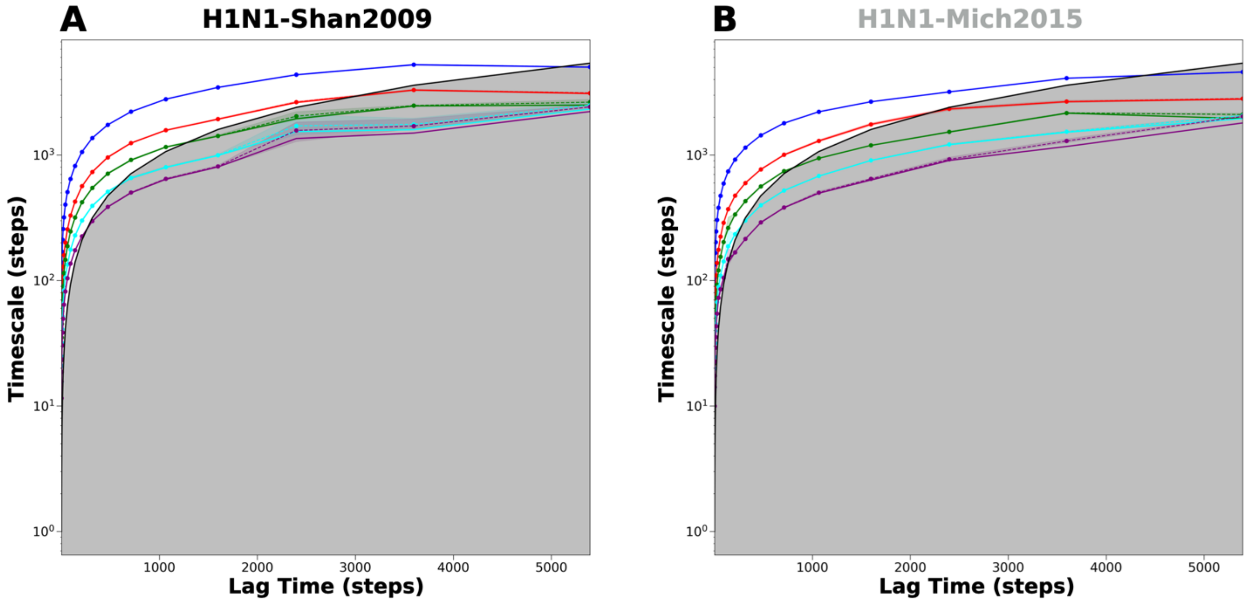

Figure S22. The implied timescale plots for the MSM of the HA head breathing in (**A**) H1N1-Shan2009 and (**B**) H1N1-Mich2015. The shading on each of the implied timescales represents statistical uncertainties calculated through Bayesian sampling. From these figures, the lag time was selected to be 500 steps and two macrostates were selected. The gray region represents forbidden space, which is faster than the lag time; transitions in this region cannot be resolved.

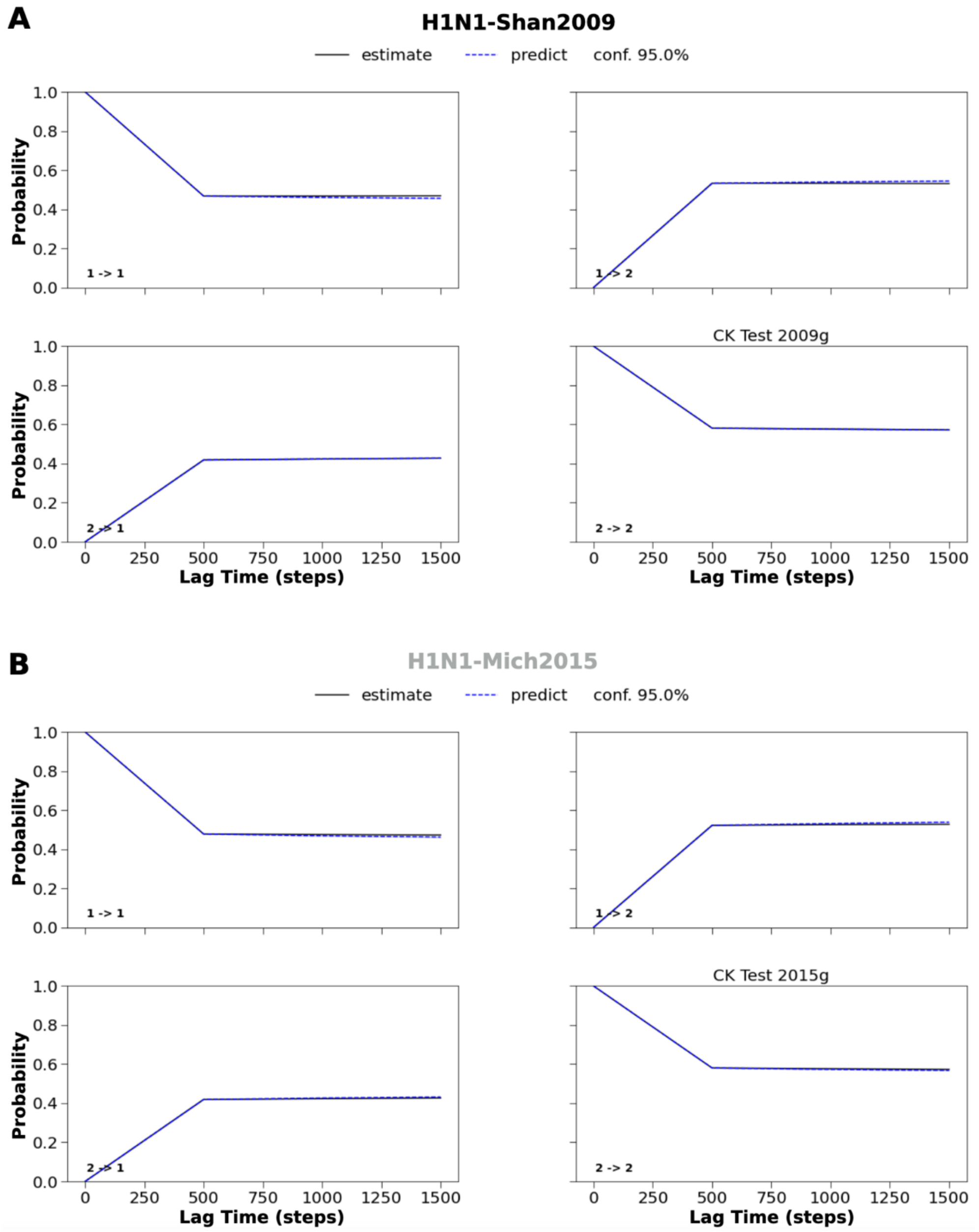

Figure S23. The CK test plots for the MSM of the HA head breathing in (**A**) H1N1-Shan2009 and (**B**) H1N1-Mich2015. The lag time was selected to be 500 steps, with two macrostates selected. The solid line (the "estimate") represents the transition probabilities of the model created at a lag time of 500 steps, with these probabilities then estimated at multiples of the lag time. The dotted line (the "prediction") represents predicted probabilities of the same model independently created at each of these multiples of the lag time. Dark blue shading represents a 95% confidence interval for the predicted probabilities. Since the estimates of the MSM model we used overlap with the predicted probabilities of the model at 3x the lag time, this model satisfies Markovianity, passes the CK test and can be used to extract transition kinetics. The numbers in the lower left corners represent macrostate transitions; thus "1 -> 1" represents a transition from macrostate 1 to macrostate 1.

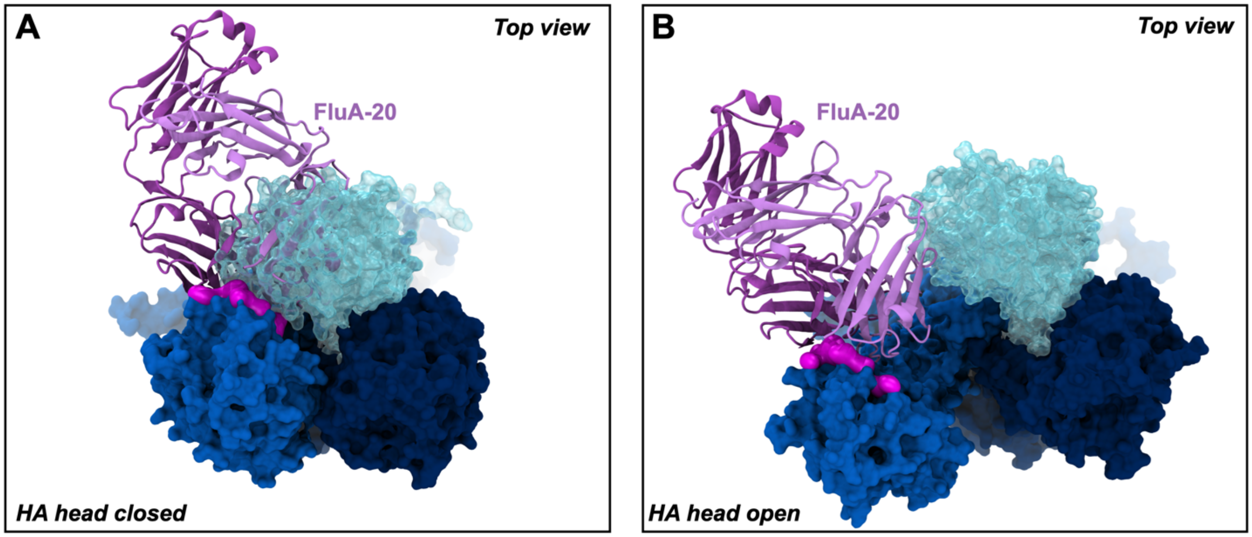

Figure S24. HA head trimer interface epitope accessibility. **A**) When HA heads are closed, the FluA-20 antibody[42] cannot access the epitope buried in the head trimer interface. FluA-20 is depicted with purple cartoons, whereas the three HA monomers are depicted with surfaces colored with different shades of blue. The FluA-20 epitope is highlighted with a magenta surface on one HA head. **B**) When one HA breathes, the FluA-20 epitope becomes accessible allowing antibody binding.

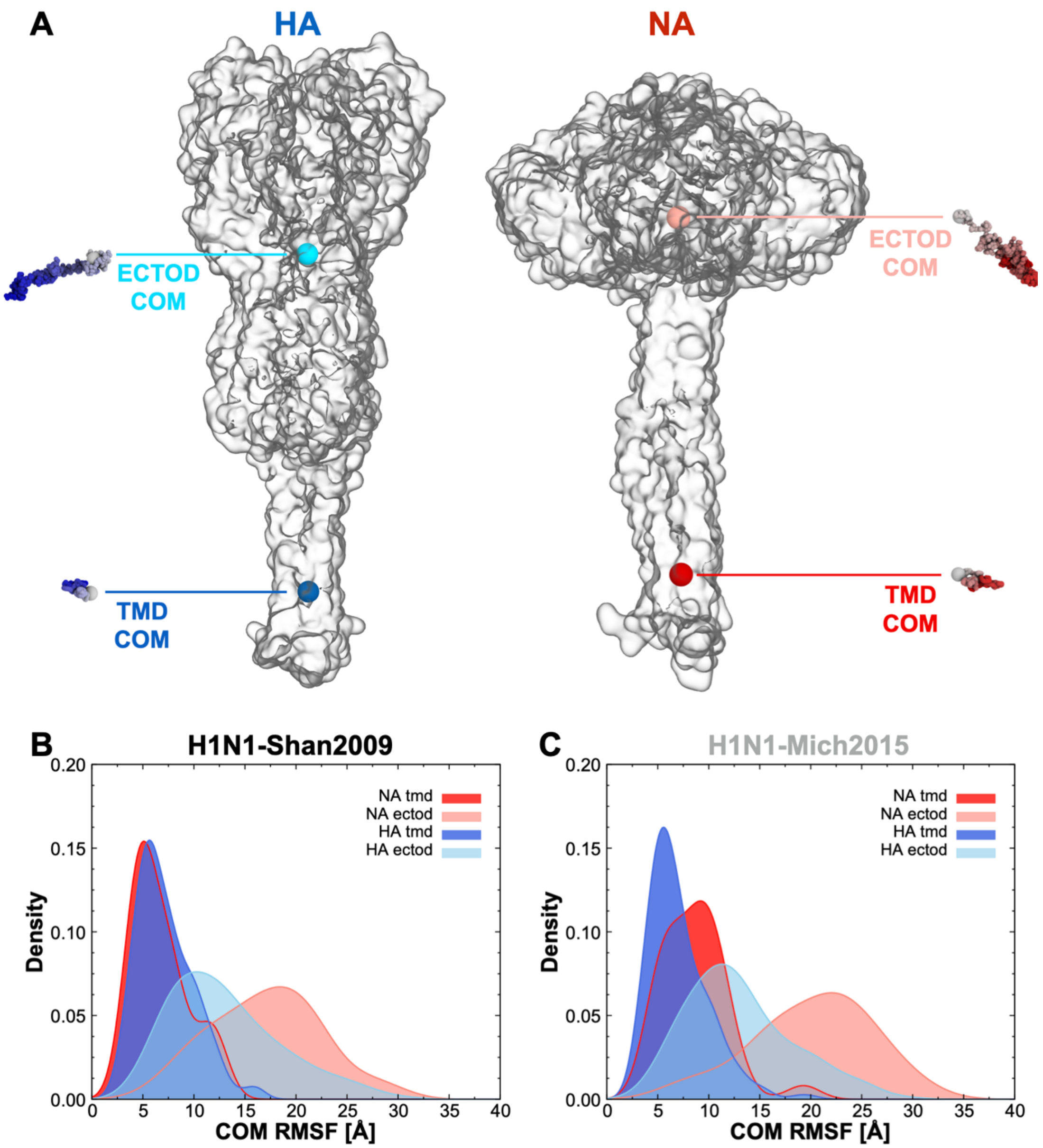

Figure S25. RMSF of HA and NA transmembrane domain (TMD) COM and ectodomain COM. The initial frame was used as a reference for RMSF calculation. **A**) Molecular representation of an HA trimer (left) and a NA tetramer (right) using a transparent surface. TMD COM and ectodomain COM of HA and NA are indicated with cyan and red spheres, respectively. As an example, the position of TMD COM and ectodomain COM is displayed with a sphere every 10th frame of the MD simulation for a representative HA trimer and a representative NA tetramer selected from H1N1-Shan2009. The spheres are colored according to simulation time using a white-to-blue gradient for HA and a white-to-red gradient for NA. **B**) Kernel density distribution profiles of RMSF values calculated for the HA (236 trimers) and NA (30 tetramers) TMD COMs and ectodomain COMs in H1N1-Shan2009. **C**) Kernel density distribution profiles of RMSF values calculated for the HA (236 trimers) and NA (30 tetramers) TMD COMs and ectodomain COMs in H1N1-Mich2015.

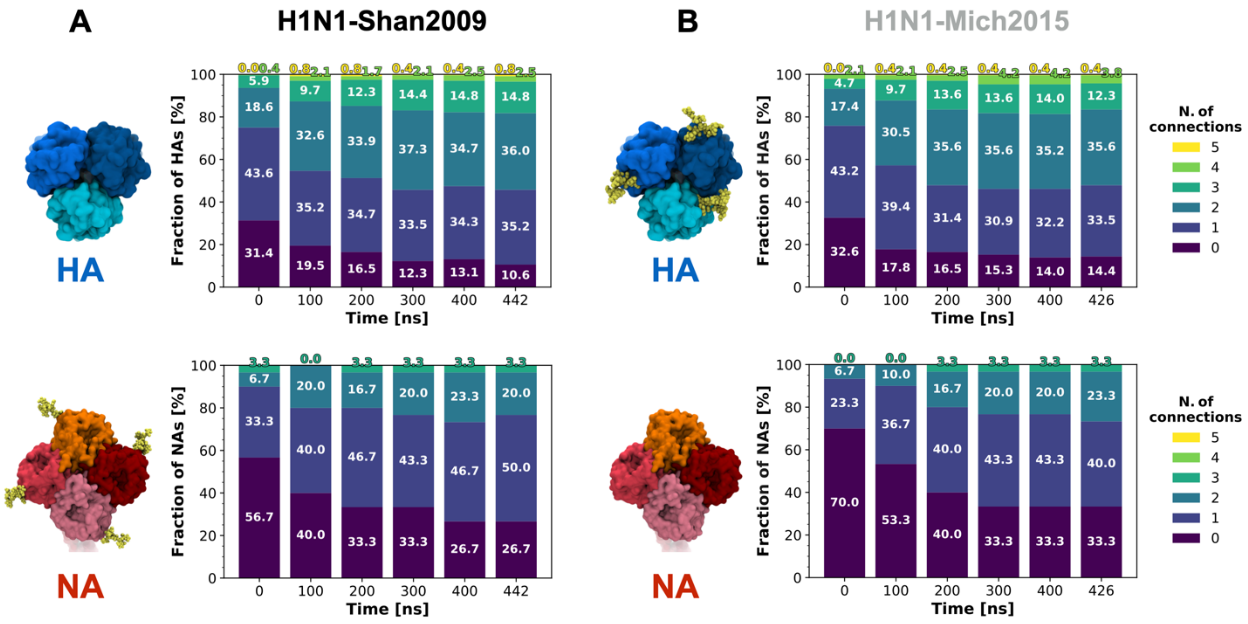

Figure S26. Analysis of glycoprotein interconnections for HA and NA. Stacked bar plots representing the fraction (%) of glycoproteins (HA top panels, NA bottom panels) establishing a certain number of connections (0 to 5, accordingly colored using the viridis palette) with surrounding glycoproteins along the simulation of (**A**) H1N1-Shan2009 and (**B**) H1N1-Mich2015. Percentages rounded to the first decimal digit are reported. A molecular representation of the HA head and NA head is also provided for both strains on the left side of each bar plot, highlighting with yellow vdW spheres the glycan that is added/deleted in H1N1-Mich2015: +1 glycan (N179) in H1N1-Mich2015 HA head and -1 glycan (N386) in H1N1-Mich2015 NA head.

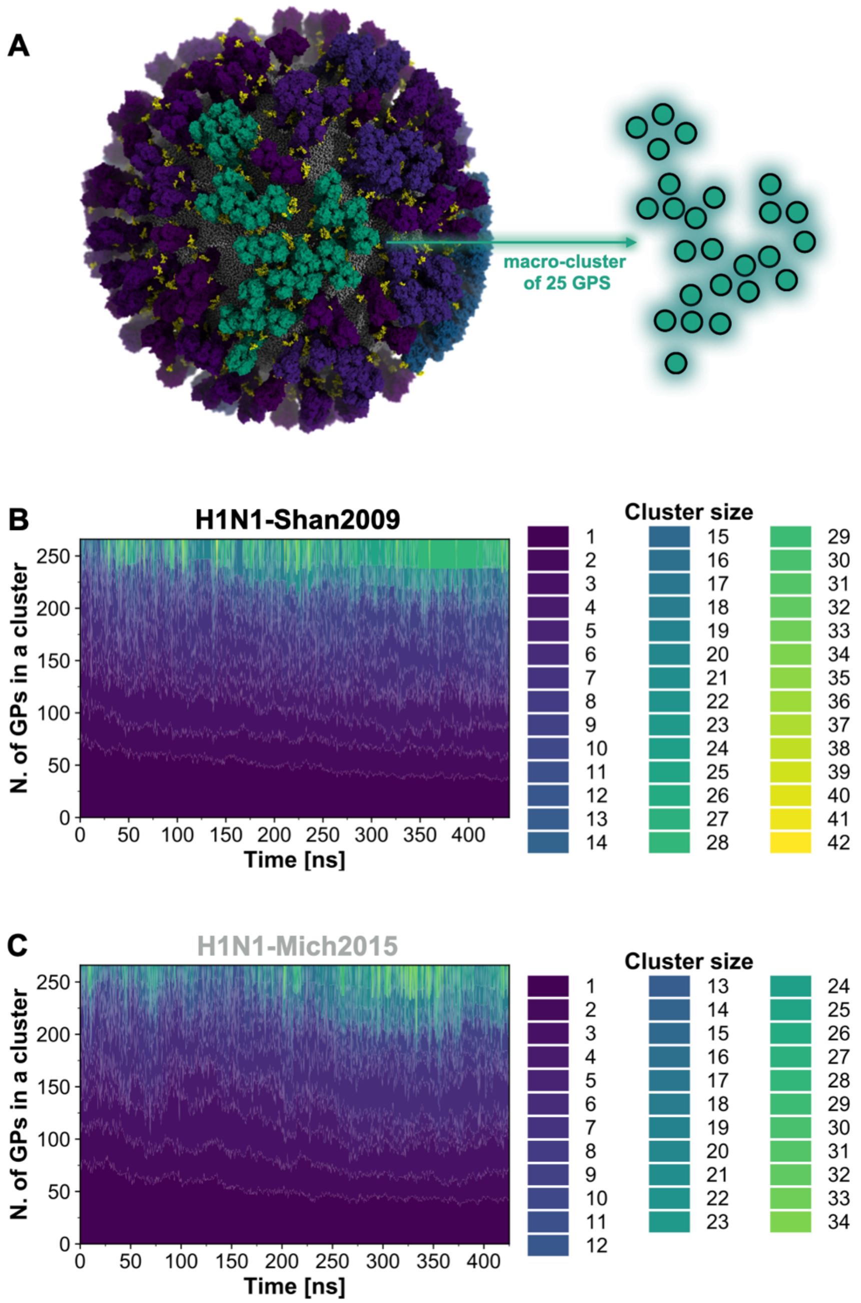

Figure S27. Analysis of glycoprotein macrocluster formation. **A**) Molecular representation of the H1N1-Shan2009 virion at a frame of the simulation where a macrocluster of 25 glycoproteins (GPs) is formed. The glycoproteins are depicted with a surface colored using a viridis palette according to the size of the macro-cluster that they belong to. The pattern of interactions of the macro-cluster of 25 GPs is highlighted using colored circles in the schematic on the right. **B-C**) Stacked plots representing the number of GPs involved in a macrocluster of a certain size along the simulation of (**B**) H1N1-Shan2009 and (**C**) H1N1-Mich2015. Macroclusters are colored using the *viridis* palette according to their size. The color gradient used for H1N1-Mich2015 (cluster size range: 0 to 34) has been normalized to the color gradient of H1N1-Shan2009 (cluster size range: 0 to 42).

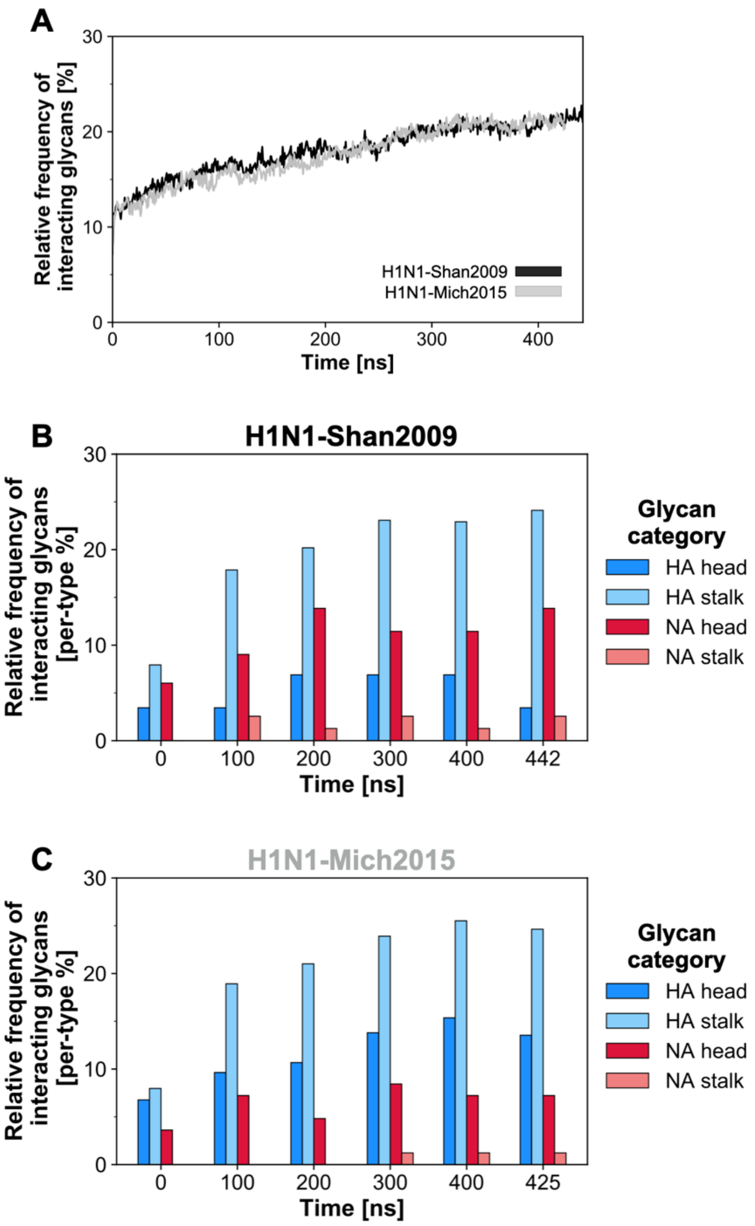

Figure S28. Analysis of glycoprotein connections involving glycans. **A**) Time evolution (ns) of the relative frequency (%) of interacting glycans, i.e., glycans participating in glycoprotein interconnections. The relative frequency of interacting glycan was calculated with respect to the total number of glycans. **B**) Grouped bar plot depicting the time evolution (ns) of the relative frequency (%) of interacting glycans in H1N1-Shan2009, where glycans have been divided into "head" and "stalk" categories according to their position, both for HA (shades of blue) and NA (shades of red). The relative frequency for each category of interacting glycans was calculated with respect to the total number of glycans of the same category. **C**) Grouped bar plot depicting the time evolution (ns) of the relative frequency (%) of interacting glycans in H1N1-Mich2015, where glycans have been divided into "head" and "stalk" categories according to their position, both for HA (shades of blue) and NA (shades of red). The relative frequency for each category of interacting glycans was calculated with respect to the total number of glycans of the same category.

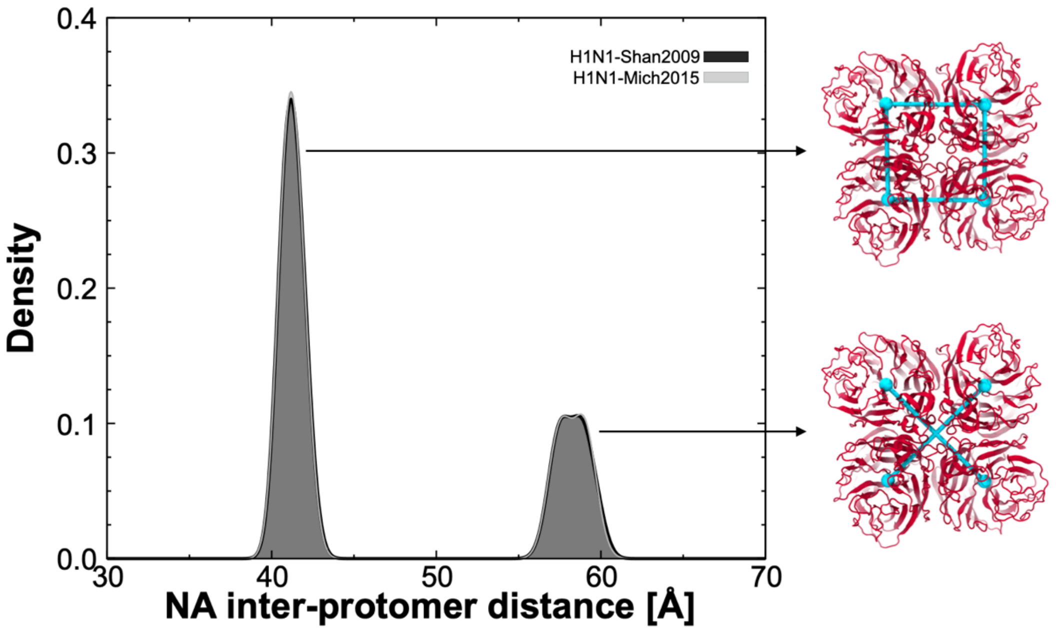

Figure S29. Analysis of NA head breathing. Distribution of inter-protomer distances between the four NA head COMs in H1N1-Shan2009 (black) and H1N1-Mich2015 (gray) virions. The distribution of the values is shown as a kernel density. On the right, a top view molecular representation of the NA head using red cartoons is provided, with the monitored distances between NA head COMs highlighted with cyan cylinders.

3. Supplementary Tables

Table S1.

Tilt angles (degree) of ten tilted and untilted structures (microstates) extracted from the MSM of the NA head tilting. Note: the average is the average of the ten selected microstates (ten selected conformational states and their respective angles). This will be representative of the average tilt angle of all microstates assigned to a macrostate of interest.

**NA HEAD-TILT ANGLES [°]**				
	**H1N1-Shan2009**		**H1N1-Mich2015**	
**Structure #**	**Untilted**	**Tilted**	**Untilted**	**Tilted**
**#1**	18.9°	81.9°	13.5°	90.0°
**#2**	20.3°	75.6°	19.7°	90.7°
**#3**	24.0°	76.7°	13.1°	88.1°
**#4**	22.9°	81.6°	14.4°	87.4°
**#5**	15.2°	79.5°	21.1°	89.7°
**#6**	23.6°	75.2°	14.4°	91.3°
**#7**	17.5°	74.2°	14.4°	87.0°
**#8**	22.4°	75.8°	20.7°	86.4°
**#9**	17.5°	78.1°	18.4°	89.6°
**#10**	32.5°	74.1°	22.1°	92.0°
**average**	**21.5°**	**77.3°**	**17.2°**	**89.2°**

Table S2.

Tilt angles (degree) of ten tilted and untilted structures (microstates) extracted from the MSM of the HA ectodomain tilting. Note: the average is the average of the ten selected microstates (ten selected conformational states and their respective angles). This will be representative of the average tilt angle of all microstates assigned to a macrostate of interest.

HA ECTODOMAIN-TILT ANGLES [°]				
	H1N1-Shan2009		H1N1-Mich2015	
**Structure #**	**Untilted**	**Tilted**	**Untilted**	**Tilted**
**#1**	8.1°	57.0°	19.1°	66.0°
**#2**	9.6°	57.3°	7.3°	61.9°
**#3**	10.0°	54.5°	19.8°	65.1°
**#4**	12.2°	57.3°	18.9°	61.3°
**#5**	5.9°	55.8°	5.9°	62.4°
**#6**	7.8°	56.9°	22.9°	66.2°
**#7**	21.1°	58.8°	6.2°	59.1°
**#8**	7.4°	58.2°	10.0°	61.8°
**#9**	9.8°	57.0°	14.8°	57.7°
**#10**	4.7°	55.3°	19.8°	67.9°
**average**	**9.7°**	**56.8°**	**14.5°**	**62.9°**

Table S3.

The table reports the distance between the COM of the HA monomer head (residues 66 to 286) and the COM of LαHs' apical residue (residues 419) calculated for open and closed structures (microstates) extracted from the MSM of the HA head breathing. The distances indicated here are the largest seen in each microstate, i.e., the largest of the three distances existing within the conformation of the HA trimer represented by the microstate. This applies also to the "closed" microstates, where the reported distance is still the largest distance seen in those microstates. Note: the average is the average of the ten selected microstates (ten selected conformational states and their respective angles). This will be representative of the average tilt angle of all microstates assigned to a macrostate of interest.

**HA HEAD COM–LαHs apical residue COM DISTANCE [Å]**				
	**H1N1-Shan2009**		**H1N1-Mich2015**	
**Structure #**	**<u>Closed</u>**	**<u>Open</u>**	**<u>Closed</u>**	**<u>Open</u>**
**#1**	27.3 Å	38.8 Å	27.3 Å	35.9 Å
**#2**	27.4 Å	37.8 Å	26.9 Å	42.2 Å
**#3**	28.1 Å	39.1 Å	27.0 Å	37.4 Å
**#4**	27.7 Å	38.7 Å	27.0 Å	36.6 Å
**#5**	27.6 Å	37.8 Å	26.9 Å	35.7 Å
**#6**	27.6 Å	36.7 Å	27.5 Å	37.3 Å
**#7**	27.4 Å	36.5 Å	26.9 Å	39.3 Å
**#8**	28.3 Å	36.7 Å	27.1 Å	35.6 Å
**#9**	28.3 Å	37.8 Å	27.2 Å	39.3 Å
**#10**	28.3 Å	36.0 Å	27.0 Å	36.5 Å
**average**	**27.8 Å**	**37.6 Å**	**27.1 Å**	**37.6 Å**

Table S4. P(A|B) for HA ectodomain tilting, where event A is the achievement of a certain tilt-angle swing range by the HA ectodomain during the dynamics with respect to the initial frame (calculated as *tilt-angle_frame_n – tilt-angle_frame_0*, with only positive values counted), whereas event B is the formation of one or more connections during the dynamics.

	H1N-Shan2009		H1N-Mich2015	
**EVENTS**	**P(A)**	**P(A|B)**	**P(A)**	**P(A|B)**
**A:** 0º ≤ **HA** tilt-angle swing range < 10º<br>**B** = 1 or more connections	45.2 ± 0.2 %	44.6 ± 0.3 %	45.8 ± 0.2 %	45.7 ± 0.3 %
**A:** 10º ≤ **HA** tilt-angle swing range < 20º<br>**B** = 1 or more connections	19.2 ± 0.1 %	19.8 ± 0.2 %	17.6 ± 0.1 %	18.5 ± 0.2 %
**A:** 20º ≤ **HA** tilt-angle swing range < 30º<br>**B** = 1 or more connections	3.9 ± 0.0 %	4.3 ± 0.1 %	4.4 ± 0.1 %	4.5 ± 0.1 %
**A:** 30º ≤ **HA** tilt-angle swing range<br>**B** = 1 or more connections	0.3 ± 0.0 %	0.4 ± 0.0 %	0.6 ± 0.0 %	0.6 ± 0.0 %

Table S5. P(A|B) for HA head breathing, where event A is the achievement of a certain extent of breathing by at least one of the HA head monomer within the same HA trimer during the dynamics with respect to the initial frame (calculated as *breathingdistance_frame_n – breathingdistance_frame_0*, with only positive values counted), whereas event B is the formation of one or more connections during the dynamics.

	H1N-Shan2009		H1N-Mich2015	
**EVENTS**	**P(A)**	**P(A|B)**	**P(A)**	**P(A|B)**
**A:** 1.0 Å ≤ breathing of one HA head < 3.0 Åº<br>**B:** 1 or more connections	46.9 ± 0.2 %	46.7 ± 0.3%	46.2 ± 0.2 %	46.1 ± 0.3%
**A:** 3.0 Å ≤ breathing of one HA head < 5.0 Åº<br>**B:** 1 or more connections	11.2 ± 0.1 %	11.5 ± 0.1 %	11.0 ± 0.1 %	10.9 ± 0.1 %
**A:** 5.0 Å ≤ breathing of one HA head < 7.0 Åº<br>**B:** 1 or more connections	2.2 ± 0.0 %	2.4 ± 0.1 %	2.8 ± 0.0 %	2.8 ± 0.1 %
**A:** 7.0 Å ≤ breathing of one HA head<br>**B:** 1 or more connections	0.2 ± 0.0 %	0.2 ± 0.0 %	0.3 ± 0.0 %	0.3 ± 0.0 %

Table S6.

P(A|B) for NA head tilting, where event A is the achievement of a certain tilt-angle swing range by the NA head during the dynamics with respect to the initial frame (calculated as *tilt-angle_frame_n – tilt-angle_frame_0*, with only positive values counted), whereas event B is the formation of one or more connections during the dynamics.

	H1N-Shan2009		H1N-Mich2015	
**EVENTS**	**P(A)**	**P(A|B)**	**P(A)**	**P(A|B)**
**A:** 0º ≤ **NA** head tilt-angle swing range < 10º<br>**B:** 1 or more connections	32.1 ± 0.4 %	34.6 ± 0.7 %	29.9 ± 0.4 %	27.5 ± 0.6 %
**A:** 10º ≤ **NA** head tilt-angle swing range < 20º<br>**B:** 1 or more connections	18.9 ± 0.3%	18.0 ± 0.5 %	14.3 ± 0.3 %	18.8 ± 0.5 %
**A:** 20º ≤ **NA** head tilt-angle swing range < 20º<br>**B:** 1 or more connections	6.0 ± 0.2 %	4.9 ± 0.2 %	6.5 ± 0.2 %	8.2 ± 0.3 %
**A:** 30º ≤ **NA** head tilt-angle swing range < 40º<br>**B:** 1 or more connections	2.4 ± 0.1 %	2.5 ± 0.2 %	6.9 ± 0.2 %	8.0 ± 0.3 %
**A:** 40º ≤ **NA** head tilt-angle swing range<br>**B:** 1 or more connections	1.8 ± 0.1 %	2.0 ± 0.2 %	1.2 ± 0.1 %	0.9 ± 0.1 %

Table S7. P(B|A) for HA ectodomain tilting, where event A is the achievement of a certain tilt-angle swing range by the HA ectodomain during the dynamics with respect to the initial frame (calculated as *tilt-angle_frame_n – tilt-angle_frame_0*, with only positive values counted), whereas event B is the formation of one or more connections during the dynamics.

	**H1N-Shan2009**		**H1N-Mich2015**	
**EVENTS**	**P(B)**	**P(B|A)**	**P(B)**	**P(B|A)**
**A:** 10º ≤ **HA** tilt-angle swing range < 20º<br>**B:** 1 or more connections	82.9 ± 0.2 %	81.8 ± 0.5 %	83.4 ± 0.2 %	83.1 ± 0.5 %
**A:** 10º ≤ **HA** tilt-angle swing range < 20º<br>**B:** 1 or more connections	82.9 ± 0.2 %	85.5 ± 0.7 %	83.4 ± 0.2 %	87.8 ± 0.8 %
**A:** 20º ≤ **HA** tilt-angle swing range < 30º<br>**B:** 1 or more connections	82.9 ± 0.2 %	91.1 ± 1.6 %	83.4 ± 0.2 %	85.5 ± 1.5 %
**A:** 30º ≤ **HA** tilt-angle swing range<br>**B:** 1 or more connections	82.9 ± 0.2 %	99.8 ± 5.8 %	83.4 ± 0.2 %	95.1 ± 4.4 %

Table S8. P(B|A) for HA head breathing, where event A is the achievement of a certain extent of breathing by at least one of the HA head monomer within the same HA trimer during the dynamics with respect to the initial frame (calculated as *breathingdistance_frame_n – breathingdistance_frame_0*, with only positive values counted), whereas event B is the formation of one or more connections during the dynamics.

	**H1N-Shan2009**		**H1N-Mich2015**	
**EVENTS**	**P(A)**	**P(B|A)**	**P(A)**	**P(B|A)**
**A:** 1.0 Å ≤ breathing of one HA head < 3.0 Åº<br>**B:** 1 or more connections	82.9 ± 0.2 %	82.5 ± 0.5 %	83.4 ± 0.2 %	83.2 ± 0.5 %
**A:** 3.0 Å ≤ breathing of one HA head < 5.0 Åº<br>**B:** 1 or more connections	82.9 ± 0.2 %	85.2 ± 0.9 %	83.4 ± 0.2 %	82.8 ± 0.9 %
**A:** 5.0 Å ≤ breathing of one HA head < 7.0 Åº<br>**B:** 1 or more connections	82.9 ± 0.2 %	91.5 ± 2.2 %	83.4 ± 0.2 %	84.5 ± 1.8 %
**A:** 7.0 Å ≤ breathing of one HA head<br>**B:** 1 or more connections	82.9 ± 0.2 %	97.1 ± 7.4 %	83.4 ± 0.2 %	81.8 ± 5.3 %

Table S9. P(B|A) for NA head tilting, where event A is the achievement of a certain tilt-angle swing range by the NA head during the dynamics with respect to the initial frame (calculated as *tilt-angle_frame_n – tilt-angle_frame_0*, with only positive values counted), whereas event B is the formation of one or more connections during the dynamics.

	**H1N-Shan2009**		**H1N-Mich2015**	
**EVENTS**	**P(B)**	**P(B|A)**	**P(B)**	**P(B|A)**
**A:** 0º ≤ **NA** head tilt-angle swing range < 10º<br>**B:** 1 or more connections	65.8 ± 0.5 %	71.1 ± 1.4 %	56.6 ± 0.4 %	52.1 ± 1.2 %
**A:** 10º ≤ **NA** head tilt-angle swing range < 20º<br>**B:** 1 or more connections	65.8 ± 0.5 %	62.5 ± 1.6 %	56.6 ± 0.5 %	74.3 ± 2.2 %
**A:** 20º ≤ **NA** head tilt-angle swing range < 20º<br>**B:** 1 or more connections	65.8 ± 0.5 %	54.3 ± 2.6 %	56.6 ± 0.5 %	70.8 ± 3.0 %
**A:** 30º ≤ **NA** head tilt-angle swing range < 40º<br>**B:** 1 or more connections	65.8 ± 0.5 %	67.1 ± 4.6 %	56.6 ± 0.5 %	65.5 ± 2.8 %
**A:** 40º ≤ **NA** head tilt-angle swing range<br>**B:** 1 or more connections	65.8 ± 0.5 %	73.5 ± 5.6 %	56.6 ± 0.5 %	45.0 ± 5.2 %

4. Captions for Supplementary Movies

Movie S1.

All-atom MD simulation of the glycosylated model of H1N1-Shan2009 influenza A virion. ~442 ns of MD are shown. Neuraminidase (NA) and hemagglutinin (HA) glycoproteins, and the M2 ion channels, are depicted with red, blue, and white surfaces, respectively. N-linked glycans are shown in yellow. The spherical lipid envelope is represented with pink van der Waals (vdW) spheres.

Movie S2.

All-atom MD simulation of the glycosylated model of H1N1-Mich2015 influenza A virion. ~425 ns of MD are shown. Neuraminidase (NA) and hemagglutinin (HA) glycoproteins, and the M2 ion channels, are depicted with red, blue, and white surfaces, respectively. N-linked glycans are shown in yellow. The spherical lipid envelope is represented with pink van der Waals (vdW) spheres.

Movie S3.

NA head tilting motion. The head tilting motion of a representative NA tetramer extracted from the H1N1-Shan2009 whole-virion simulation is displayed. NA tetramer is depicted with a transparent surface overlaid on top of a cartoon representation colored with a viridis palette according to the extent of head tilt. N-linked glycans and sequon asparagine are shown with yellow van der Waals (vdW) spheres.

Movie S4.

NA head tilting motion. The head tilting motion of a NA tetramer is displayed together with the dynamics of the surrounding crowded environment of the H1N1-Shan2009 whole-virion. ~442 ns of MD are shown. Neuraminidase (NA) and hemagglutinin (HA) glycoproteins, and the M2 ion channels, are depicted with red, blue, and white surfaces, respectively. N-linked glycans are shown in yellow. The spherical lipid envelope is represented with pink van der Waals (vdW) spheres. The NA exhibiting head tilting motion is highlighted in red during the dynamics, whereas the other HAs and NAs are colored with gray and dark gray, respectively.

Movie S5.

HA ectodomain tilting motion. The ectodomain tilting motion of a representative HA trimer extracted from the H1N1-Shan2009 whole-virion simulation is displayed. HA trimer is depicted with a transparent surface overlaid on top of a cartoon representation colored with a viridis palette according to the extent of ectodomain tilt. N-linked glycans and sequon asparagine are shown with yellow van der Waals (vdW) spheres.

Movie S6.

HA ectodomain tilting motion. The ectodomain tilting motion of an HA trimer is displayed together with the dynamics of the surrounding crowded environment of the H1N1-Shan2009 whole-virion. ~442 ns of MD are shown. Neuraminidase (NA) and hemagglutinin (HA)

glycoproteins, and the M2 ion channels, are depicted with red, blue, and white surfaces, respectively. N-linked glycans are shown in yellow. The spherical lipid envelope is represented with pink van der Waals (vdW) spheres. The HA exhibiting ectodomain tilting motion is highlighted in blue during the dynamics, whereas the other HAs and NAs are colored with gray and dark gray, respectively.

Movie S7.

HA head breathing motion. The head breathing motion of a representative HA trimer extracted from the H1N1-Shan2009 whole-virion simulation is displayed from a side (left) and a top (right) viewpoint simultaneously. Each HA monomer within the trimer is depicted with a transparent surface overlaid on top of a cartoon representation colored with a viridis palette according to the extent of the respective head breathing. N-linked glycans and sequon asparagine are shown with yellow van der Waals (vdW) spheres.

Movie S8.

HA head breathing motion. The head breathing motion of an HA trimer is displayed together with the dynamics of the surrounding crowded environment of the H1N1-Shan2009 whole-virion. ~442 ns of MD are shown. Neuraminidase (NA) and hemagglutinin (HA) glycoproteins, and the M2 ion channels, are depicted with red, blue, and white surfaces, respectively. N-linked glycans are shown in yellow. The spherical lipid envelope is represented with pink van der Waals (vdW) spheres. The HA exhibiting head breathing motion is highlighted in blue during the dynamics, whereas the other HAs and NAs are colored with gray and dark gray, respectively.

Movie S9.

The dynamic interplay between glycoproteins. The glycoprotein interplay taking place during the simulations of the glycosylated whole-virion models of H1N1-Shan2009 (left) and H1N1-Mich2015 (right) is displayed. ~442 ns and ~425 ns of MD are shown, respectively. The glycoproteins are depicted with a surface colored according to the number of connections made by the respective glycoprotein at that frame (0 to 5, colored in viridis palette). Interacting glycans are highlighted with yellow vdW spheres, whereas non-interacting glycans with white VdW spheres.

Movie S10.

Glycoprotein macrocluster formation. The formation of macrocluster of glycoproteins taking place during the simulations of the glycosylated whole-virion models of H1N1-Shan2009 (left) and H1N1-Mich2015 (right) is displayed. ~442 ns and ~425 ns of MD are shown, respectively. The glycoproteins are depicted with a surface colored according to the size of the macrocluster they are involved in at that frame (colored in *viridis* palette). All glycans are highlighted with white vdW spheres.

5. Captions for Supplementary Data

Data S1.

Jupyter-notebooks for the MSM analysis of NA head tilting, HA ectodomain tilting, and HA head breathing are provided along with PDB files of representative NA/HA conformations extracted for each state.

Data S2.

3D map visualization of Fab NDS.1 in complex with A/Darwin/9/2021 at 24.1 Å resolution. Density file (.mrc).

6. Supplementary References

(1) Durrant, J. D.; Kochanek, S. E.; Casalino, L.; Ieong, P. U.; Dommer, A. C.; Amaro, R. E. Mesoscale All-Atom Influenza Virus Simulations Suggest New Substrate Binding Mechanism. *ACS Cent. Sci.* **2020**, *6* (2), 189–196.

(2) Durrant, J. D.; Amaro, R. E. LipidWrapper: An Algorithm for Generating Large-Scale Membrane Models of Arbitrary Geometry. *PLoS Comput. Biol.* **2014**, *10* (7), e1003720.

(3) Jo, S.; Lim, J. B.; Klauda, J. B.; Im, W. CHARMM-GUI Membrane Builder for Mixed Bilayers and Its Application to Yeast Membranes. *Biophys. J.* **2009**, *97* (1), 50.

(4) Jo, S.; Kim, T.; Im, W. Automated Builder and Database of Protein/Membrane Complexes for Molecular Dynamics Simulations. *PLoS One* **2007**, *2* (9), e880.

(5) Vermaas, J. v.; Mayne, C. G.; Shinn, E.; Tajkhorshid, E. Assembly and Analysis of Cell-Scale Membrane Envelopes. *J. Chem. Inf. Model.* **2022**, *62* (3), 602–617.

(6) Ivanova, P. T.; Myers, D. S.; Milne, S. B.; McClaren, J. L.; Thomas, P. G.; Brown, H. A. Lipid Composition of Viral Envelope of Three Strains of Influenza Virus – Not All Viruses Are Created Equal. *ACS Infect. Dis.* **2015**, *1* (9), 399–452.

(7) Amaro, R. E.; Ieong, P. U.; Huber, G.; Dommer, A.; Steven, A. C.; Bush, R. M.; Durrant, J. D.; Votapka, L. W. A Computational Assay That Explores the Hemagglutinin/Neuraminidase Functional Balance Reveals the Neuraminidase Secondary Site as a Novel Anti-Influenza Target. *ACS Cent. Sci.* **2018**, *4* (11), 1570–1577.

(8) Harris, A.; Cardone, G.; Winkler, D. C.; Heymann, J. B.; Brecher, M.; White, J. M.; Steven, A. C. Influenza Virus Pleiomorphy Characterized by Cryoelectron Tomography. *Proc. Natl. Acad. Sci. U. S. A.* **2006**, *103* (50), 19123–19127.

(9) Durrant, J. D.; McCammon, J. A. AutoClickChem: Click Chemistry in Silico. *PLoS Comput. Biol.* **2012**, *8* (3), e1002397.

(10) Ropp, P.; Friedman, A.; Durrant, J. D. Scoria: A Python Module for Manipulating 3D Molecular Data. *J. Cheminform.* **2017**, *9* (1), 1–7.

(11) Jorgensen, W. L.; Chandrasekhar, J.; Madura, J. D.; Impey, R. W.; Klein, M. L. Comparison of Simple Potential Functions for Simulating Liquid Water. *J. Chem. Phys.* **1998**, *79* (2), 926.

(12) Phillips, J. C.; Hardy, D. J.; Maia, J. D. C.; Stone, J. E.; Ribeiro, J. v.; Bernardi, R. C.; Buch, R.; Fiorin, G.; Hénin, J.; Jiang, W.; et al. Scalable Molecular Dynamics on CPU and GPU Architectures with NAMD. *J. Chem. Phys.* **2020**, *153* (4), 044130.

(13) Huang, J.; Mackerell, A. D. CHARMM36 All-Atom Additive Protein Force Field: Validation Based on Comparison to NMR Data. *J. Comput. Chem.* **2013**, *34* (25), 2135–2145.

(14) Klauda, J. B.; Venable, R. M.; Freites, J. A.; O'Connor, J. W.; Tobias, D. J.; Mondragon-Ramirez, C.; Vorobyov, I.; MacKerell, A. D.; Pastor, R. W. Update of the CHARMM All-Atom Additive Force Field for Lipids: Validation on Six Lipid Types. *J. Phys. Chem. B* **2010**, *114* (23), 7830–7843.

(15) Klauda, J. B.; Monje, V.; Kim, T.; Im, W. Improving the CHARMM Force Field for Polyunsaturated Fatty Acid Chains. *J. Phys. Chem. B* **2012**, *116* (31), 9424–9431.

(16) Beglov, D.; Roux, B. Finite Representation of an Infinite Bulk System: Solvent Boundary Potential for Computer Simulations. *J. Chem. Phys.* **1998**, *100* (12), 9050.

(17) Sharma, M.; Yi, M.; Dong, H.; Qin, H.; Peterson, E.; Busath, D. D.; Zhou, H. X.; Cross, T. A. Insight into the Mechanism of the Influenza A Proton Channel from a Structure in a Lipid Bilayer. *Science (1979)* **2010**, *330* (6003), 509–512.

(18) Sun, S.; Wang, Q.; Zhao, F.; Chen, W.; Li, Z. Glycosylation Site Alteration in the Evolution of Influenza A (H1N1) Viruses. *PLoS One* **2011**, *6* (7), e22844.

(19) Blake, T. A.; Williams, T. L.; Pirkle, J. L.; Barr, J. R. Targeted N-Linked Glycosylation Analysis of H5N1 Influenza Hemagglutinin by Selective Sample Preparation and Liquid Chromatography/Tandem Mass Spectrometry. *Anal. Chem.* **2009**, *81* (8), 3109–3118.

(20) Kirkpatrick, E.; Qiu, X.; Wilson, P. C.; Bahl, J.; Krammer, F. The Influenza Virus Hemagglutinin Head Evolves Faster than the Stalk Domain. *Sci. Rep.* **2018**, *8* (1), 10432.

(21) Pralow, A.; Hoffmann, M.; Nguyen-Khuong, T.; Pioch, M.; Hennig, R.; Genzel, Y.; Rapp, E.; Reichl, U. Comprehensive N-Glycosylation Analysis of the Influenza A Virus Proteins HA and NA from Adherent and Suspension MDCK Cells. *FEBS J.* **2021**, *288* (16), 4869–4891.

(22) Cruz, E.; Cain, J.; Crossett, B.; Kayser, V. Site-Specific Glycosylation Profile of Influenza A (H1N1) Hemagglutinin through Tandem Mass Spectrometry. *Hum. Vaccin. Immunother.* **2017**, *14* (3), 508–517.

(23) Khatri, K.; Klein, J. A.; White, M. R.; Grant, O. C.; Leymarie, N.; Woods, R. J.; Hartshorn, K. L.; Zaia, J. Integrated Omics and Computational Glycobiology Reveal Structural Basis for Influenza a Virus Glycan Microheterogeneity and Host Interactions. *Mol. Cell. Proteomics* **2016**, *15* (6), 1895–1912.

(24) Liu, Y. J.; Wu, S. L.; Love, K. R.; Hancock, W. S. Characterization of Site-Specific Glycosylation in Influenza A Virus Hemagglutinin Produced by Spodoptera Frugiperda Insect Cell Line. *Anal. Chem.* **2017**, *89* (20), 11036–11043.

(25) She, Y. M.; Farnsworth, A.; Li, X.; Cyr, T. D. Topological N-Glycosylation and Site-Specific N-Glycan Sulfation of Influenza Proteins in the Highly Expressed H1N1 Candidate Vaccines. *Sci. Rep.* **2017**, *7* (1), 10232.

(26) Danne, R.; Poojari, C.; Martinez-Seara, H.; Rissanen, S.; Lolicato, F.; Róg, T.; Vattulainen, I. DoGlycans-Tools for Preparing Carbohydrate Structures for Atomistic Simulations of Glycoproteins, Glycolipids, and Carbohydrate Polymers for GROMACS. *J. Chem. Inf. Model.* **2017**, *57* (10), 2401–2406.

(27) Humphrey, W.; Dalke, A.; Schulten, K. VMD: Visual Molecular Dynamics. *J. Mol. Graph.* **1996**, *14* (1), 33–38.

(28) Phillips, J. C.; Braun, R.; Wang, W.; Gumbart, J.; Tajkhorshid, E.; Villa, E.; Chipot, C.; Skeel, R. D.; Kalé, L.; Schulten, K. Scalable Molecular Dynamics with NAMD. *J. Comput. Chem.* **2005**, *26* (16), 1781–1802.

(29) Abraham, M. J.; Murtola, T.; Schulz, R.; Páll, S.; Smith, J. C.; Hess, B.; Lindah, E. GROMACS: High Performance Molecular Simulations through Multi-Level Parallelism from Laptops to Supercomputers. *SoftwareX* **2015**, *1–2*, 19–25.

(30) Li, J.; Liu, S.; Gao, Y.; Tian, S.; Yang, Y.; Ma, N. Comparison of N-Linked Glycosylation on Hemagglutinins Derived from Chicken Embryos and MDCK Cells: A Case of the Production of a Trivalent Seasonal Influenza Vaccine. *Appl. Microbiol. Biotechnol.* **2021**, *105* (9), 3559–3572.

(31) Liu, Y. J.; Wu, S. L.; Love, K. R.; Hancock, W. S. Characterization of Site-Specific Glycosylation in Influenza A Virus Hemagglutinin Produced by Spodoptera Frugiperda Insect Cell Line. *Anal. Chem.* **2017**, *89* (20), 11036–11043.

(32) Schnell, J. R.; Chou, J. J. Structure and Mechanism of the M2 Proton Channel of Influenza A Virus. *Nature* **2008**, *451* (7178), 591–595.

(33) Pielak, R. M.; Chou, J. J. Influenza M2 Proton Channels. *Biochim. Biophys. Acta Biomembr.* **2011**, *1808* (2), 522–529.

(34) Guvench, O.; Hatcher, E.; Venable, R. M.; Pastor, R. W.; MacKerell, A. D. CHARMM Additive All-Atom Force Field for Glycosidic Linkages between Hexopyranoses. *J. Chem. Theory Comput.* **2009**, *5* (9), 2353–2370.

(35) *Preparing large MD simulations with VMD*. https://www.ks.uiuc.edu/Research/vmd/minitutorials/largesystems/ (accessed 2022-06-01).

(36) Brünger, A.; Brooks, C. L.; Karplus, M. Stochastic Boundary Conditions for Molecular Dynamics Simulations of ST2 Water. *Chem. Phys. Lett.* **1984**, *105* (5), 495–500.

(37) Martyna, G. J.; Tobias, D. J.; Klein, M. L. Constant Pressure Molecular Dynamics Algorithms. *J. Chem. Phys.* **1998**, *101* (5), 4177.

(38) Feller, S. E.; Zhang, Y.; Pastor, R. W.; Brooks, B. R. Constant Pressure Molecular Dynamics Simulation: The Langevin Piston Method. *J. Chem. Phys.* **1998**, *103* (11), 4613.

(39) Darden, T.; York, D.; Pedersen, L. Particle Mesh Ewald: An N·log(N) Method for Ewald Sums in Large Systems. *J. Chem. Phys.* **1998**, *98* (12), 10089.

(40) Ryckaert, J. P.; Ciccotti, G.; Berendsen, H. J. C. Numerical Integration of the Cartesian Equations of Motion of a System with Constraints: Molecular Dynamics of n-Alkanes. *J. Comput. Phys.* **1977**, *23* (3), 327–341.

(41) Williams, T.; Kelley, C.; Bersch, C.; Bröker, H.-B.; Campbell, J.; Cunningham, R.; Denholm, D.; Elber, G.; Fearick, R.; Grammes, C.; et al. *Gnuplot 5.4 An Interactive Plotting Program*; 1986.

(42) Bangaru, S.; Lang, S.; Schotsaert, M.; Vanderven, H. A.; Zhu, X.; Kose, N.; Bombardi, R.; Finn, J. A.; Kent, S. J.; Gilchuk, P.; et al. A Site of Vulnerability on the Influenza Virus Hemagglutinin Head Domain Trimer Interface. *Cell* **2019**, *177* (5), 1136-1152.

(43) Shrake, A.; Rupley, J. A. Environment and Exposure to Solvent of Protein Atoms. Lysozyme and Insulin. *J. Mol. Biol.* **1973**, *79* (2), 351–371.

(44) Scherer, M. K.; Trendelkamp-Schroer, B.; Paul, F.; Pérez-Hernández, G.; Hoffmann, M.; Plattner, N.; Wehmeyer, C.; Prinz, J. H.; Noé, F. PyEMMA 2: A Software Package for Estimation, Validation, and Analysis of Markov Models. *J. Chem. Theory Comput.* **2015**, *11* (11), 5525–5542.

(45) Pérez-Hernández, G.; Paul, F.; Giorgino, T.; de Fabritiis, G.; Noé, F. Identification of Slow Molecular Order Parameters for Markov Model Construction. *J. Chem. Phys.* **2013**, *139* (1), 015102.

(46) Molgedey, L.; Schuster, H. G. Separation of a Mixture of Independent Signals Using Time Delayed Correlations. *Phys. Rev. Lett.* **1994**, *72* (23), 3634.

(47) Schwantes, C. R.; Pande, V. S. Improvements in Markov State Model Construction Reveal Many Non-Native Interactions in the Folding of NTL9. *J. Chem. Theory Comput.* **2013**, *9* (4), 2000–2009.

(48) Hochbaum D; Shmoys D. A Best Possible Heuristic for the K-Center Problem. *Math. Oper. Res.* **1985**, *10* (2), 180–184.

(49) Swope, W. C.; Pitera, J. W.; Suits, F. Describing Protein Folding Kinetics by Molecular Dynamics Simulations. 1. Theory. *J. Phys. Chem. B* **2004**, *108* (21), 6571–6581.

(50) Trendelkamp-Schroer, B.; Wu, H.; Paul, F.; Noé, F. Estimation and Uncertainty of Reversible Markov Models. *J. Chem. Phys.* **2015**, *143* (17), 174101.

(51) Prinz, J. H.; Wu, H.; Sarich, M.; Keller, B.; Senne, M.; Held, M.; Chodera, J. D.; Schtte, C.; Noé, F. Markov Models of Molecular Kinetics: Generation and Validation. *J. Chem. Phys.* **2011**, *134* (17), 174105.

(52) Metzler, R. Generalized Chapman-Kolmogorov Equation: A Unifying Approach to the Description of Anomalous Transport in External Fields. *Phys. Rev. E* **2000**, *62* (5), 6233.

(53) Chapman, B. S. On the Brownian Displacements and Thermal Diffusion of Grains Suspended in a Non-Uniform Fluid. *Proc. R. Soc. Lond. A Math. Phys. Sci.* **1928**, *119* (781), 34–54.

(54) Kolmogoroff, A. Über Die Analytischen Methoden in Der Wahrscheinlichkeitsrechnung. *Math. Ann.* **1931**, *104* (1), 415–458.

(55) Röblitz, S.; Weber, M. Fuzzy Spectral Clustering by PCCA+: Application to Markov State Models and Data Classification. *Adv. Data Anal. Classif.* **2013**, *7* (2), 147–179.

(56) McGibbon, R. T.; Beauchamp, K. A.; Harrigan, M. P.; Klein, C.; Swails, J. M.; Hernández, C. X.; Schwantes, C. R.; Wang, L. P.; Lane, T. J.; Pande, V. S. MDTraj: A Modern Open Library for the Analysis of Molecular Dynamics Trajectories. *Biophys. J.* **2015**, *109* (8), 1528–1532.

(57) Stone, J. E.; Vandivort, K. L.; Schulten, K. GPU-Accelerated Molecular Visualization on Petascale Supercomputing Platforms. *Proceedings of the 8th International Workshop on Ultrascale Visualization* **2013**, 6.

(58) Doria-Rose, N. A.; Bhiman, J. N.; Roark, R. S.; Schramm, C. A.; Gorman, J.; Chuang, G.-Y.; Pancera, M.; Cale, E. M.; Ernandes, M. J.; Louder, M. K.; et al. New Member of the V1V2-Directed CAP256-VRC26 Lineage That Shows Increased Breadth and Exceptional Potency. *J. Virol.* **2015**, *90* (1), 76–91.

(59) Tiller, T.; Meffre, E.; Yurasov, S.; Tsuiji, M.; Nussenzweig, M. C.; Wardemann, H. Efficient Generation of Monoclonal Antibodies from Single Human B Cells by Single Cell RT-PCR and Expression Vector Cloning. *J. Immunol. Methods* **2008**, *329* (1–2), 112–124.

(60) Mastronarde, D. N. Automated Electron Microscope Tomography Using Robust Prediction of Specimen Movements. *J. Struct. Biol.* **2005**, *152* (1), 36–51.

(61) Scheres, S. H. W. RELION: Implementation of a Bayesian Approach to Cryo-EM Structure Determination. *J. Struct. Biol.* **2012**, *180* (3), 519–530.

(62) Pettersen, E. F.; Goddard, T. D.; Huang, C. C.; Couch, G. S.; Greenblatt, D. M.; Meng, E. C.; Ferrin, T. E. UCSF Chimera--a Visualization System for Exploratory Research and Analysis. *J. Comput. Chem.* **2004**, *25* (13), 1605–1612.

(63) Altschul, S. F.; Gish, W.; Miller, W.; Myers, E. W.; Lipman, D. J. Basic Local Alignment Search Tool. *J. Mol. Biol.* **1990**, *215* (3), 403–410.

(64) Stadlbauer, D.; Zhu, X.; McMahon, M.; Turner, J. S.; Wohlbold, T. J.; Schmitz, A. J.; Strohmeier, S.; Yu, W.; Nachbagauer, R.; Mudd, P. A.; et al. Broadly Protective Human

Antibodies That Target the Active Site of Influenza Virus Neuraminidase. *Science (1979)* **2019**, *366* (6464), 499–504.

(65) Saul, F. A.; Poljak, R. J. Crystal Structure of Human Immunoglobulin Fragment Fab New Refined at 2.0 Å Esolution. *Proteins* **1992**, *14* (3), 363–371.

(66) Benton, D. J.; Nans, A.; Calder, L. J.; Turner, J.; Neu, U.; Lin, Y. P.; Ketelaars, E.; Kallewaard, N. L.; Corti, D.; Lanzavecchia, A.; et al. Influenza Hemagglutinin Membrane Anchor. *Proc. Natl. Acad. Sci. U. S. A.* **2018**, *115* (40), 10112–10117.
