# Supplementary material for: Breathing and Tilting: Mesoscale Simulations Illuminate Influenza Glycoprotein Vulnerabilities
Source: ACS Cent Sci. 2022 Dec 8;8(12):1646–63. doi: 10.1021/acscentsci.2c00981 (PMC9801513; doi:10.1021/acscentsci.2c00981)

oc-2022-00981d.R1

Name: Peer Review Information for "Breathing and Tilting: Mesoscale simulations illuminate influenza glycoprotein vulnerabilities"

First Round of Reviewer Comments

Reviewer: 1

Comments to the Author

Amaro and co-workers present a tour-de-force molecular simulation study of the influenza virion which provides insights on the viral egress process via characterisation of specific dynamic modes.

The study builds upon previously published work from this group on influenza which provides confidence in their knowledge and handling of this system, and extends it to look at two evolutionarily linked viruses.

Markov state modelling is used to probe the system kinetics. Extensive supplementary information is provided to further support the conclusions of the work.

I commend the authors on an excellent study. Further, i think these types of simulations of large systems, which are carried out rigorously and with due consideration and discussion of caveats etc are essential for moving the field forward.

Minor comment:

Can Figure 1 be simplified at all? Its a bit too busy imho.

Reviewer: 2

Comments to the Author

The manuscript by Casalino and team describes all-atom molecular dynamics simulation of two solvated influenza A virion models. The data is analyzed using Markov State Models to study conformational transitions of the surface proteins, and seek the biological relevance of the hidden conformations seen in the MD simulation.

The strengths of the manuscript lies in its scope of performing large scale simulations, and seeing the effect of the crowded environment on protein structure. Validations are drawn from binding assays and structural biology experiments, which always adds value to computational works.

I have a number of concerns nonetheless:

1. first, from the mean first passage times it look like the transition times, namely tilting or opening is of the order of microseconds. If multi-replica single-protein simulations were performed for this long, would that have captured similar transition times, or is it actually the confinement that is imposing this microsecond-scale kinetics ? I suggest that the authors try this control experiment, and show that some of the reported transitions cannot be seen in a long sub-microsecond single-copy simulation of HA or NA. This would really strengthen the significance of the connectivity analysis shown at the end.

2. My next question is about verifying the theoretical applicability of MSMs in the current context. There is an inherent correlation time associated with the fluctuating inter-protein connectivities. I think, the only way a whole-system simulation can be assumed as an aggregate of monomeric sampling if the timescale of inter-monomeric correlation is much smaller than that of the intra-monomeric changes. Under this condition, the confinement can be assumed essentially a continuum or mean-field (with no net structure) within which a protein tilts, opens or breathes. If however, the correlation of the intra vs inter-protein dynamics happen on a comparable timescales, an additional potentially ‘non-Markovian’ memory term gets added to the treatment impacting all the kinetic estimates. So I suggest to discuss this issue and verify the applicability of the Markovian Assumption as it is a key element of the manuscript.

3. The antibody mapping on the ‘underbelly’ of NA is powerful showcase of molecular modeling. If possible, I would heavily suggest a rigid-body Brownian Dynamics simulation to show some explicit, understandably approximate, binding poses.

With these points addresses, I will be happy to revisit the manuscript.

Author's Response to Peer Review Comments:

Response to the reviewers

Reviewer: 1

Recommendation: Publish in ACS Central Science without change.

Comments:
Amaro and co-workers present a tour-de-force molecular simulation study of the influenza virion which provides insights on the viral egress process via characterisation of specific dynamic modes.

The study builds upon previously published work from this group on influenza which provides confidence in their knowledge and handling of this system, and extends it to look at two evolutionarily linked viruses.

Markov state modelling is used to probe the system kinetics. Extensive supplementary information is provided to further support the conclusions of the work.

I commend the authors on an excellent study. Further, i think these types of simulations of large systems, which are carried out rigorously and with due consideration and discussion of caveats etc are essential for moving the field forward.

Minor comment:

Can Figure 1 be simplified at all? Its a bit too busy imho.

Response: We thank the Reviewer for the positive feedback on our work. We also appreciate and value the comment about Figure 1 on possibly being too busy in the Reviewer's opinion. According to this comment, we scrutinized the information contained in Figure 1. We concluded that all the panels presented in Figure 1 are fundamental to illustrate the main features of influenza virus: its shape and the glycoprotein distribution (central panel), the main influenza proteins embedded into the lipid envelope (top-left: NA; bottom-left: HA; top-right: M2), and the glycans (bottom right). We also believe that it is critical to include information about the glycosylation sites (yellow text) since some of these are mentioned throughout the text. Moreover, considering that the simulations are performed at the mesoscale we believe that it is necessary to report the scale bars to illustrate the different spatial scales encompassed by our simulations. In conclusion, the only elements that we decided to remove from Figure 1 are the numbers under the "HA," "NA" and "M2" labels indicating the number glycoprotein copies included in our model since they are already mentioned in the text. As requested by the Reviewer, this has made the image a little less busy. We have now included the modified image in the revised version of the manuscript.

Reviewer: 2

Recommendation: Reconsider after major revisions noted.

The manuscript by Casalino and team describes all-atom molecular dynamics simulation of two solvated influenza A virion models. The data is analyzed using Markov State Models to study conformational transitions of the surface proteins, and seek the biological relevance of the hidden conformations seen in the MD simulation.

The strengths of the manuscript lies in its scope of performing large scale simulations, and seeing the effect of the crowded environment on protein structure. Validations are drawn from binding assays and structural biology experiments, which always adds value to computational works.

Response: We thank the Reviewer for highlighting the strengths of our work and for the positive feedback.

I have a number of concerns nonetheless:

1. first, from the mean first passage times it look like the transition times, namely tilting or opening is of the order of microseconds. If multi-replica single-protein simulations were performed for this long, would that have captured similar transition times, or is it actually the confinement that is imposing this microsecond-scale kinetics?

Response: We thank the Reviewer for raising this important point that we are glad to clarify. First, we would like to point out that the estimated mean first passage times (MFPTs) for the NA head tilting (untilted-to-tilted transition, Fig. 2) and HA head breathing (closed-to-open transition, Fig. 4) are not in the order of microseconds as mentioned by the Reviewer, but rather in the order of nanoseconds (or sub-microseconds), namely ~400 ns the former and ~300 ns the latter. These MFPTs are even shorter than the length of the performed whole-virion simulations (~440 ns). In fact, only the MFPT for the HA ectodomain tilt (untilted-to-tilted transition, Fig. 3) is in the order of microseconds, namely ~1.2 µs. In addition, we would like to clarify that our goal is not to compare the motion kinetics obtained from the whole-virion simulations with the kinetics of the same motions obtained from a simulation of a single glycoprotein in a water box. Although it can provide valuable insights, the single glycoprotein regime is a simplified setup as it only accounts for one limited scenario that, considering the glycoprotein patterning shown by cryo-ET experiments (Harris et al. *PNAS* 2006, https://doi.org/10.1073/pnas.0607614103) and reproduced in our mesoscale model, is less frequent in influenza virus.

As highlighted by the Reviewer, one strength of our simulation is that they account for the whole influenza virus. The geometrical information of our model, like the shape of the virion, as well as the morphological information, such as the number, the distribution, and the patterning of the HA and NA glycoproteins in the lipid envelope, are taken from cryo-ET experiments (Harris et al. *PNAS* 2006, https://doi.org/10.1073/pnas.0607614103). As such, with some approximations, our mesoscale model realistically reproduces the crowded subcellular environment of the influenza virus, with protein crowding being a morphological feature of this environment. In this environment, HA's and NA's ectodomains are not restricted to a small region by a fixed physical barrier. Therefore, they are not limited to exerting molecular motions, such as tilting, by protein confinement per definition (Huan-Xiang Zhou et al. *Annu. Rev. Biophys*. 2008, PMID: 18573087). In fact, they are not even fully enclosed by neighboring glycoproteins in most cases. In addition, surrounding glycoproteins are often at a loose distance, allowing HA's and NA's ectodomains to move and wiggle through the gaps. However, when at a very close distance from each other, i.e., when interacting with each other, glycoproteins can provide a confining boundary to each other owing to the fact that they are tethered to the membrane. Yet, the case where HA is fully surrounded, i.e., enclosed all-around by neighboring glycoproteins at interaction distance (< 5 Å), is rare.

In order to address the Reviewer's concern, we inquired whether this type of confinement could have an impact on the HA ectodomain tilting motion. In order to do so we calculated the correlation between (i) the HA ectodomain-tilt angle swing range (calculated as *maximum angle value – minimum angle value*) exhibited by the 236 HAs and (ii) the respective average number of connections made with surrounding glycoproteins during the simulations. The second metric should approximate the average confinement that each HA faces along the dynamics: more average connections imply more average confinement; fewer or zero connections imply less confinement. As a

result of this analysis, the two metrics are weakly, negatively correlated, with a Pearson coefficient (r) of –0.23 in the case of H1N1-Shan2009 (panels A and C) and –0.15 in the case of 2015-Mich2015 (panel B and D).

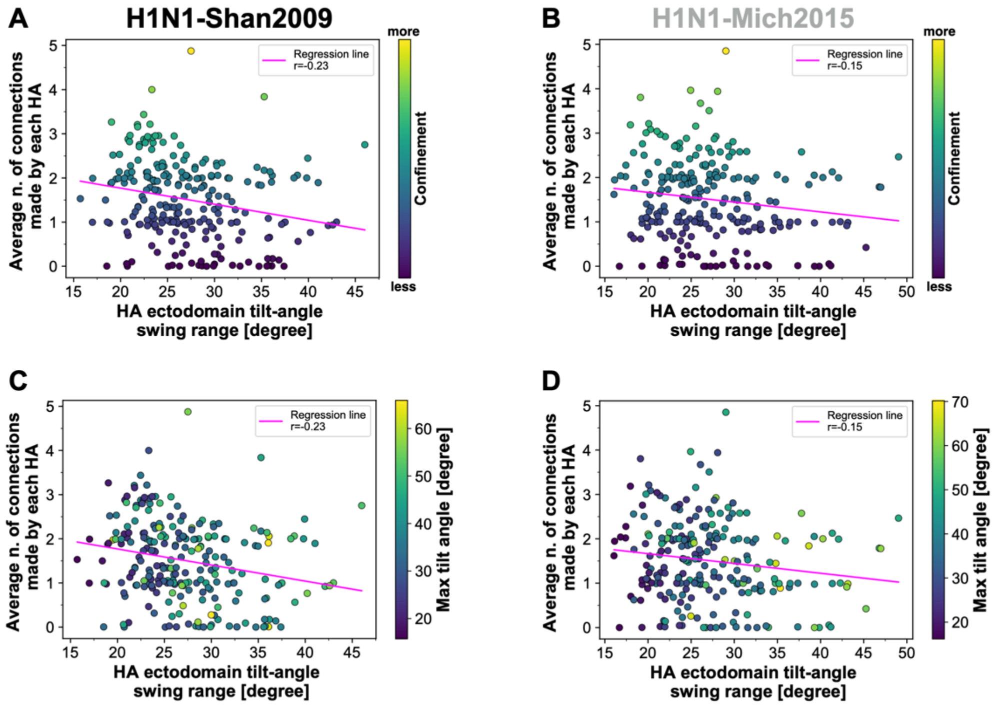

From the plots above, it emerges that among the 236 HAs copies, only a few HAs establish, on average, during both simulations, more than 3 connections with neighboring glycoproteins. Despite being tightly confined along the simulations, these few HAs still exhibit remarkable (~25º) tilt-angle swing ranges in both systems (panels A and B), and some of them even show remarkable (i.e., larger than 40º) maximum tilt-angle values (panels C and D). Interestingly, the largest tilting-angle swing range calculated in the H1N1-Shan2009 simulation (> 45 º) is exhibited by one moderately confined HA protein (panel A). This particular HA is surrounded by two HAs and one NA, with an average number of connections of 2.75 during the simulation. Nonetheless, during the 440 ns of the H1N1-Shan2009 simulation, its ectodomain tilting angle ranges from 6º (untilted) to 52º (tilted), marking a 46º excursion. Notably, there is only one HA that is constantly enclosed all around by neighboring glycoproteins at interaction distance (< 5 Å), showing an average of 5 connections along the simulations. Yet, this HA exhibits a moderate tilt-angle swing range (~27 º).

I suggest that the authors try this control experiment, and show that some of the reported transitions cannot be seen in a long sub-microsecond single-copy simulation of HA or NA. This would really strengthen the significance of the connectivity analysis shown at the end.

Response: from the plots above (panels C and D) we have extracted the HAs that do not interact with any neighboring proteins during the simulation, i.e., the HAs that make an average of 0 to 0.03 connections along the dynamics, where 0.03 was chosen as high threshold value accounting for a few isolated frames where a connection was established. Therefore, the selected subset of HAs only accounts for the HAs that remain isolated all the time (or most of it), with no other glycoproteins lying within 5 Å of them. This limited scenario approximates to a certain extent the one of a single-protein simulation of HA. The results of this analysis are shown in panels E and F shown below. In H1N1-Shan2009 (panel E), only one out of 16 HAs reaches the tilted state (tilting angle > 50º). In H1N1-Mich2015 (panel F), only 3 out of 17 reach the tilted state. Most of the isolated HAs remain in the untilted or slightly tilted state despite not being confined by any other protein, pinpointing that longer sampling than ~0.5 µs could be necessary to see the full transition to the tilted state.

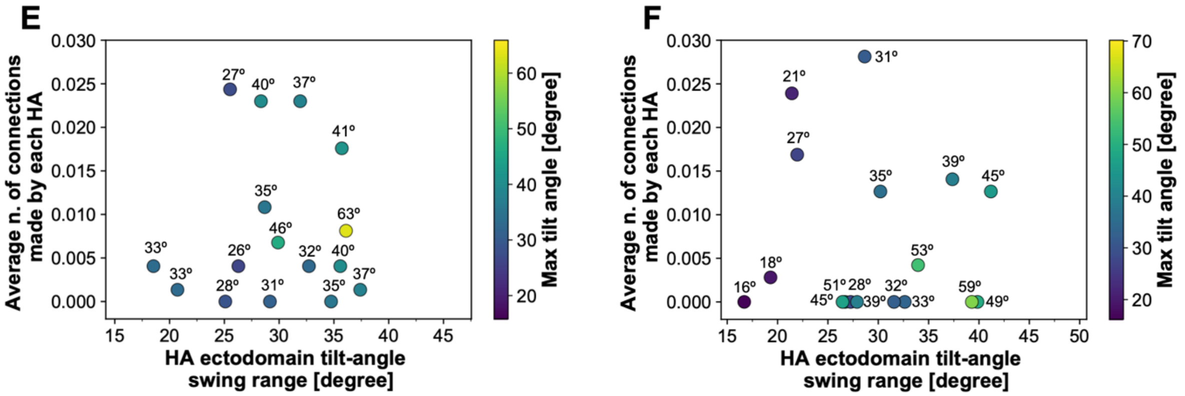

In conclusion, we concur that protein confinement can occur when two glycoproteins are at a very close distance from each other. Although there is indeed a negative correlation between the tilting swing range and the average protein confinement, this is very weak (r = -0.23 and –0.15). Hence, the microsecond-scale kinetics extrapolated for the HA tilting motion does not seem to be strictly imposed by the protein confinement. In addition, almost all the isolated HAs do not exhibit the full transition in the sub-microsecond time scale.

In the revised version of the manuscript, we have recalculated the MFPTs for each MSM and included the error as a standard deviation. Then we added a paragraph in the Results section on page 12 of the revised manuscript addressing the question about protein confinement. The relative plots with the correlation analyses (plots A to F) have been added to the revised version of the Supporting Information (Figure S20).

2. My next question is about verifying the theoretical applicability of MSMs in the current context. There is an inherent correlation time associated with the fluctuating inter-protein connectivities. I think, the only way a whole-system simulation can be assumed as an aggregate of monomeric sampling if the timescale of inter-monomeric correlation is much smaller than that of the intra-monomeric changes. Under this condition, the confinement can be assumed essentially a continuum or mean-field (with no net structure) within which a protein tilts, opens or breathes. If however, the correlation of the intra vs inter-protein dynamics happen on a comparable timescales, an additional potentially 'non-Markovian' memory term gets added to the treatment impacting all the kinetic estimates. So I suggest to discuss this issue and verify the applicability of the Markovian Assumption as it is a key element of the manuscript.

Response: We thank the reviewer for giving us the opportunity to discuss the theoretical applicability of MSMs in the context of the mesoscale simulations presented here.

First, we would like to clarify that the MSMs built in our work are based on aggregates of trimeric (for HA) / tetrameric (for NA) sampling and not monomeric sampling. HA is a homo-trimer, whereas NA is a homo-tetramer. In our model, there are 236 HA homo-trimers, which generated 236 trimeric trajectories, and 30 NA homo-tetramers, which generated 30 tetrameric trajectories. These individual trajectories have been loaded into pyEMMA to build the MSMs presented here. We apologize if the first paragraph in the Results section has created any confusion about this point. We have now clarified this point in the revised version of the Manuscript.

With our treatment, i.e., aggregating all the individual trimeric/tetrameric trajectories to build a Markov State Model, only a subset of system coordinates is explicitly accounted for, whereas other components of the entire set of the system coordinates, such as, for example, the inter-protein connectivity, are only implicitly included. Therefore, we concur with the Reviewer that an implicit treatment of such components could potentially undermine the Markovianity of the dynamics described by our MSMs. It is also true that the conditions for strict Markovian dynamics are usually very hard to achieve when dealing with complex biological systems. As mentioned in Suárez et al. JCTC 2016 (https://doi.org/10.1021/acs.jctc.6b00339), "*even when the underlying dynamics of a continuous system (e.g., molecular) is Markovian, the system's behavior becomes non-Markovian when projected onto a finite discrete space…The issue becomes more pronounced when projecting from a high-dimensional space to a relatively small number of discrete states.*" An interesting article (https://doi.org/10.6083/M4736QFT) written by Dan Zuckerman,

one of the experts in the field, highlights how "*everything is Markovian,*" like, for example, stochastic and deterministic dynamics, but at the same time, "*nothing is Markovian,*", because when only a subset of the system's phase space is considered for the MSM analysis, the system behavior will appear non-Markovian. This is often the case for complex systems like the one presented here, where it is routine and sometimes a necessity to operate on sub-spaces of the phase space. In practice, all treatments that consider only a subset of the system's phase space could lead to the introduction of non-Markovian memory terms due to the other components that are not explicitly considered. Demonstrating that the Markov approximation is sufficiently accurate is not trivial, as this remains an ongoing topic of research.

The Reviewer specifically mentioned the inter-protein connectivity as a potential component that could introduce a non-Markovian term to our description, inviting us to discuss if our approach could be applicable in this context. To address the Reviewer's concern regarding this point, for each MSM presented in our work, i.e., NA head tilting, HA ectodomain tilting, and HA head breathing, we calculated the conditional probability that a glycoprotein (either NA or HA) exhibits a certain degree of tilting/breathing during the dynamics given that it forms a connection with one or more neighboring glycoproteins. Here the conditional event, i.e., the formation of one or more connections during the dynamics, should account for the inter-protein connectivity mentioned by the Reviewer. Therefore, with this analysis, we investigate whether the inter-protein connectivity somehow affects the probability of achieving a certain degree of tilting/breathing. In order to do so, we used the following formula:

$$\mathbf{P(A|B) = P(A \cap B) / P(B)}$$

Where:

- **P(A)** is the probability that a glycoprotein tilts/breathes by a certain amount during the dynamics with respect to the initial frame.
- **P(B)** is the probability that the glycoprotein forms one or more connections during the dynamics
- **P(A∩B)** is the probability at which the events A and B occur together.
- **P(A|B)** is the probability of event A occurring if event B has happened.

The rationale of this analysis is that if **P(A∩B)**) ≈ **P(A)**, then the inter-protein connectivity does not affect the probability of tilting/breathing by a certain amount along the simulation, and ultimately the untilted-to-tilted transitions or closed-to-open transition described by our MSMs.

The results of this analysis, performed for both the simulated systems, are presented in the tables below:

Table 1. P(A|B) for NA head tilting, where event A is the achievement of a certain tilt-angle swing range by the NA head during the dynamics with respect to the initial frame (calculated as *tilt-angle_frame_n – tilt-angle_frame_0*, with only positive values counted), whereas event B is the formation of one or more connections during the dynamics.

	**H1N-Shan2009**		**H1N-Mich2015**	
**EVENTS**	**P(A)**	**P(A|B)**	**P(A)**	**P(A|B)**
**A:** 0º ≤ **NA** head tilt-angle swing range < 10º<br>**B:** 1 or more connections	32.1 ± 0.4 %	34.6 ± 0.7 %	29.9 ± 0.4 %	27.5 ± 0.6 %
**A:** 10º ≤ **NA** head tilt-angle swing range < 20º<br>**B:** 1 or more connections	18.9 ± 0.3%	18.0 ± 0.5 %	14.3 ± 0.3 %	18.8 ± 0.5 %
**A:** 20º ≤ **NA** head tilt-angle swing range < 20º<br>**B:** 1 or more connections	6.0 ± 0.2 %	4.9 ± 0.2 %	6.5 ± 0.2 %	8.2 ± 0.3 %
**A:** 30º ≤ **NA** head tilt-angle swing range < 40º<br>**B:** 1 or more connections	2.4 ± 0.1 %	2.5 ± 0.2 %	6.9 ± 0.2 %	8.0 ± 0.3 %
**A:** 40º ≤ **NA** head tilt-angle swing range<br>**B:** 1 or more connections	1.8 ± 0.1 %	2.0 ± 0.2 %	1.2 ± 0.1 %	0.9 ± 0.1 %

Table 2. P(A|B) for HA ectodomain tilting, where event A is the achievement of a certain tilt-angle swing range by the HA ectodomain during the dynamics with respect to the initial frame (calculated as *tilt-angle_frame_n – tilt-angle_frame_0*, with only positive values counted), whereas event B is the formation of one or more connections during the dynamics.

	H1N-Shan2009		H1N-Mich2015	
**EVENTS**	**P(A)**	**P(A|B)**	**P(A)**	**P(A|B)**
**A:** 0º ≤ **HA** tilt-angle swing range < 10º<br>**B** = 1 or more connections	45.2 ± 0.2 %	44.6 ± 0.3 %	45.8 ± 0.2 %	45.7 ± 0.3 %
**A:** 10º ≤ **HA** tilt-angle swing range < 20º<br>**B** = 1 or more connections	19.2 ± 0.1 %	19.8 ± 0.2 %	17.6 ± 0.1 %	18.5 ± 0.2 %
**A:** 20º ≤ **HA** tilt-angle swing range < 30º<br>**B** = 1 or more connections	3.9 ± 0.0 %	4.3 ± 0.1 %	4.4 ± 0.1 %	4.5 ± 0.1 %
**A:** 30º ≤ **HA** tilt-angle swing range<br>**B** = 1 or more connections	0.3 ± 0.0 %	0.4 ± 0.0 %	0.6 ± 0.0 %	0.6 ± 0.0 %

Table 3. P(A|B) for HA breathing head tilting, where event A is the achievement of a certain extent of breathing by at least one of the HA head monomer within the same HA trimer during the dynamics with respect to the initial frame (calculated as *breathingdistance_frame_n – breathingdistance_frame_0*, with only positive values counted), whereas event B is the formation of one or more connections during the dynamics.

	H1N-Shan2009		H1N-Mich2015	
**EVENTS**	**P(A)**	**P(A|B)**	**P(A)**	**P(A|B)**
**A:** 1.0 Å ≤ breathing of one HA head < 3.0 Åº<br>**B:** 1 or more connections	46.9 ± 0.2 %	46.7 ± 0.3%	46.2 ± 0.2 %	46.1 ± 0.3%
**A:** 3.0 Å ≤ breathing of one HA head < 5.0 Åº<br>**B:** 1 or more connections	11.2 ± 0.1 %	11.5 ± 0.1 %	11.0 ± 0.1 %	10.9 ± 0.1 %
**A:** 5.0 Å ≤ breathing of one HA head < 7.0 Åº<br>**B:** 1 or more connections	2.2 ± 0.0 %	2.4 ± 0.1 %	2.8 ± 0.0 %	2.8 ± 0.1 %
**A:** 7.0 Å ≤ breathing of one HA head<br>**B:** 1 or more connections	0.2 ± 0.0 %	0.2 ± 0.0 %	0.3 ± 0.0 %	0.3 ± 0.0 %

For all the three investigated motions, i.e., NA head tilting (Table 1), HA ectodomain tilting (Table2), and HA head breathing (Table 3), **P(A∩B))** is similar to **P(A)** in both systems. Especially for HA ectodomain tilting and HA head breathing, **P(A∩B))** is almost right on the dot equal to **P(A).** These results pinpoint that the inter-protein connectivity does not affect the probability of tilting or breathing of NA and HA. Therefore, although the inter-protein connectivity is not explicitly accounted for in our treatment when concatenating all the individual trajectories, the Markovian approximation of our models should hold. Moreover, we remark that all our MSMs were validated through CK tests, as detailed in the Supporting Information.

In addition, we have also calculated the inverted conditional probability P(B|A), i.e., we calculated the probability that a glycoprotein (either NA or HA) forms a connection with one or more glycoproteins given that it exerts a certain degree of tilting/breathing during the dynamics:

$$\mathbf{P(B|A) = P(B \cap A) / P(A)}$$

The results of this analysis, performed for both the simulated systems, are presented in the tables below:

Table 4. P(B|A) for NA head tilting, where event A is the achievement of a certain tilt-angle swing range by the NA head during the dynamics with respect to the initial frame (calculated as *tilt-angle_frame_n – tilt-angle_frame_0*, with only positive values counted), whereas event B is the formation of one or more connections during the dynamics.

	H1N-Shan2009		H1N-Mich2015	
**EVENTS**	**P(B)**	**P(B|A)**	**P(B)**	**P(B|A)**
**A:** 0º ≤ **NA** head tilt-angle swing range < 10º<br>**B:** 1 or more connections	65.8 ± 0.5 %	71.1 ± 1.4 %	56.6 ± 0.4 %	52.1 ± 1.2 %
**A:** 10º ≤ **NA** head tilt-angle swing range < 20º<br>**B:** 1 or more connections	65.8 ± 0.5 %	62.5 ± 1.6 %	56.6 ± 0.5 %	74.3 ± 2.2 %
**A:** 20º ≤ **NA** head tilt-angle swing range < 20º<br>**B:** 1 or more connections	65.8 ± 0.5 %	54.3 ± 2.6 %	56.6 ± 0.5 %	70.8 ± 3.0 %
**A:** 30º ≤ **NA** head tilt-angle swing range < 40º	65.8 ± 0.5 %	67.1 ± 4.6 %	56.6 ± 0.5 %	65.5 ± 2.8 %

**B:** 1 or more connections				
**A:** 40º ≤ **NA** head tilt-angle swing range<br>**B:** 1 or more connections	65.8 ± 0.5 %	73.5 ± 5.6 %	56.6 ± 0.5 %	45.0 ± 5.2 %

Table 5. P(B|A) for HA ectodomain tilting, where event A is the achievement of a certain tilt-angle swing range by the HA ectodomain during the dynamics with respect to the initial frame (calculated as *tilt-angle_frame_n – tilt-angle_frame_0*, with only positive values counted), whereas event B is the formation of one or more connections during the dynamics.

	**H1N-Shan2009**		**H1N-Mich2015**	
**EVENTS**	**P(B)**	**P(B|A)**	**P(B)**	**P(B|A)**
**A:** 10º ≤ **HA** tilt-angle swing range < 20º<br>**B:** 1 or more connections	82.9 ± 0.2 %	81.8 ± 0.5 %	83.4 ± 0.2 %	83.1 ± 0.5 %
**A:** 10º ≤ **HA** tilt-angle swing range < 20º<br>**B:** 1 or more connections	82.9 ± 0.2 %	85.5 ± 0.7 %	83.4 ± 0.2 %	87.8 ± 0.8 %
**A:** 20º ≤ **HA** tilt-angle swing range < 30º<br>**B:** 1 or more connections	82.9 ± 0.2 %	91.1 ± 1.6 %	83.4 ± 0.2 %	85.5 ± 1.5 %
**A:** 30º ≤ **HA** tilt-angle swing range<br>**B:** 1 or more connections	82.9 ± 0.2 %	99.8 ± 5.8 %	83.4 ± 0.2 %	95.1 ± 4.4 %

Table 6. P(B|A) for HA breathing head tilting, where event A is the achievement of a certain extent of breathing by at least one of the HA head monomer within the same HA trimer during the dynamics with respect to the initial frame (calculated as *breathingdistance_frame_n – breathingdistance_frame_0*, with only positive values counted), whereas event B is the formation of one or more connections during the dynamics.

	**H1N-Shan2009**		**H1N-Mich2015**	
**EVENTS**	**P(A)**	**P(B|A)**	**P(A)**	**P(B|A)**
**A:** 1.0 Å ≤ breathing of one HA head < 3.0 Åº<br>**B:** 1 or more connections	82.9 ± 0.2 %	82.5 ± 0.5 %	83.4 ± 0.2 %	83.2 ± 0.5 %
**A:** 3.0 Å ≤ breathing of one HA head < 5.0 Åº<br>**B:** 1 or more connections	82.9 ± 0.2 %	85.2 ± 0.9 %	83.4 ± 0.2 %	82.8 ± 0.9 %
**A:** 5.0 Å ≤ breathing of one HA head < 7.0 Åº<br>**B:** 1 or more connections	82.9 ± 0.2 %	91.5 ± 2.2 %	83.4 ± 0.2 %	84.5 ± 1.8 %
**A:** 7.0 Å ≤ breathing of one HA head<br>**B:** 1 or more connections	82.9 ± 0.2 %	97.1 ± 7.4 %	83.4 ± 0.2 %	81.8 ± 5.3 %

For all the three investigated motions, i.e., NA head tilting (Table 4), HA ectodomain tilting (Table 5), and HA head breathing (Table 6), **P(B∩A)**) is not similar to **P(B)** in both systems. Especially for the HA ectodomain tilting (both H1N1-Shan2009 and H1N1-Mich2015) and HA head breathing in H1N1-Shan2009, **P(A∩B)**) is larger than P(B) for the higher range of values of event A. These results pinpoint that extensive HA ectodomain tilting and HA head breathing increase the probability of forming one more connection for HA, boosting the interplay. Instead, a less defined behavior is shown by NA, where for certain ranges of values of head tilt the interplay is boosted, whereas for other ranges is instead reduced.

We thank again the Reviewer for giving us the possibility to discuss this important point. We believe that by showing that the probability that HA and NA tilt and breathe to a certain extent is not affected by the inter-protein connectivity, the Markovian assumption of the presented MSMs holds. We remark that all our MSMs were validated through CK tests, as already detailed in the Supporting Information. We have included the description of this analysis and added the relative Tables (S4 to S9) to the revised version of the Supporting Information, and we have added a few sentences commenting on this analysis in the Results section (page 19) and in the Materials and Methods section (page 29) of the revised version of the Manuscript.

3. The antibody mapping on the 'underbelly' of NA is powerful showcase of molecular modeling. If possible, I would heavily suggest a rigid-body Brownian Dynamics simulation to show some explicit, understandably approximate, binding poses.

Response: We thank the Reviewer for the suggestion. We agree that it would be insightful to provide more structural details on the binding pose of the NDS.1 Fab to the underside of the NA head. Unfortunately, this is not possible as the resolution of the NDS.1 Fab particles is only ~24 Å, too low to resolve the structure of the antibody.

Any attempt to guess a more accurate binding pose, perhaps using docking calculations with a mock Fab in place of NDS.1, would lead to misleading results since the specificity of the NDS.1's variable domain would not be accounted for. Therefore, in order to provide a better understanding of where NDS.1 binds, we have fitted a mock human Fab from (PDB ID: 7FAB) into the density of NDS.1 and included the pose as a new Figure in the revised version of the Supporting Information.

Considering the scope of this work, we believe that Figure 2F, Figure S16, and the newly added figure (Figure S17) to the revised version of the Supporting Information suffice to show the portion of the NA head region targeted by NDS.1 Fab. A sentence referencing Figure S17 was added in the Results section of the revised Manuscript at the end of page 9.

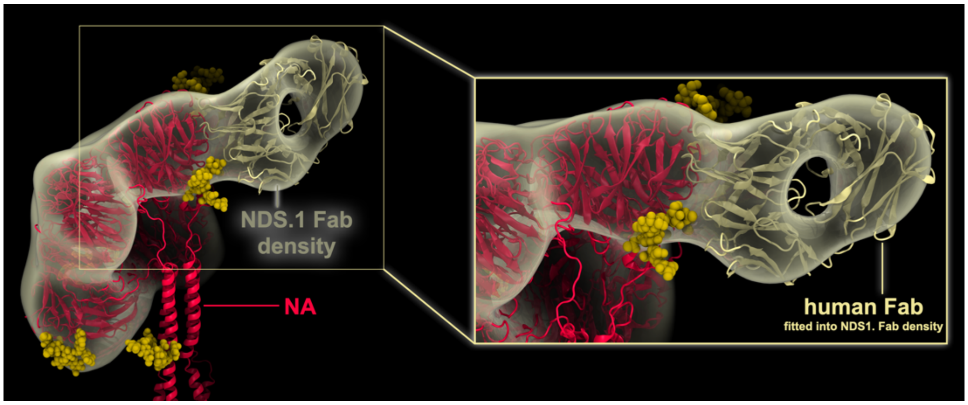

Supplement: Supplementary file 12 — oc2c00981_si_013.pdf [file oc2c00981_si_013.pdf]
